# Tailoring exciplex formation in metal-induced supramolecular organization

**DOI:** 10.1039/d6dt00490c

**Published:** 2026-04-06

**Authors:** Giau Le-Hoang, Laure Guénée, Claude Piguet

**Affiliations:** a Department of Inorganic and Analytical Chemistry, University of Geneva 30 quai E. Ansermet CH-1211 Geneva 4 Switzerland claude.piguet@unige.ch hoang.le@unige.ch; b Laboratory of Crystallography, University of Geneva 24 quai E. Ansermet CH-1211 Geneva 4 Switzerland

## Abstract

The intramolecular π-polarization induced upon binding the bichromophoric ligand L1 to lanthanide ions produces intermolecular hetero-π-stacked pyrene–terimine organization in the resulting complexes as ascertained by ground state supramolecular aggregations leading to dimeric [L1Ln(hfac)_3_]_2_ (Ln = Y, Eu) units both in the solid state (X-ray crystal structure determination) and in dichloromethane solution (^1^H NMR titrations). Photoexcitation engenders additional polarization changes which further complicate/extend interaromatic aggregation processes *via* the formation of light-driven excimers and exciplexes. The various π-stacking intermolecular interactions operating in the [L1Y(hfac)_3_] host are probed by reaction with three competitive aromatics guests: electron-poor nitrobenzene, neutral benzene and electron-rich methoxybenzene.

## Abstract 2

The binding of lanthanide ions to bichromophoric organic ligands leads to a ground state and photo-driven excited state hetero-dimeric organization.

## Graphical abstract

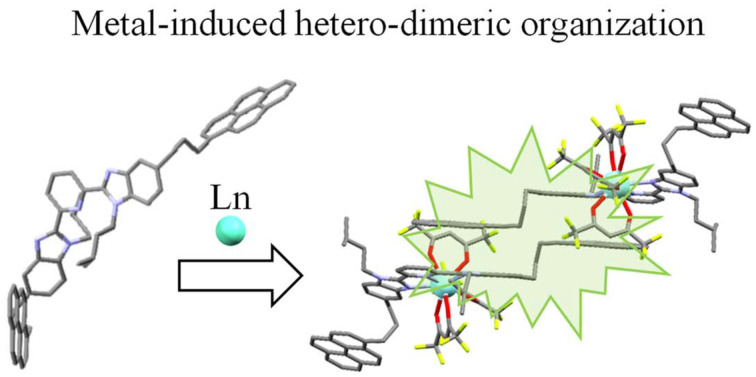

## Introduction

**Fig. 1:**
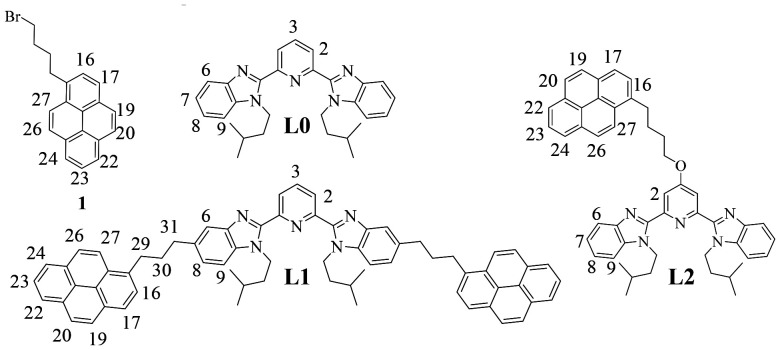
Chemical structures of the target ligands studied in this work, with numbering schemes used for ^1^H NMR measurements.

In 1990, Sanders and Hunter proposed a simple and highly welcome electrostatic model to rationalize the non-covalent interactions operating between aromatic systems in which each neutral sp^2^ carbon carries one positive *δ* = +1 charge located on the skeleton, whereas two negative π = −½ charges lie above and below the plane of the aromatic system.^1^ A favourable stacking interaction between two aromatic rings then operates when the δ↔π attraction dominates over the π↔π and/or δ↔δ repulsion. Based on this model, for an unpolarized π system, the stacking interaction between a neutral aromatic system and an electron-rich aromatic system is less favourable than that between two neutral units, while the contact between a neutral aromatic ring and an electron-poor aromatic ring is the most efficient due to the minimized π↔π repulsion.^1,2^ Modulation of π-polarization within aromatic frameworks in their ground or excited states for tuning the nature, scale and geometry of π-stacking remains of great interest to organic and physical chemists eager to rationally program the chemical and physical properties of the resulting molecular/supramolecular assemblies.^3–5^ In this context, pyrene is a well-known planar conjugated neutral polyaromatic chromophore, for which its strong monomer fluorescence (350–400 nm) upon light excitation can be completed with a red-shifted excimer emission (480 nm) generated by dimerization between an excited-state pyrene molecule and a ground-state pyrene molecule *via* non-covalent π-stacking interactions at high monomer concentrations.^6,7^ This photochemical process may also occur between two different aromatic molecules, such as pyrene and 1,2-dimethylindole, or pyrene and *N*,*N*-dimethylaniline, resulting in exciplex emissions.^8–10^ The transformation of pyrene monomer fluorescence into aggregation-induced excimer/exciplex emission is strongly influenced by multifactorial parameters including chromophore concentration, solvent polarity and viscosity, temperature, light, local environment, aromatic guests, quenchers, spatial orientation and electronic densities of the pyrene/aromatic units.^11–13^ The sensitivity of pyrene-based fluorescence to these factors allows control over its emission characteristics (intensity, wavelength, lifetime), thereby enabling its applications as biochemical sensors and as optoelectronic materials *via* the construction or the destruction of the excited dimers and the modulation of its excimer emission.^14–19^ Most of these applications require very dilute concentrations of pyrene-containing materials, while the dimerization of polyaromatic organic compounds occurs typically at moderate or high concentrations approaching 10^−3^–10^−2^ M. Therefore, controlling and boosting excimer/exciplex formation at low concentrations by molecular design continues to attract the attention of organic chemists (Fig. 1).

In this study, we report a rare example where a lanthanide ion is used for selectively inducing intermolecular hetero-π-stacking interactions between a pyrene unit (1) and a bound aromatic terimine core (L0) operating in [L*k*Ln(hfac)_3_] (L*k* = L1, L2, and Ln = Eu, Y) adducts in their ground and excited states. The polarization generated upon light excitation ultimately provides exciplex emission at close to micromolar concentrations. The sensitivity of this dimeric luminescence process with respect to (i) the nature of interacting aromatic guests (electron-rich, electron-poor and neutral) and (ii) energy transfer toward the europium activator is discussed.

## Results and discussion

### Ground state aggregation

**Fig. 2:**
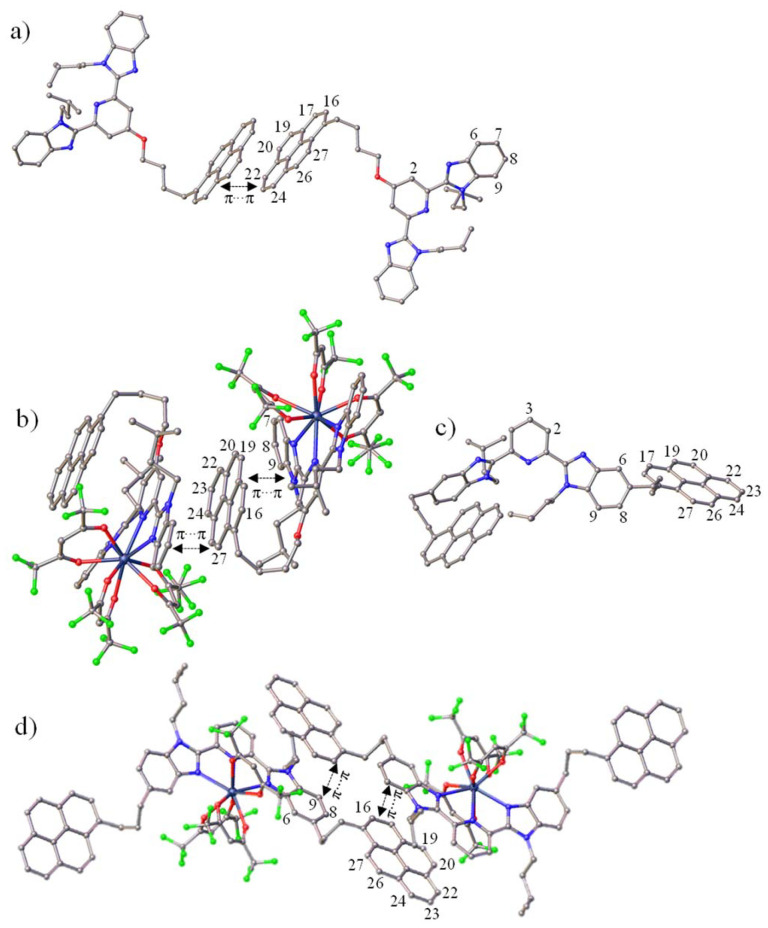
Molecular structures found in the crystal structures of (a) L2, (b) [L2Y(hfac)_3_], (c) L1, and (d) [L1Y(hfac)_3_]. Color codes: C = gray, N = light blue, O = red, F = green, Y = dark blue. Hydrogen atoms and solvent molecules have been omitted for clarity.

The syntheses of the pyrene-monomer L2, pyrene-dimer L1, and their lanthanide adducts [L*k*Ln(hfac)_3_] (L*k* = L1, L2, and Ln = Eu, Y with hfac = 1,1,1,5,5,5-hexafluoro-pentane-2,4-dione) follow published procedures^20–25^ and are detailed in Appendix 1 SI. Slow evaporation of dichloromethane/hexane solutions gave single crystals of L1, L2, [L1Eu(hfac)_3_]·0.5CH_2_Cl_2_·1.5C_6_H_14_, [L1Y(hfac)_3_]·2.25C_6_H_14_, [L2Eu(hfac)_3_], and [L2Y(hfac)_3_] suitable for X-ray analysis (Fig. 2 and Appendix 2 SI).

The crystal structure of the free ligand L2 exhibits (i) a *trans*–*trans* arrangement of two di-imine units to minimize the steric congestion between the *N*-isopentyl groups of the benzimidazole side arms and the aromatic protons of the central pyridine ring, and (ii) an intermolecular homo-π-stacking between two neutral pyrene moieties in a parallel-displaced conformation with an interplanar distance of 3.35 Å (Fig. 2a and A2-3 SI). In agreement with the electrostatic Sanders–Hunter model,^1,2^ no hetero-stacking can be detected between the neutral pyrene and the electron-rich polyaromatic terimine units in the solid state. Upon meridional tri-coordination of L2 to the lanthanide metal in [L2Ln(hfac)_3_] (Ln = Eu, Y), the bound *cis*–*cis* terimine unit becomes electron-deficient and two hetero-π-stackings, one intramolecular and one intermolecular, imply the neutral pyrene ring, which is sandwiched between two electron-poor bound terimine units in the solid state (Fig. 2b, A2-5 and A2-7 SI). Due to steric constraints, any intramolecular pyrene/polyaromatic terimine interactions are precluded in the less compact [L1Ln(hfac)_3_] complexes (Ln = Eu, Y). Only intermolecular hetero-π-stacking interactions may operate. They involve the pyrene rings and the bound polyaromatic terimine units to give [L1Ln(hfac)_3_]_2_ dimeric units within infinite packed chains in the solid state (Fig. 2d, A2-9, and A2-11 SI). Since the free ligand L1 displays no non-covalent interaction involving its aromatic units in the solid state (Fig. 2c), one concludes that productive metal-induced hetero-π-stackings may be programmed in the ground state upon complexation of our bichromophoric pyrene–terimine ligands L1 and L2 to trivalent lanthanides.

**Fig. 3:**
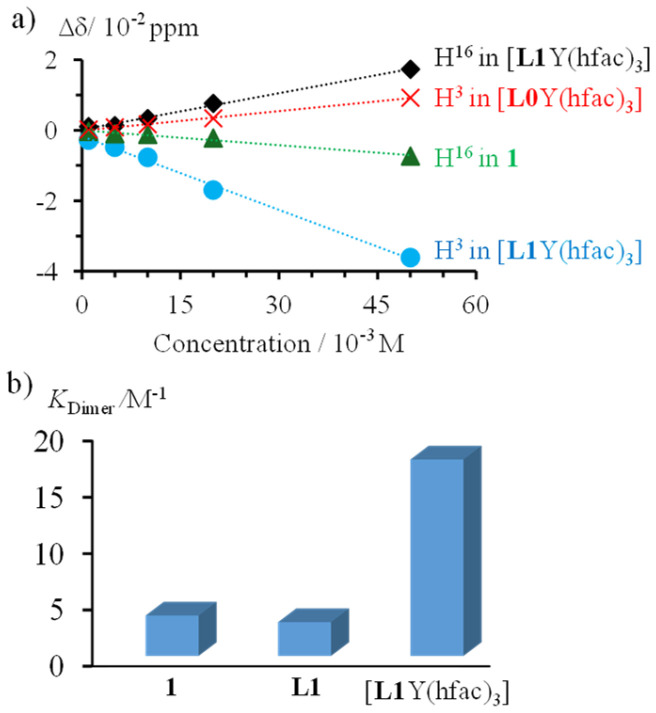
(a) Difference in ^1^H NMR chemical shifts, as compared to that recorded at 10^−4^ M, upon increasing the concentration for protons H^3^ and H^16^ in 1, [L0Y(hfac)_3_] and [L1Y(hfac)_3_], and (b) associated dimerization constants for 1, L1 and [L1Y(hfac)_3_].

**Fig. 4:**
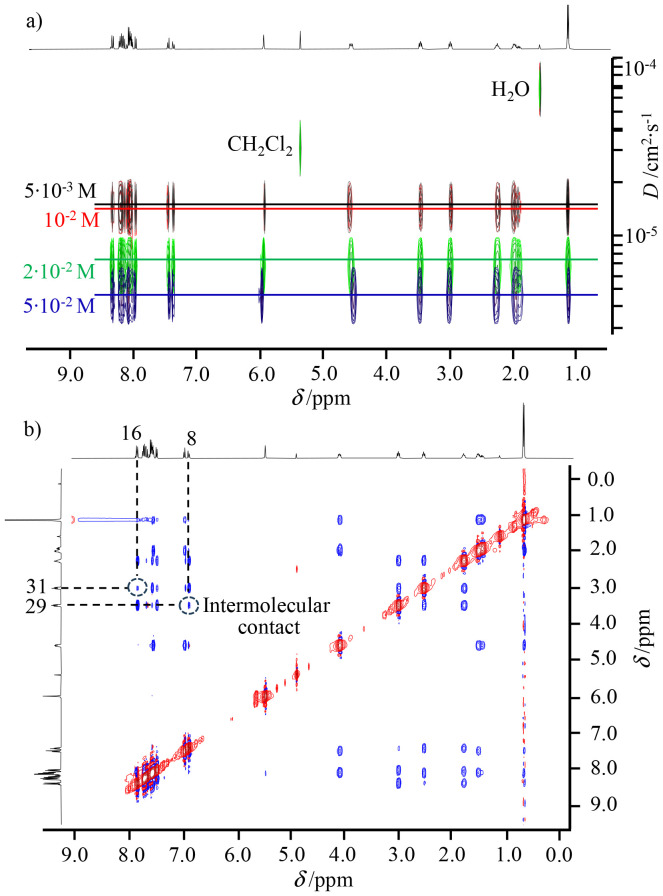
(a) DOSY spectra of [L1Y(hfac)_3_] at various concentrations, and (b) NOESY spectrum of [L1Y(hfac)_3_] at 2 × 10^−2^ M in CD_2_Cl_2_. Numbering is shown in Fig. 1.

NMR spectroscopy was employed to investigate the ground state supramolecular aggregation behaviours of metal-free 1, L0, L1, and the related yttrium complexes [L0Y(hfac)_3_] and [L1Y(hfac)_3_] in CD_2_Cl_2_ solution. The aggregation behaviour of ligand L2 is not further studied in this work due to the complexity arising from the competition between inter- and intramolecular stacking interactions found in the solid state (Fig. 2b) and will be investigated in a subsequent publication. Recording ^1^H NMR spectra at various concentrations reveals that the pyrene units in 1 (Fig. A1-20 SI) and in the free ligand L1 (Fig. A1-18 SI) exhibit faint drifts in chemical shifts in agreement with very minor dimerization (Fig. A1-18 and A1-20 SI). Similarly, the terimine cores in L0 (Fig. A1-19 SI) and in L1 (Fig. A1-18 SI) globally display invariable chemical shifts for the protons of the terimine units. Altogether, these results demonstrate that only weak, if any, intermolecular ground state π-stacking occurs between the various polyaromatic units in unbound ligands L0 and L1, even at high concentrations in CD_2_Cl_2_. The situation drastically changes upon complexation in [L1Y(hfac)_3_] (Fig. A1-17 SI). Several aromatic protons of the bound terimine moiety (for instance H^3^ or H^9^) display significant downfield shifts with increasing concentration (blue trace in Fig. 3a), whereas the corresponding signals in the reference pyrene-lacking [L0Y(hfac)_3_] complex (Fig. A1-21 SI) show opposite upfield shifts (red trace in Fig. 3a). This implies that the aromatic terimine unit is involved in an intermolecular stacking process in [L1Y(hfac)_3_], but not in [L0Y(hfac)_3_]. A related but inverse trend is observed for the aromatic protons of the pyrene unit (*i.e.* H^16^) in [L1Y(hfac)_3_] (Fig. A1-17 SI), which are shielded upon dimerization at a high concentration (black trace in Fig. 3a), whereas the corresponding signals in the reference compound 1 (Fig. A1-20 SI) are deshielded due to the well-established pyrene–pyrene aggregation process (green trace in Fig. 3a). Altogether, these results suggest the operation of a significant intermolecular hetero-π-stacked organization between the pyrene and the terimine units in [L1Y(hfac)_3_], thus reflecting that observed in the crystallized dimeric units [L1Y(hfac)_3_]_2_ (Fig. 2d). The latter intermolecular ground state aggregation behaviour of [L1Y(hfac)_3_] is further confirmed by the lower diffusion coefficients estimated using DOSY spectroscopy upon increasing the concentration of [L1Y(hfac)_3_] from 5 × 10^−3^ M to 5 × 10^−2^ M (Fig. 4a). Moreover, the NOESY spectrum of [L1Y(hfac)_3_] recorded at high concentration (≥2 × 10^−2^ M) displays specific cross peaks (H8↔H29, H16↔H31; Fig. 4b) confirming non-covalent contacts between two molecules of [L1Y(hfac)_3_], which disappear upon dilution (Fig. A1-15 SI).

A quantitative support is brought by the determination of the thermodynamic aggregation equilibrium constants *K*_Dimer_ (2M→M_2_) for 1, L1 and [L1Y(hfac)_3_] in dichloromethane solution using concentration-dependent ^1^H NMR titrations according to eqn (1) previously established by Chen and Shirts for related dimerization reactions.^26^1
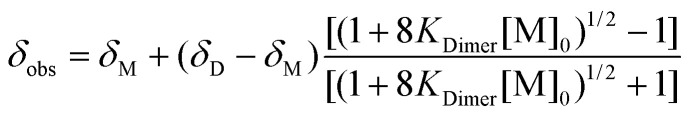
*δ*_M_ is the chemical shift of the pure monomer in a highly diluted solution, *δ*_D_ is the chemical shift of the pure dimer, *δ*_obs_ is the observed chemical shift expressed as a weighted average of *δ*_M_ and *δ*_D_ at each titration point, and [M]_0_ is the total concentration of the monomer at this point. Non-linear least-squares fits of *δ*_obs_*versus K*_Dimer_ and *δ*_D_ yielded both the dimerization constant and the chemical shift of the pure dimer (Fig. 3b and Table A3-1 SI). The pyrene unit in the [L1Y(hfac)_3_] complex displays a much higher aggregation equilibrium constant (×4) compared to those measured for the pyrene-bearing ligand L1 and for the reference compound 1, thus demonstrating the influence of the bound yttrium metal on the π-stacked supramolecular organization.

**Fig. 5:**
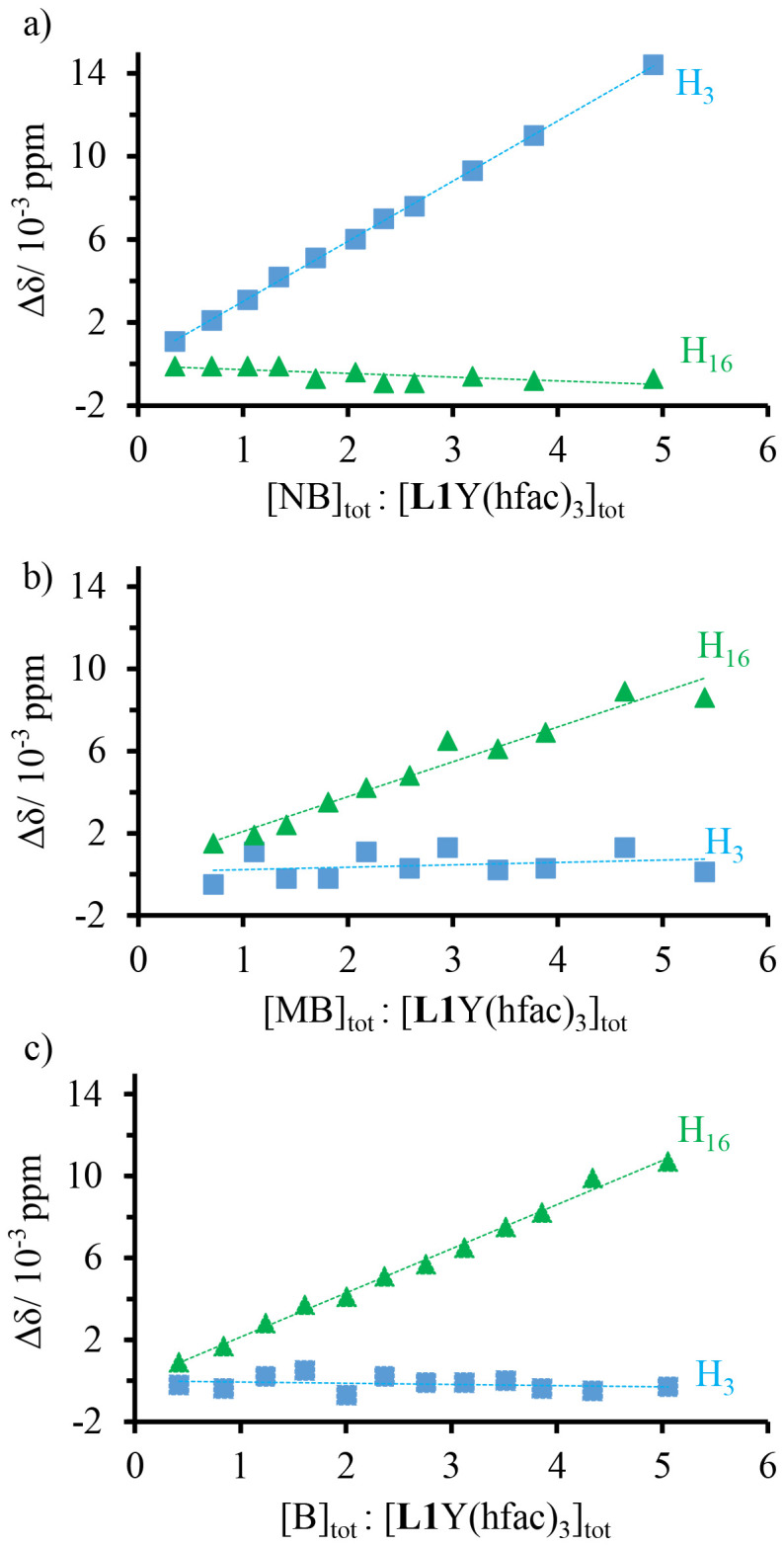
Difference in chemical shift Δ*δ* upon ^1^H NMR titration of [L1Y(hfac)_3_] with (a) nitrobenzene (NB), (b) methoxybenzene (MB), and (c) benzene (B).

Having established that the neutral pyrene rings and electron-poor aromatic terimine unit bound to the [Y(hfac)_3_] cargo are available for interaromatic π-stacking interactions in the [L1Y(hfac)_3_] complex to give dimer [L1Y(hfac)_3_]_2_ (Table 1), further competitive perturbations by external aromatic guests, including electron-rich methoxybenzene (MB), neutral benzene (B) and electron-poor nitrobenzene (NB), were investigated by ^1^H NMR titrations (Fig. 5, A4-1, A4-2, and A4-3 SI). A ^1^H NMR titration of [L1Y(hfac)_3_] (total concentration of 2.67 × 10^−2^ M, 37% of the dimer) upon addition of small aliquots of a concentrated solution of NB (1.75 M to limit dilution) displays upfield shifts of the aromatic signals of the bound terimine unit (H^3^, blue trace in Fig. 5a), whereas that of the pyrene unit displays no significant variation (H^16^, green trace in Fig. 5a). At the end of the titration, the chemical shifts of the protons of the bound terimine unit are diagnostic for the absence of π-stacking, as found for the monomeric [L1Y(hfac)_3_] at dilute concentrations (Fig. A1-17 and A4-1 SI). One concludes that the stepwise addition of electron-poor NB in solution induces competitive π-stacking interactions with the neutral pyrene unit in [L1Y(hfac)_3_], thus leading to the removal of the heteroaromatic terimine–pyrene interactions responsible for the dimerization process. In contrast, upon titrations of [L1Y(hfac)_3_] with either electron-rich MB or neutral B guests, no significant chemical shift change is observed for the protons of the terimine unit (H3, blue traces in Fig. 5b and c). The concomitant upfield shifts in the protons of the pyrene unit (H16, green traces in Fig. 5b and c) mimic that observed in Fig. 3a and thus suggest the operation of weak additional intermolecular π-stacking interactions implying MB or B, but with no significant perturbation of the heteroaromatic π-stacked pyrene–terimine framework leading to the monomer↔dimer equilibrium. Please note that the retention of the heteroaromatic π-stacking between the neutral pyrene and the electron-poor bound terimine units in [L1Y(hfac)_3_] in the presence of competitive unfavorable electron-rich MB, or neutral B, fully agrees with the Sanders–Hunter rule.

**Table 1:** Percentages of the dimeric form [L1Y(hfac)_3_]_2_ at various concentrations in CD_2_Cl_2_ (*K*_Dimer_ = 17.4)

*M* (mol L^−1^)	2 × 10^−2^	10^−2^	10^−3^	10^−4^	5 × 10^−5^	10^−5^	5 × 10^−6^
Dimer (%)	32.1	21.5	3.2	0.34	0.17	0.033	0.019

Finally, the thermodynamic formation constant of the [L1Y(hfac)_3_] complex was determined by ^1^H NMR titration of the L1 host with the [digY(hfac)_3_] guest (dig = diglyme or bis(2-methoxyethyl)) at a millimolar concentration in CD_2_Cl_2_ solution where the formation of the dimer is negligible (Table 1) and according to eqn (2):

2 [digY(hfac)_3_] + L1 ↔ [L1Y(hfac)_3_] + dig *β*^**L1**,Y^_1,1,exch_

An excess of dig (0.146 M) is used to stabilize the activity coefficients upon ^1^H NMR titration,^27^ thus transforming the exchange stability constant *β*^**L1**,Y^_1,1,exch_ into the conditional binding constant *β*^**L1**,Y^_1,1,cond_ described in eqn (3).3
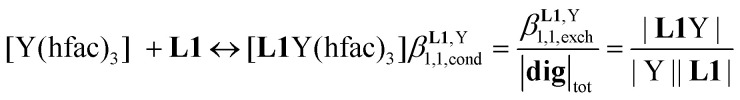


**Fig. 6:**
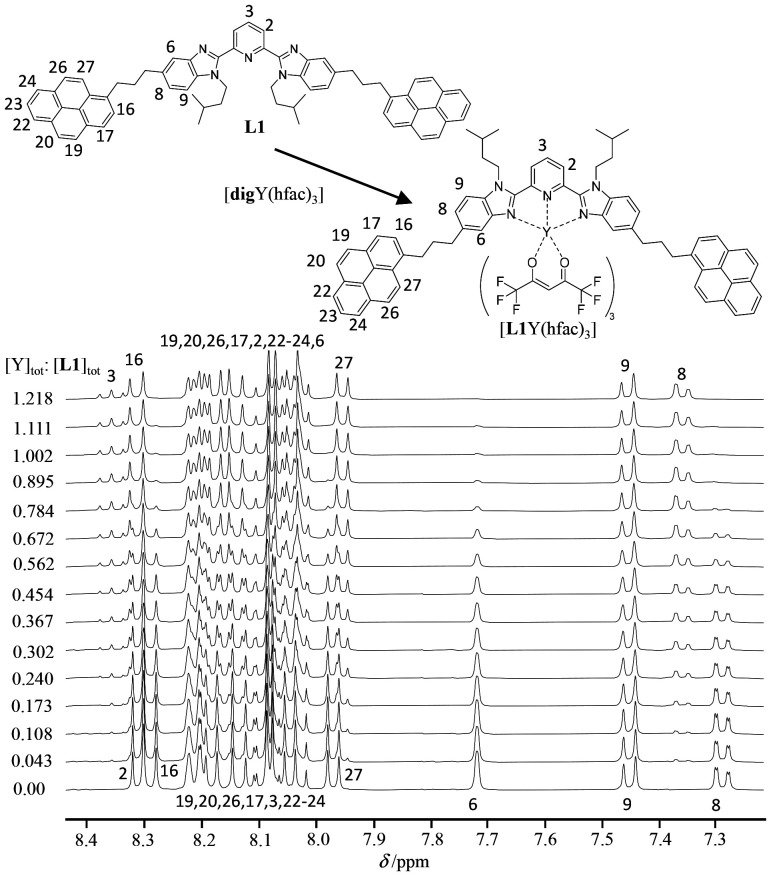
^1^H NMR titration of L1 with [digY(hfac)_3_] at 293 K in CD_2_Cl_2_ + 0.146 M dig.

**Fig. 7:**
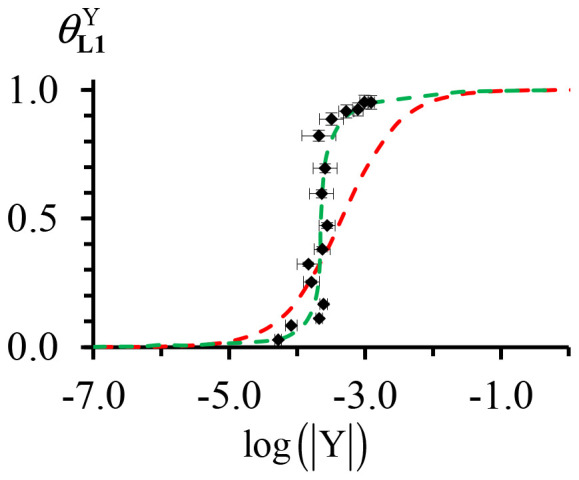
Experimental (black diamonds) and fitted binding isotherms using eqn (4) (dashed-red trace) and eqn (A4-3) (dashed-green trace) for the titration of L1 with [digY(hfac)_3_].

Practically, at each point of the titration, the ^1^H NMR spectrum (Fig. 6) provides reliable integrations for a given proton, for instance H^8^ and H^8bound^, corresponding to the free (*I*_**L1**_) and the bound (*I*_**L1**Y_) ligand L1. The well-known occupancy factor *θ*^Y^_**L1**_ = *I*_**L1**Y_/(*I*_**L1**Y_ + *I*_**L1**_) and the free concentration |Y| = |Y|_tot_ − *θ*^Y^_**L1**_|L1|_tot_ of the trivalent yttrium in solution can be then calculated using eqn (4) for building the experimental binding isotherm (black diamonds in Fig. 7).4
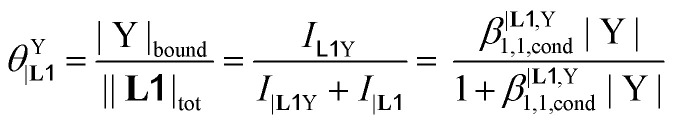


The conditional (CD_2_Cl_2_ + 0.146 M diglyme) stability constant *β*^**L1**,Y^_1,1,cond_ = 2200(361) can be estimated from the nonlinear least-squares fit of *θ*^Y^_**L1**_*versus* |Y| with the help of eqn (4). The binding isotherm rebuilt from the obtained stability constant (dashed-red traces in Fig. 7) differs from the experimental curve (black diamonds in Fig. 7) due to the variation in the activity coefficients occurring in non-ideal organic solutions.^27^ An extended analysis, which specifically models infinite dilution conditions, is given in Appendix 5 SI and provides a satisfactory binding isotherm (green trace in Fig. 7). The titration process reveals non-uniform chemical shift changes for aromatic protons of the terimine unit in the bound ligand L1 due to the electronic effects induced by the Y(iii)–N interactions upon binding to the lanthanide cargo (Fig. 6). For instance: (i) the *meta*-positioned proton H^9^ displays no chemical shift change, while (ii) the *para*-positioned proton H^8^ shows a downfield shift due to the resonance π-delocalization and (iii) the proton H^6^ exhibits a significant downfield shift due to the combination of both resonance effect originating from the nitrogen atom and inductive effect from the electron-withdrawing trivalent yttrium center.

From the calculated exchange binding *β*^**L1**,Y^_1,1,exch_ = *β*^**L1**,Y^_1,1,cond_|dig|_tot_ = 321(53), a rough, but pertinent association constant of *β*^**L1**,Y^_1,1_ ≅ 3 × 10^5^ can be estimated in the absence of competitive diglyme by fixing |dig|_tot_ ≤ 10^−3^ M. This ensures the quantitative formation (≥94%) of the monomeric [L1Y(hfac)_3_] complex at the concentration of 10^−4^ M used for the photophysical studies. Moreover, Table 1 confirms that negligible traces of the dimer are expected to occur in the ground state (static excimer) under these dilute conditions.

### Excited state aggregation

**Fig. 8:**
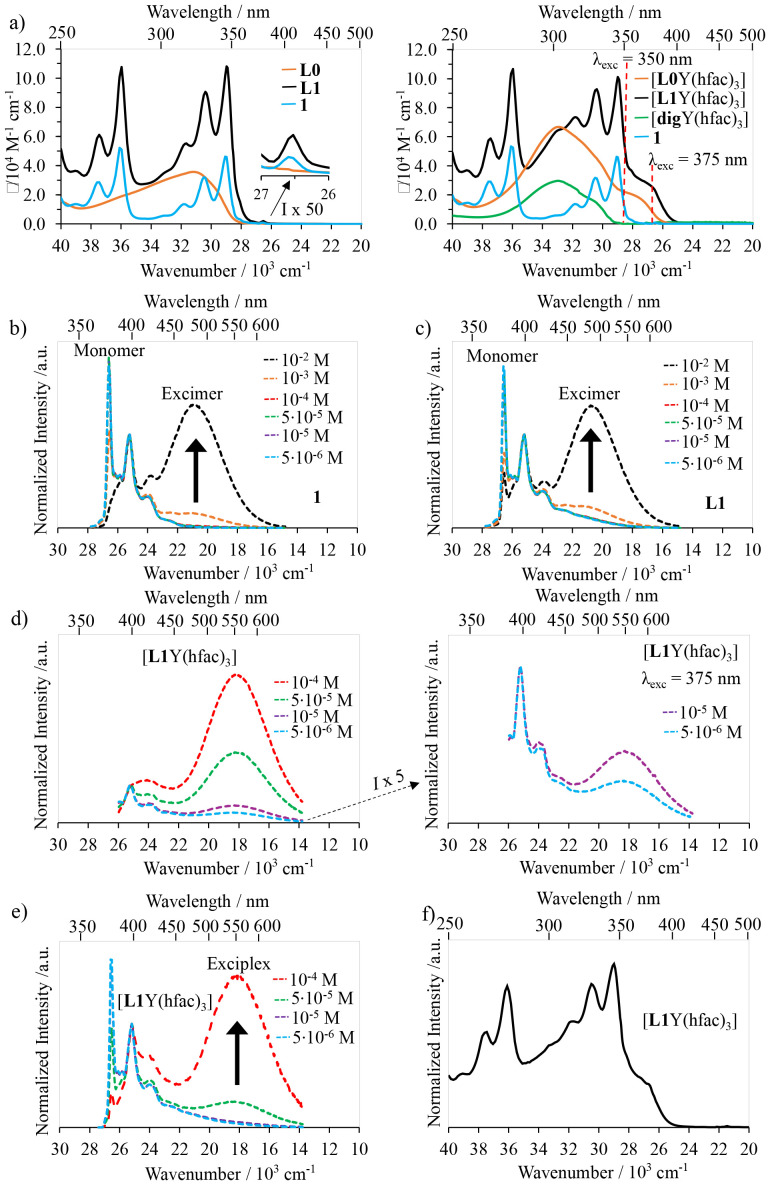
(a) Absorption spectra of 1, L0, L1, [L0Y(hfac)_3_], [L1Y(hfac)_3_], and [digY(hfac)_3_]. Emission spectra of (b) 1 (*λ*_exc_ = 345 nm), (c) L1 (*λ*_exc_ = 345 nm), (d) [L1Y(hfac)_3_] (*λ*_exc_ = 375 nm), and (e) [L1Y(hfac)_3_] (*λ*_exc_ = 350 nm), and (f) excitation spectrum of [L1Y(hfac)_3_] (*λ*_analysis_ = 548 nm).

The UV-Vis absorption spectra of pyrene-bearing 1, terimine ligand L0, pyrene–terimine ligand L1 and their yttrium complexes were recorded in CH_2_Cl_2_ solution at room temperature and are shown in Fig. 8a. The absorption band associated with the terimine unit in L1 is masked by the stronger absorption of the pyrene chromophore which displays the spin-allowed ^1^π_3_* ← ^1^π (256–280 nm), ^1^π_2_* ← ^1^π (303–356 nm) and ^1^π_1_* ← ^1^π (370 nm) transitions, reminiscent of those observed in compound 1 (Fig. 8a, left). The terimine unit undergoes a *trans*–*trans* → *cis*–*cis* conformational change upon complexation of either L0 or L1 to the metal to give [L*k*Y(hfac)_3_], which is responsible for the appearance of additional well-documented weaker absorption ^1^π_2a_* ← ^1^π bands at lower energies (350–400 nm, Fig. 8a, right).^28,29^ As a result, irradiation at 375 nm can be exploited to selectively excite the bound terimine unit of ligand L1 in the [L1Y(hfac)_3_] complex, with negligible contributions of the concomitant hfac^−^ and pyrene moieties to the light conversion process.

Let's start by focusing on the excitation at *λ*_exc_ = 345–350 nm where both the pyrene and the aromatic terimine units are excited into their ^1^π* levels. The resulting emission spectrum recorded for the pure pyrene compound 1 under dilute conditions (10^−4^ to 5 × 10^−6^ M) displays the 370–390 nm emission band diagnostic for monomeric pyrene (blue trace in Fig. 8b). At higher concentrations (10^−3^ to 10^−2^ M), an additional broad pyrene–pyrene excimer emission around 477 nm completes the emission spectrum (black trace in Fig. 8b). In strict analogy, the emission spectra of the free ligand L1 under 345 nm excitation display the pyrene monomer emission at low concentrations (blue trace in Fig. 8c) and the pyrene–pyrene excimer emission at high concentrations (black trace in Fig. 8c), thus demonstrating no communication between the pyrene and the terimine units for L1 in its excited state, a feature mimicking its ground state behaviour (solid state and solution, *vide supra*). In contrast, the same global excitation at 350 nm of the alternative bound ligand L1 in [L1Y(hfac)_3_] leads to an unprecedented broad emission at 548 nm assigned to the exciplex emission generated by the intermolecular π-interaction between the pyrene and the terimine units in the excited state (Fig. 8e and A6-1a SI, left), as previously identified in the ground state (Fig. 2 and 4, *vide supra*). Selective excitation at 375 nm into the aromatic terimine unit in [L1Y(hfac)_3_] confirms the exciplex emission occurring at 548 nm, even at very low concentrations of 10^−5^–10^−6^ M (Fig. 8d and A6-1a SI, right) in agreement with strong communication between the excited state of the terimine unit donor and the ground state pyrene acceptor under micromolar conditions. In Fig. 8d (right), at dilute concentrations (10^−5^ to 5 × 10^−6^ M), the emission profile around 400 nm is dominated by the typical monomer emission of the pyrene unit, which fully masks the singlet excited ^1^π_1_* → ^1^π transition of the terimine unit in the [L1Y(hfac)_3_] complex. In contrast, at higher concentrations (10^−2^ to 5 × 10^−5^ M), the emission band in the 400–450 nm range is mainly governed by the terimine-based ^1^π_1_* → ^1^π transition (Fig. 8d and A6-1a SI). This attribution is supported by the comparable ligand-based emission profile of the free pyrene [L0Y(hfac)_3_] model shown in Fig. A6-1d SI (Appendix A6 SI). Under concentrated conditions, the pyrene monomer emission is significantly quenched and efficiently converted into the intermolecular exciplex emission at 548 nm (Fig. 8d and A6-1a SI). The excitation spectra monitored at *λ*_analysis_ = 548 nm of [L1Y(hfac)_3_] (Fig. 8f) strictly mirror its absorption spectrum (Fig. 8a, right), confirming that the dimerization reaction in the excited state involves both the pyrene and the terimine units.

**Fig. 9:**
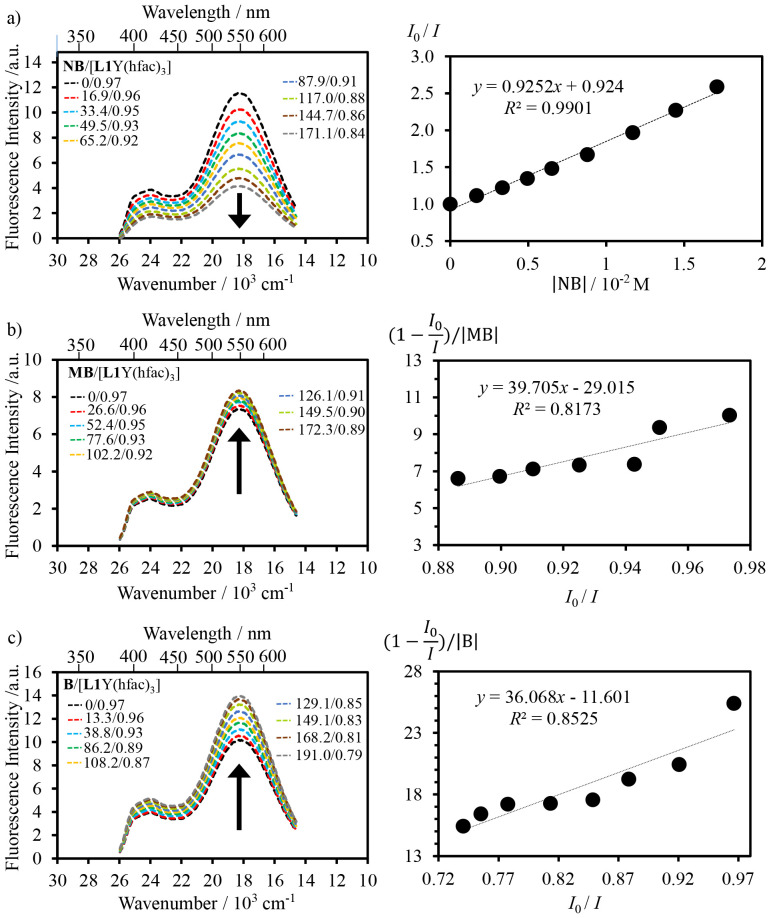
Fluorescence titrations of [L1Y(hfac)_3_] (*λ*_exc_ = 375 nm) with (a) NB, (b) MB and (c) B in dichloromethane (293 K) and associated Stern–Volmer analysis using eqn (5) and (6) (see the text). The molar ratios are expressed in 10^−4^ M units.

To further elucidate the nature and electronic sensitivity of the exciplex, the interaction between the [L1Y(hfac)_3_] host and the aromatic compound guests (MB, B, NB) has been investigated in the excited state at a low 10^−4^ M concentration to avoid the contribution of the static dimerization to the light-driven dynamic excimer formation (Table 1). Under excitation at 375 nm and 350 nm, the exciplex emission intensity decreases upon addition of the electron-poor NB quencher to the solution of [L1Y(hfac)_3_] due to competitive π-stacking interactions between the NB guest, the terimine and the pyrene units in the host structure (Fig. 9a). The fluorescence quenching efficiency by NB is determined with the help of Stern–Volmer eqn (5),^30,31^ in which *I*_0_ and *I* refer, respectively, to the excimer emission intensities in the absence and presence of the quencher, |*Q*| is the concentration of the quencher, and *K*_SV_ is the Stern–Volmer quenching constant:5
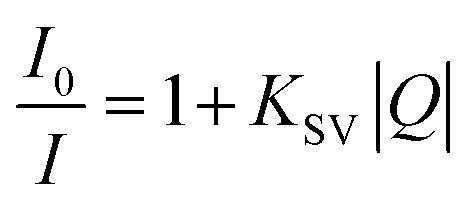


**Fig. 10:**
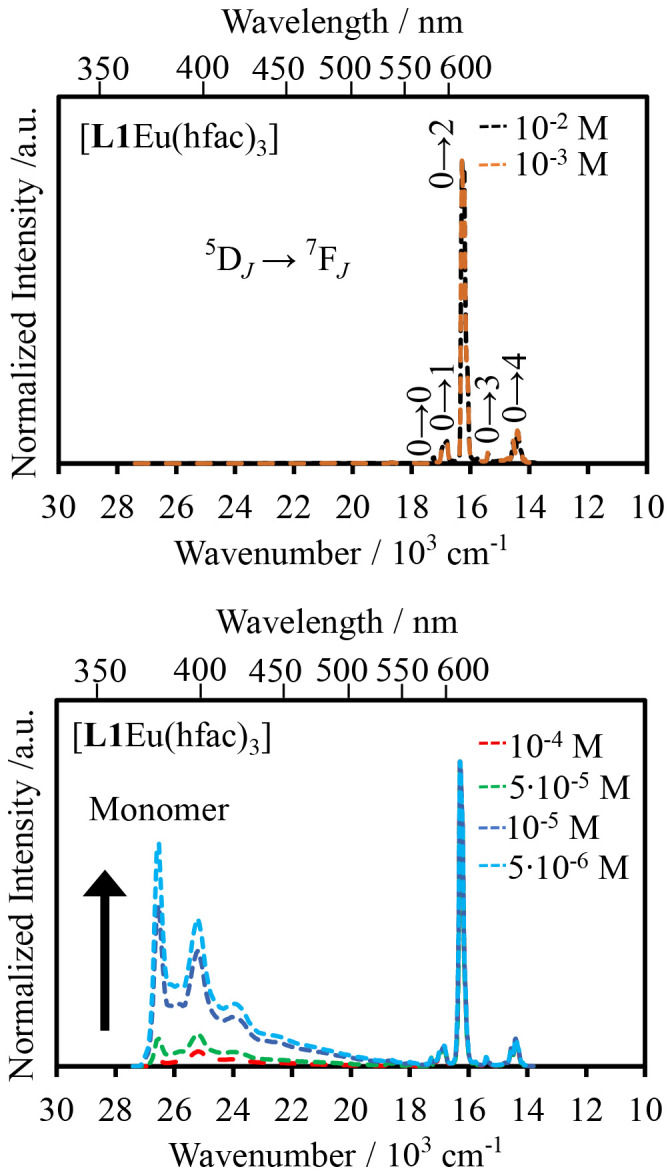
Emission spectra of [L1Eu(hfac)_3_] (*λ*_exc_ = 350 nm) at decreasing concentrations (top to bottom).

The fluorescence titration of [L1Y(hfac)_3_] with the NB quencher displays a linear Stern–Volmer plot (Fig. 9a, right), from which *K*_SV_ = 93 M^−1^ can be determined. In agreement with the ground-state dimer dissociation observed by ^1^H NMR titration of [L1Y(hfac)_3_] with NB (Fig. 5a), the addition of electron-poor NB also destroys the excited-state dimeric organization of [L1Y(hfac)_3_] at both 10^−4^ M (Fig. 9a) and 10^−2^ M (Fig. A6-2 SI). Interestingly, upon fluorescence titration of [L1Y(hfac)_3_] at 10^−4^ M with the electron-rich MB and the neutral B, the exciplex emission is slightly enhanced (Fig. 9b and c). This behaviour could be explained by the formation of a complex between the excited-state dimeric organization and the supplementary aromatic guest,^32,33^ again mimicking the ground state π-stacking interactions operating between the pyrene unit and the aromatic compound (B and MB) observed upon ^1^H NMR titration (Fig. 5b and c). This enhancement effect is quantified by the equilibrium constants *K*_B_ = 11 M^−1^ and *K*_MB_ = 29 M^−1^ of the exciplex–guest binding reactions determined as the slope of the plot of (1 − *I*_0_/*I*)/|Guest| *versus I*_0_/*I* according to eqn (6) (Fig. 9b and c, right).^32^6
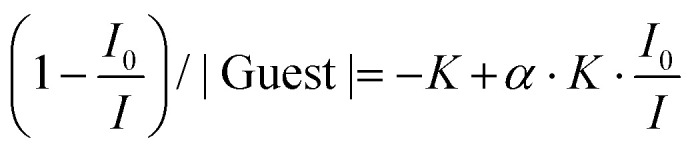
*I*_0_ and *I* refer, respectively, to the excimer emission intensities in the absence and presence of the guest, |Guest| is the concentration of the guest (B or MB), *α* represents the fluorescence quantum efficiency of the exciplex–aromatic complex, and *K* is the association equilibrium constant.

When Y^3+^ is replaced with Eu^3+^ in the [L1Eu(hfac)_3_] complex, excitation at 350 nm into the bound aromatic ligand L1 displays concentration-dependent emission spectra (Fig. 10). At dilute concentrations (10^−6^–10^−4^ M), the emission spectra recorded for [L1Eu(hfac)_3_] display both pyrene monomer ^1^π_1_* → ^1^π (370–450 nm) and europium-centered Eu(^5^D_0_→^7^F_*J*_, *J* = 0–4; 580–700 nm) emission bands (Fig. 10, bottom). Upon increasing the concentration (10^−2^–10^−3^ M), only the characteristic Eu-centered f–f transitions ^5^D_0_ → ^7^F_0_ (579 nm), ^5^D_0_ → ^7^F_1_ (594 nm), ^5^D_0_ → ^7^F_2_ (615 nm), ^5^D_0_ → ^7^F_3_ (652 nm), and ^5^D_0_ → ^7^F_4_ (703 nm) are detected (Fig. 10, top).^34^ The lack of exciplex emission at any concentration of [L1Eu(hfac)_3_] indicates that an efficient energy transfer process connects the exciplex level (donor) onto the Eu(iii) activator. The contribution of the exciplex working as an antenna for the Eu-centered luminescence is eventually confirmed by the observation of the characteristic bands of the pyrene unit in the excitation spectrum of [L1Eu(hfac)_3_] monitored at 614 nm (Fig. A6-1e SI). To the best of our knowledge, this is the first report of the exciplex-to-europium energy transfer in an aggregation-induced organization.

## Conclusions

This work provides a molecular approach for tailoring the π–π interactions in organic materials *via* a lanthanide-based tuning of their supramolecular organizations and their light emission properties. In the ground state, the faint (negligible) homo-π-stacking interactions involving the terminal neutral pyrene units in ligand L1 give rise to unprecedented hetero-π-stacking interactions between the neutral pyrene ring and the electron-poor terimine unit bound to the trivalent metal in the [L1Ln(hfac)_3_] (Ln = Y, Eu) complexes. Upon light excitation, the free ligand L1 displays a standard homo pyrene–pyrene excimer emission at high concentrations (10^−2^–10^−3^ M). Again, because of lanthanide complexation, the corresponding [L1Y(hfac)_3_] adduct exhibits the alternative hetero pyrene–terimine exciplex emission at close to micromolar concentrations (10^−5^–10^−6^ M). Complexation to the Eu(iii) activator in [L1Eu(hfac)_3_] conveys the energy of the ligand-based excited states onto the Eu(^5^D_0_) emissive level, except at a very low concentration where ‘free’ terminal pyrene groups restore monomeric ring-centered emission. In the presence of competitive aromatic guests (nitrobenzene, benzene, methoxybenzene), only the electron-poor nitrobenzene strongly interacts *via* π-stacking interactions with the pyrene residues and induces dissociation of [L1Y(hfac)_3_]_2_ both in its ground and excited states.

## Author contributions

G. L.-H. designed the synthetic strategies, performed all the experiments and wrote the first draft of the contribution. L. G. solved the crystal structures. C. P. managed the whole project, obtained funding and finalized the version to be published.

## Conflicts of interest

There are no conflicts to declare.

## Supplementary Material

DT-055-D6DT00490C-s001

DT-055-D6DT00490C-s002

## Data Availability

Supplementary information (SI): experimental section, NMR spectra, crystal structures, thermodynamic data, and photophysical data for the synthesized compounds. See DOI: https://doi.org/10.1039/d6dt00490c. CCDC 2531606–2531611 contain the supplementary crystallographic data for this paper.^35*a*–*f*^
