# Supplementary material for: Tailoring exciplex formation in metal-induced supramolecular organization
Source: Dalton Trans. 2026 Apr 6;55(22):8481–8. doi: 10.1039/d6dt00490c (PMC13093875; doi:10.1039/d6dt00490c)
Supplement: DT-055-D6DT00490C-s001 [file DT-055-D6DT00490C-s001.pdf]

Supplementary Information (SI) for Dalton Transactions.
This journal is © The Royal Society of Chemistry 2026

Tailoring Exciplex Formation in Metal-Induced Supramolecular Organization

Giau Le-Hoang,*[a] Laure Guénée,[b] and Claude Piguet*[a]

[a] *Department of Inorganic and Analytical Chemistry, University of Geneva, 30 quai E. Ansermet, CH-1211 Geneva 4, Switzerland.*

Emails: Hoang.Le@unige.ch, Claude.Piguet@unige.ch

[b] *Laboratory of Crystallography, Univ ersity of Geneva, 24 quai E. Ansermet, CH-1211 Geneva 4, Switzerland.*

Supporting Information

(62 pages)

Appendix 1: Experimental Section

General. All reagents were purchased from Alfa Aesar, FluroChem, Acros, and Sigma-Aldrich, and used as received. Compounds **L0**,[A1-1] [**L0**Y(hfac)$_3$],[A1-1] [**L0**Eu(hfac)$_3$],[A1-1] **1**,[A1-2] **2**,[A1-3] **4**,[A1-4] [**dig**Eu(hfac)$_3$],[A1-5] and [**dig**Y(hfac)$_3$][A1-6] were prepared according to literature. Dichloromethane was distilled over calcium hydride.

Spectroscopic and analytical measurements

^{1}H and ^{13}C NMR spectra were recorded at 298 K on a Bruker Avance 400 MHz spectrometer equipped with BCU temperature control for variable temperature measurements. Electrospray (ESI-MS) mass spectra were recorded on an Applied Biosystems API 150EX LC/MS System equipped with a Turbo Ionspray source. Elemental analyses were performed by K. L. Paglia from the Microchemical Laboratory of the University of Geneva. Electronic spectra in the UV-Vis region were recorded at 293 K from solutions in CH_2Cl_2 with a Perkin-Elmer Lambda 1050. The emission spectra were recorded using a Fluorolog (Horiba Jobin-Yvon) instrument equipped with an iHR320 imaging spectrometer, a 450 W xenon lamp illuminator (FL-1039A/40A) and a Peltier-cooled photomultiplier tube (PMT Hamamatsu R928P). The emission spectra were corrected for the wavelength-dependent sensitivity of the PMT. The standard xenon lamp of the Horiba Scientific Fluorolog 3 spectrofluorometer has been used for UV excitation of the samples to record the pertinent emission spectra, where the appropriate longpass filters (Thorlabs) have been placed after the sample to remove the second-order Rayleigh scattering of the xenon lamp. The emission spectra were corrected for the instrumental response function. The mathematical analyses were performed by using Origin 2017 (OriginLab Corporation) and Excel® (Microsoft) software.

Synthesis of target ligands L1 and L2.

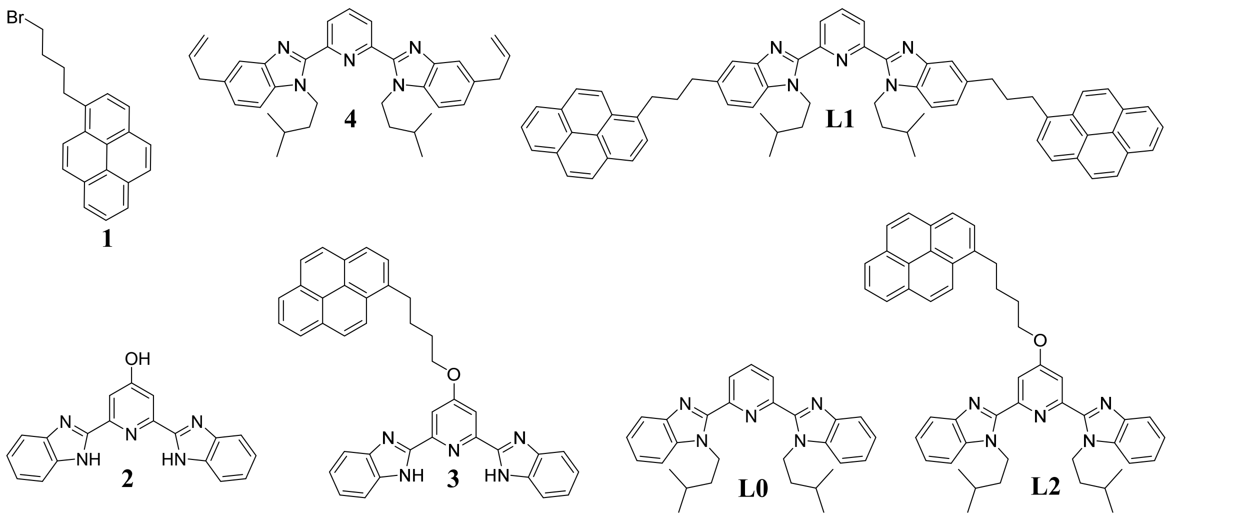

Figure A1-1. Chemical structures of synthesized compounds in this work.

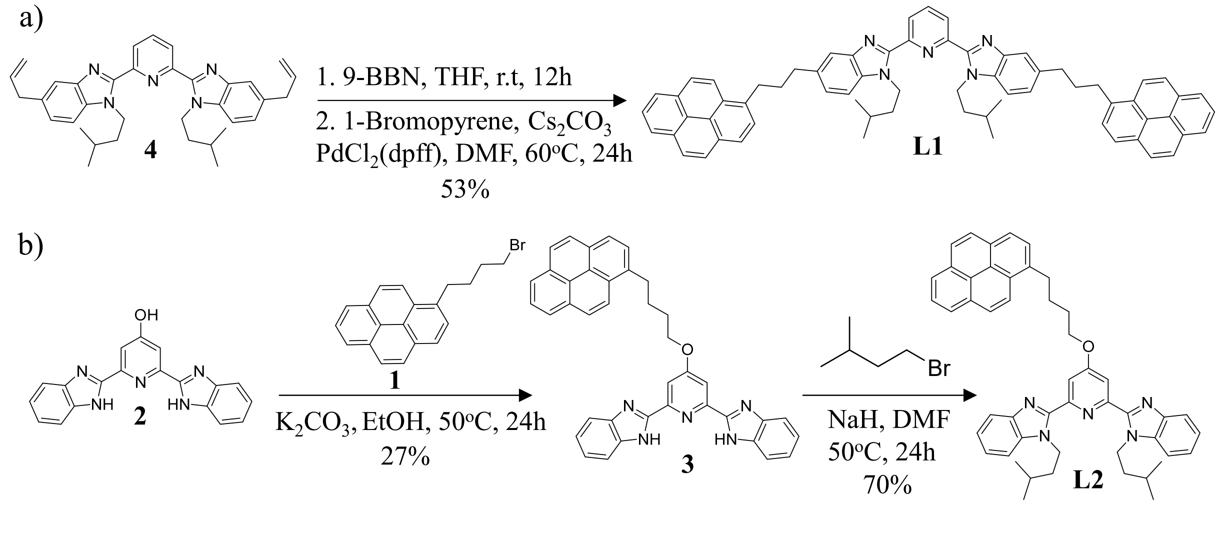

Scheme A1-1. Synthesis of the tridentate ligands **L1** and **L2**.

Preparation of L1. To a solution of **4** (2.5 g, 4.7 mmol) in dry THF (25 mL), 9-borabicyclo[3.3.1]nonane (9-BBN, 0.5 M, 48 mL, 24 mmol) was added at 0°C. The solution was stirred at r.t for 12h. 1-Bromopyrene (13 g, 46.2 mmol), Cs_2CO_3 (7.8 g, 24 mmol), Pd_2Cl_2(dppf) (0.7 g, 0.96 mmol; dppf = (ferrocene-1,1′-diyl)bis(diphenylphosphane)), and DMF (25 mL) were

added. The mixture was stirred at 60°C for 24h and then cooled to room temperature. H_4EDTA (15 g, 51.3 mmol) and H_2O (30 mL) were added. The mixture was stirred at r.t for 1h. The mixture was extracted with CH_2Cl_2 (3 x 100 mL), dried over Na_2SO_4 and evaporated to dryness. The residue was purified by column chromatography (SiO_2, CH_2Cl_2/MeOH 100/0 to 100/10) to afford **L1** (2.35 g, 53%) as a white solid. ^1H NMR (400 MHz, CD_2Cl_2): 8.31 (d, J = 6.4 Hz, 2H), 8.29 (d, J = 7.7 Hz, 2H), 8.25 – 8.18 (m, 4H), 8.18 (d, J = 7.8 Hz, 2H), 8.14 (d, J = 9.3 Hz, 2H), 8.11 – 8.02 (m, 7H), 7.97 (d, J = 7.8 Hz, 2H), 7.72 (d, J = 0.9 Hz, 2H), 7.48 – 7.43 (m, 2H), 7.29 (dd, J = 8.4, 1.6 Hz, 2H), 4.82 – 4.73 (m, 4H), 3.52 – 3.43 (m, 4H), 3.02 (t, J = 7.6 Hz, 4H), 2.39 – 2.27 (m, 4H), 1.71 – 1.62 (m, 4H), 1.51 – 1.41 (m, 2H), 0.73 (d, J = 6.6 Hz, 12H). ^{13}C NMR (101 MHz, CD_2Cl_2): 150.2, 143.3, 137.9, 137.1, 136.7, 134.8, 131.4, 130.9, 129.7, 128.6, 127.5, 127.3, 127.0, 126.4, 125.8, 125.1, 125.0, 124.9, 124.8, 124.7(6), 124.6, 124.3, 123.5, 119.4, 110.0, 43.5, 38.8, 36.0, 34.0, 33.0, 25.8, 21.9. ESI-MS calculated for $[C_{67}H_{61}N_5 + H]^+$ ($[\mathbf{L1} + H]^+$): m/z 936.5; found: 937.1. Elemental analysis calculated for $C_{67}H_{61}N_5$ (**L1**) (%): C 85.95, H 6.57, N 7.48; found (%): C 85.31, H 6.49, N 7.35.

Preparation of 3. A mixture of **2** (1.0 g, 3.05 mmol) and K_2CO_3 (0.42 g, 3.04 mmol) in EtOH (50 mL) was stirred at 80°C for 1h. **1** (1.55 g, 4.60 mmol) and KI (catalytic amount) were added. The solution was heated at 80°C for 24h and then evaporated to dryness. H_2O (100 mL) was added. The resulting solid was filtered off and purified by column chromatography (SiO_2, CH_2Cl_2/MeOH 100/0 to 100/5) to afford **2** (0.48 g, 27%) as a white solid. ^1H NMR (400 MHz, DMSO-*d6*): 12.94 (s, 2H), 8.42 (d, J = 9.3 Hz, 1H), 8.29 – 8.19 (m, 4H), 8.15 – 7.99 (m, 4H), 7.83 (s, 2H), 7.78 (d, J = 8.0 Hz, 2H), 7.73 (d, J = 7.9 Hz, 2H), 7.39 – 7.33 (m, 2H), 7.32 – 7.25 (m, 2H), 4.40 (d, J = 6.1 Hz, 2H), 3.45 (t, J = 7.1 Hz, 2H), 2.08 – 1.91 (m, 4H). ^{13}C NMR (101 MHz, DMSO-*d6*): 169.9, 153.7, 152.7, 147.4, 140.0, 137.6, 134.2, 133.7, 132.6, 131.4, 130.8, 130.7, 130.5, 129.8, 129.4,

128.3, 128.2, 128.0, 127.6, 127.5, 127.0, 126.8, 125.5, 123.0, 115.1, 110.7, 71.6, 58.2, 35.4, 31.6, 30.9.

Preparation of L2. A mixture of **2** (0.34 g, 0.63 mmol) and 60% NaH (0.08 g, 2 mmol) in dry DMF (5 mL) was stirred at r.t for 30 min. 1-Bromo-3-methylbutane (1.8 g, 1.19 mmol) and KI (catalytic amount) were added. The solution was heated at 50 °C for 24h and then quenched with water (10 mL). The mixture was extracted with CH_2Cl_2 (3 x 20 mL), dried over Na_2SO_4 and evaporated to dryness. The residue was purified by column chromatography (SiO_2, CH_2Cl_2/MeOH 100/0 to 100/10) to afford **L2** (0.3 g, 70%) as a white solid. ^{1}H NMR (400 MHz, CD_2Cl_2): 8.39 (d, *J* = 9.2 Hz, 1H), 8.24 – 8.18 (m, 4H), 8.11 – 8.02 (m, 3H), 8.00 (d, *J* = 7.8 Hz, 1H), 7.87 (s, 2H), 7.84 – 7.80 (m, 2H), 7.53 – 7.49 (m, 2H), 7.41 – 7.31 (m, 4H), 4.82 – 4.74 (m, 4H), 4.38 (t, *J* = 5.8 Hz, 2H), 3.52 (t, *J* = 7.3 Hz, 2H), 2.20 – 2.09 (m, 4H), 1.68 – 1.61 (m, 4H), 1.49 – 1.40 (m, 2H), 0.73 (d, *J* = 6.6 Hz, 12H). ^{13}C NMR (101 MHz, CD_2Cl_2): 166.5, 151.7, 150.3, 142.9, 136.6, 136.4, 131.4, 130.9, 129.8, 128.6, 127.5, 127.3, 127.2, 126.5, 125.8, 125.0, 124.9, 124.8(4), 124.8(2), 124.7, 123.4, 123.1, 122.3, 120.0, 111.8, 110.3, 68.6, 43.4, 38.7, 33.0, 28.9, 28.1, 25.8, 21.9. ESI-MS calculated for $[C_{49}H_{49}N_5O + H]^+$ ($[\mathbf{L2} + H]^+$): *m/z* 724.4; found: 724.9. Elemental analysis calculated for $C_{49}H_{49}N_5O$ (**L2**) (%): C 81.29, H 6.82, N 9.67; found (%): C 81.25, H 7.02, N 9.71.

General procedure for synthesis of [L*k*Ln(hfac)$_3$] (L*k* = L1, L2 and Ln = Eu, Y) complexes

A mixture of ligand **L*k*** and [**dig**Ln(hfac)$_3$] (1.1 eq.) in CH_2Cl_2 was stirred at r.t for 30 min and then evaporated to dryness. The residue was purified by precipitation in pentane to afford [**L*k***Ln(hfac)$_3$].

[L1Eu(hfac)$_3$]. Yield: 93%. ^{1}H NMR (400 MHz, CD_2Cl_2): 21.32 (br, 2H), 9.64 (d, *J* = 8.5 Hz, 2H), 9.07 (d, *J* = 9.3 Hz, 2H), 8.59 (dd, *J* = 16.3, 8.2 Hz, 4H), 8.42 (d, *J* = 7.8 Hz, 2H), 8.33 (d, *J* = 9.2 Hz, 2H), 8.27 – 8.22 (m, 4H), 8.20 (d, *J* = 8.9 Hz, 2H), 8.13 (d, *J* = 8.9 Hz, 2H), 8.05 (t, *J* = 7.6 Hz, 2H), 7.51 (t, *J* = 7.8 Hz, 1H), 6.83 (d, *J* = 7.9 Hz, 2H), 5.93 – 5.79 (m, 4H), 5.68 – 5.49 (m,

4H), 4.71 – 4.58 (m, 4H), 4.09 – 3.95 (m, 4H), 3.07 – 2.94 (m, 4H), 2.47 – 2.35 (m, 2H), 1.40 (d, *J* = 6.6 Hz, 12H). ^{13}C NMR (101 MHz, CD_2Cl_2): 154.7, 154.0, 146.2, 145.8, 144.2, 137.9, 131.5, 131.2, 130.0, 129.2, 128.9, 127.9, 127.6, 127.2, 126.5, 125.8, 125.3, 125.1, 124.7, 124.6, 124.1, 115.4, 110.9, 99.7, 60.2, 56.1, 44.4, 40.9, 38.9, 36.2, 34.6, 26.8, 22.4. ESI-MS calculated for [$C_{82}H_{64}N_5O_6F_{18}$Eu - hfac]$^+$ ([[**L1**Eu(hfac)$_3$] - hfac]$^+$): *m/z* 1502.4; found: 1502.0. Elemental analysis calculated for $C_{82}H_{64}N_5O_6F_{18}$Eu ([**L1**Eu(hfac)$_3$]) (%): C 57.62, H 3.77, N 4.10; found (%): C 57.42, H 3.77, N 4.12.

[L1Y(hfac)$_3$]. Yield: 84%. ^{1}H NMR (400 MHz, CD_2Cl_2): 8.36 (d, *J* = 8.0 Hz, 1H), 8.32 (d, *J* = 9.1 Hz, 2H), 8.21 (dd, *J* = 7.6, 4.2 Hz, 4H), 8.18 (d, *J* = 7.8 Hz, 2H), 8.14 (d, *J* = 9.2 Hz, 2H), 8.11 – 8.01 (m, 10H), 7.96 (d, *J* = 7.8 Hz, 2H), 7.45 (d, *J* = 8.5 Hz, 2H), 7.36 (dd, *J* = 8.6, 1.5 Hz, 2H), 5.92 (s, 3H), 4.62 – 4.52 (m, 4H), 3.49 – 3.41 (m, 4H), 2.98 (t, *J* = 7.9 Hz, 4H), 2.28 – 2.18 (m, 4H), 2.03 – 1.94 (m, 4H), 1.94 – 1.86 (m, 2H), 1.13 (d, *J* = 6.4 Hz, 12H). ^{13}C NMR (101 MHz, CD_2Cl_2): 175.8, 175.5, 175.2, 174.8, 148.7, 147.5, 140.6, 140.2, 138.8, 137.1, 134.3, 131.4, 131.0, 129.7, 128.6, 127.5, 127.3, 127.0, 126.4, 126.1, 125.7, 125.0, 124.9, 124.8, 124.7, 124.6, 123.5, 122.6, 122.1, 121.4, 119.2, 116.3, 113.5, 109.6, 89.6, 44.7, 38.3, 36.2, 34.0, 33.4, 26.4, 22.1. ESI-MS calculated for [$C_{82}H_{64}F_{18}N_5O_6$Y - hfac]$^+$ ([[**L1**Y(hfac)$_3$] - hfac]$^+$): *m/z* 1438.4; found: 1438.6. Elemental analysis calculated for $C_{82}H_{64}F_{18}N_5O_6Y\cdot0.4CH_2Cl_2$ ([**L1**Y(hfac)$_3$]·0.4CH_2Cl_2) (%): C 58.90, H 3.89, N 4.17; found (%): C 58.75, H 3.63, N 4.19.

[L2Eu(hfac)$_3$]. Yield: 85%. ^{1}H NMR (400 MHz, CD_2Cl_2): 22.08 (br, 2H), 11.16 (br, 2H), 10.03 – 9.95 (m, 2H), 8.85 (dd, *J* = 8.4, 0.9 Hz, 2H), 8.17 (dd, *J* = 7.2, 1.5 Hz, 1H), 8.11 (d, *J* = 9.2 Hz, 1H), 8.08 – 8.00 (m, 2H), 7.93 (d, *J* = 9.2 Hz, 1H), 7.82 (d, *J* = 8.9 Hz, 1H), 7.74 – 7.61 (m, 2H), 7.41 (d, *J* = 8.9 Hz, 1H), 5.63 (s, 2H), 5.56 – 5.44 (m, 4H), 3.92 (t, *J* = 6.1 Hz, 2H), 3.29 (t, *J* = 7.2 Hz, 2H), 2.87 – 2.78 (m, 4H), 2.65 (s, 3H), 2.33 – 2.18 (m, 2H), 1.96 – 1.77 (m, 4H), 1.27 (d, *J* =

6.6 Hz, 12H). ^{13}C NMR (101 MHz, CD_2Cl_2): 173.4, 159.1, 156.5, 151.7, 144.0, 135.9, 132.3, 131.3, 131.2, 130.7, 129.6, 128.4, 128.3, 127.2, 127.0(2), 127.0, 126.5, 126.0, 125.1, 124.8, 124.7, 124.4, 122.9, 118.4, 111.4, 81.9, 68.7, 62.3, 60.4, 59.4, 56.6, 44.1, 40.6, 32.5, 28.3, 27.1, 26.9, 22.3. ESI-MS calculated for [$C_{64}H_{52}F_{18}N_6O_7Eu$ - hfac]$^+$ ([[**L2**Eu(hfac)$_3$] - hfac]$^+$): *m/z* 1290.3; found: 1290.1. Elemental analysis calculated for $C_{64}H_{52}F_{18}N_6O_7Eu$ ([**L2**Eu(hfac)$_3$]) (%): C 51.35, H 3.50, N 4.68; found (%): C 51.39, H 3.58, N 4.70.

[L2Y(hfac)$_3$]. Yield: 78%. ^{1}H NMR (400 MHz, CD_2Cl_2): 8.35 (d, J = 9.3 Hz, 1H), 8.22 – 7.86 (m, 10H), 7.53 – 7.44 (m, 4H), 7.43 – 7.34 (m, 2H), 7.32 (s, 2H), 5.93 (s, 3H), 4.46 – 4.35 (m, 4H), 4.24 (t, J = 5.7 Hz, 2H), 3.52 (t, J = 6.8 Hz, 2H), 2.18 (qq, J = 7.3, 3.8 Hz, 4H), 1.83 (ddq, J = 24.4, 13.3, 6.5 Hz, 6H), 1.06 (d, J = 6.1 Hz, 12H). ^{13}C NMR (101 MHz, CD_2Cl_2): 175.8, 175.5, 175.1, 174.8, 167.7, 148.9, 148.8, 148.7, 140.1, 136.1, 135.8, 131.3, 130.8, 129.9, 128.6, 127.4, 127.3, 127.2, 126.6, 126.0, 125.1, 125.0, 124.9, 124.8, 124.7(5), 124.7(3), 123.7, 123.1, 122.3, 122.0, 119.2, 116.3, 113.5, 109.7, 109.3, 89.5, 69.2, 44.5, 38.2, 32.7, 28.6, 27.4, 26.5, 22.1. ESI-MS calculated for [$C_{64}H_{52}N_5O_7F_{18}Y$ - hfac]$^+$ ([[**L2**Y(hfac)$_3$] - hfac]$^+$): *m/z* 1226.3; found: 1227.0. Elemental analysis calculated for $C_{64}H_{52}N_5O_7F_{18}Y$ ([**L2**Y(hfac)$_3$]) (%): C 53.60, H 3.66, N 4.88; found (%): C 53.59, H 3.80, N 4.92.

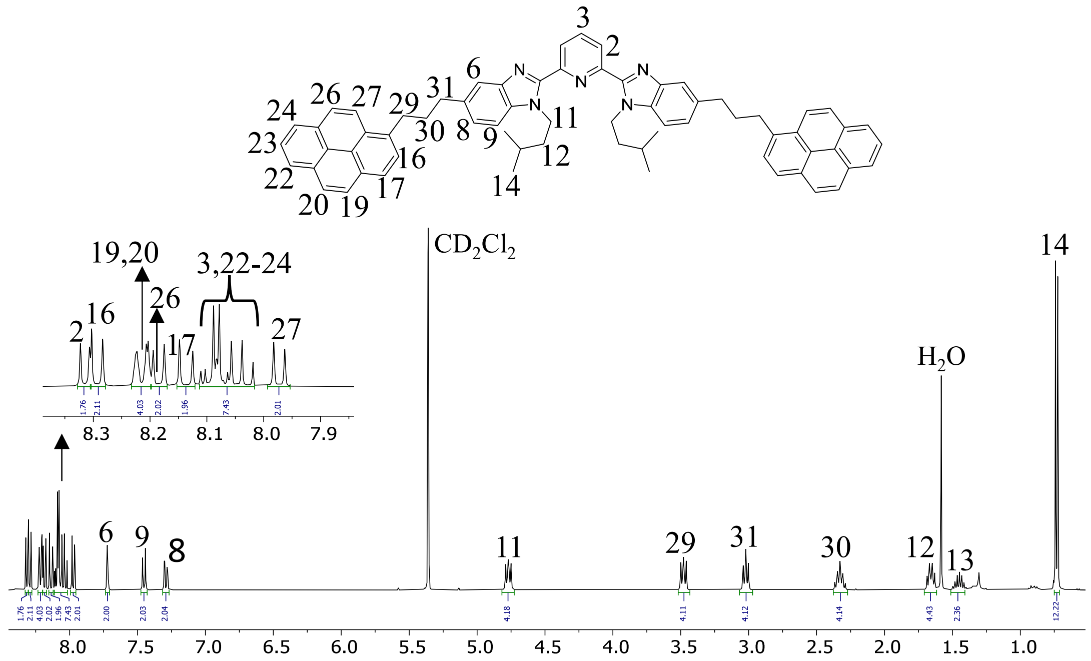

Figure A1-2. ^{1}H NMR spectrum of ligand **L1** in CD_2Cl_2.

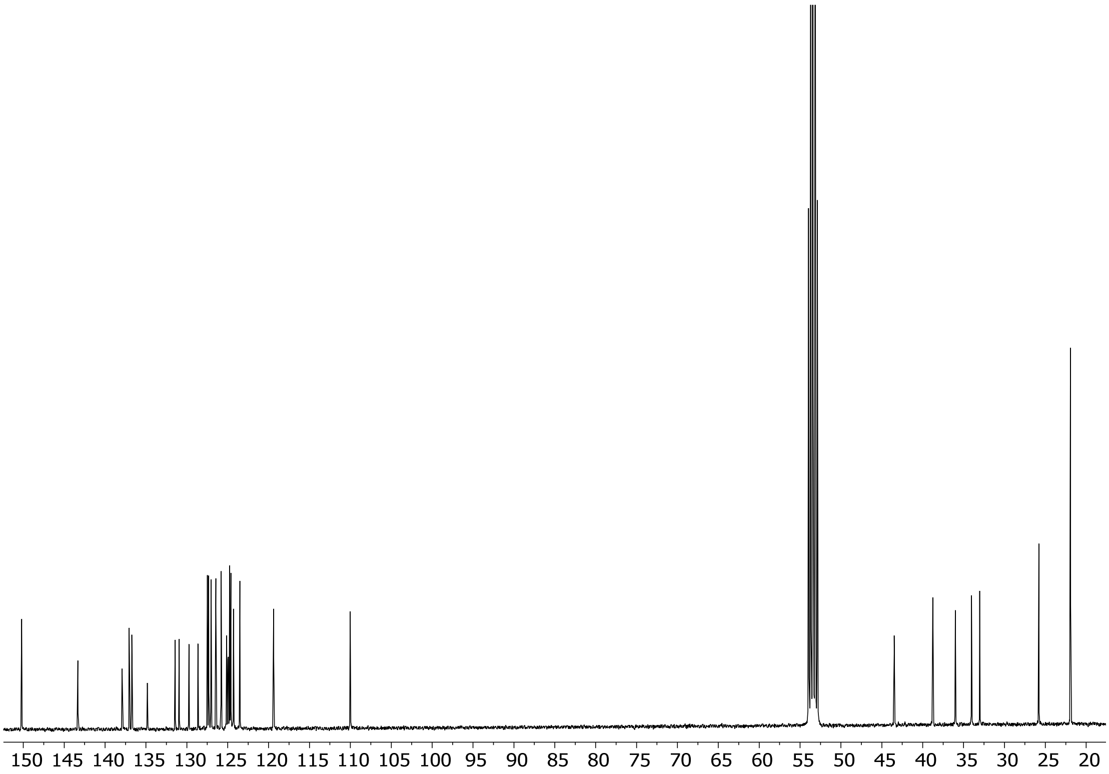

Figure A1-3. ^{13}C NMR spectrum of ligand **L1** in CD_2Cl_2.

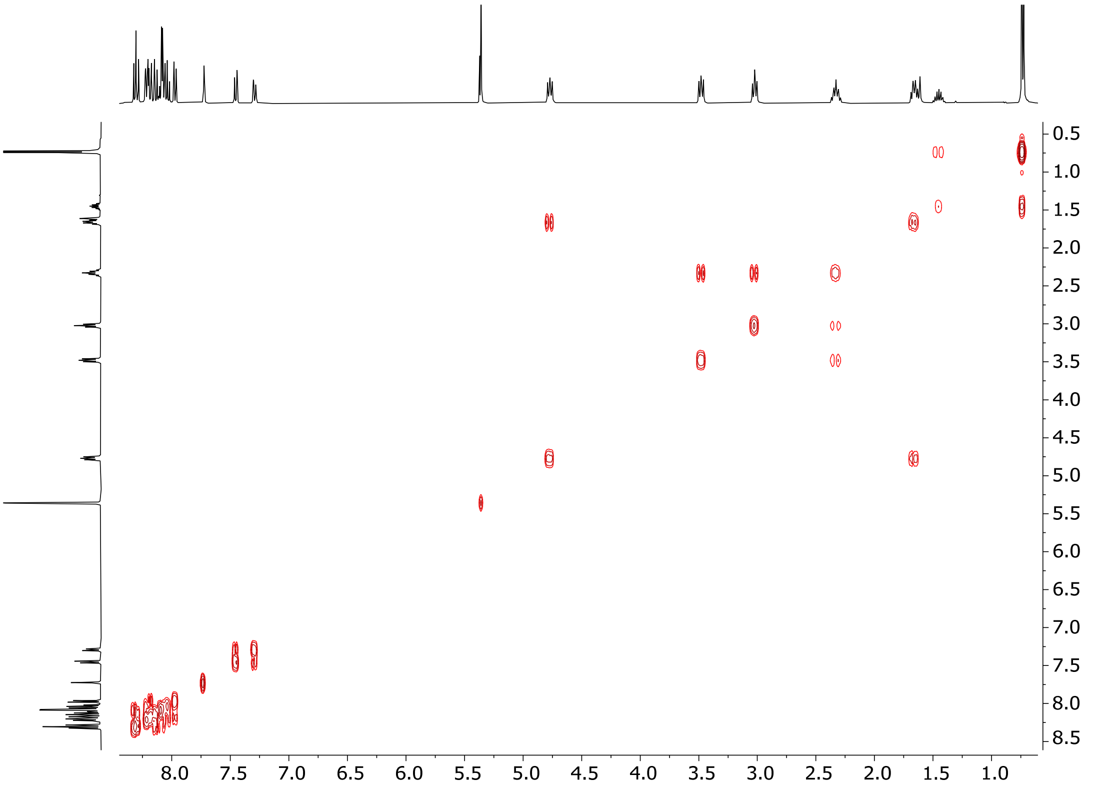

Figure A1-4. COSY spectrum of ligand **L1** in CD_2Cl_2.

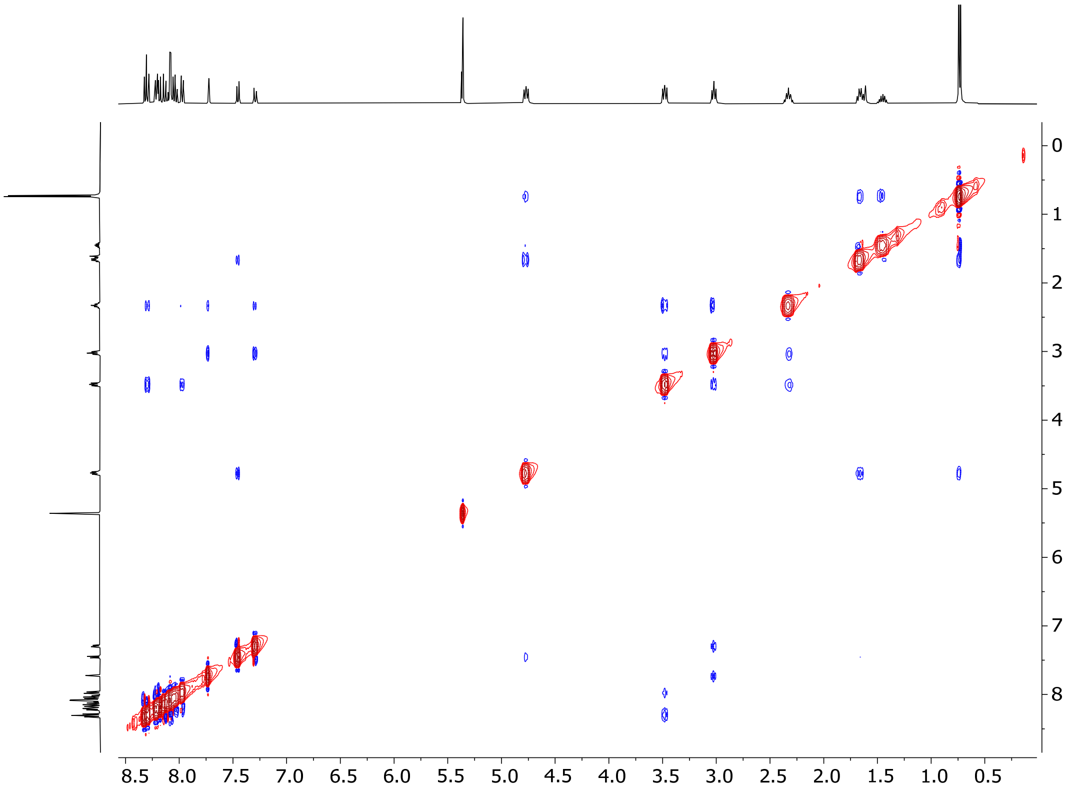

Figure A1-5. NOESY spectrum of ligand **L1** in CD_2Cl_2.

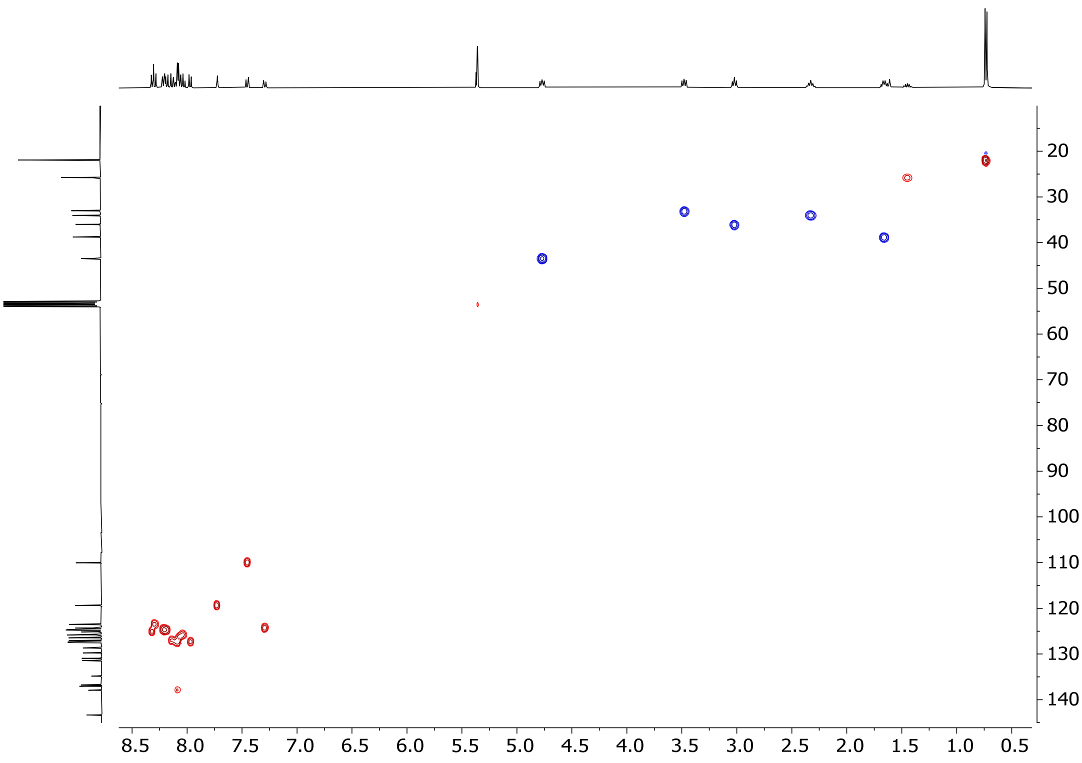

Figure A1-6. HSQC spectrum of ligand **L1** in CD_2Cl_2.

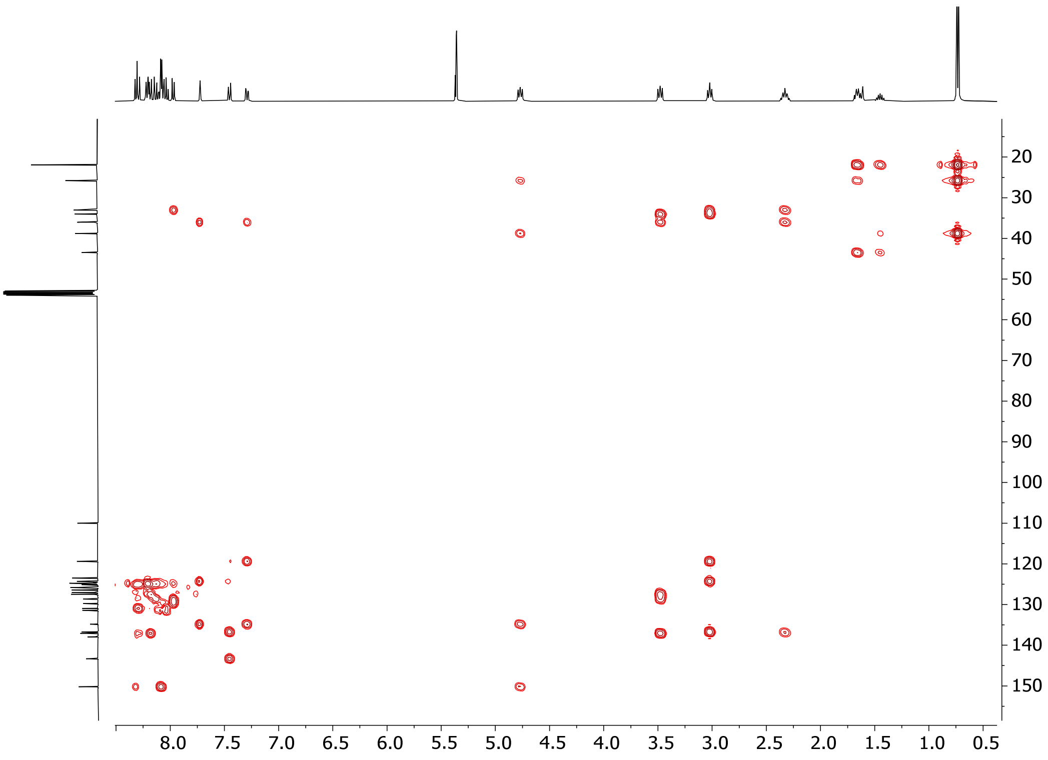

Figure A1-7. HMBC spectrum of ligand **L1** in CD_2Cl_2.

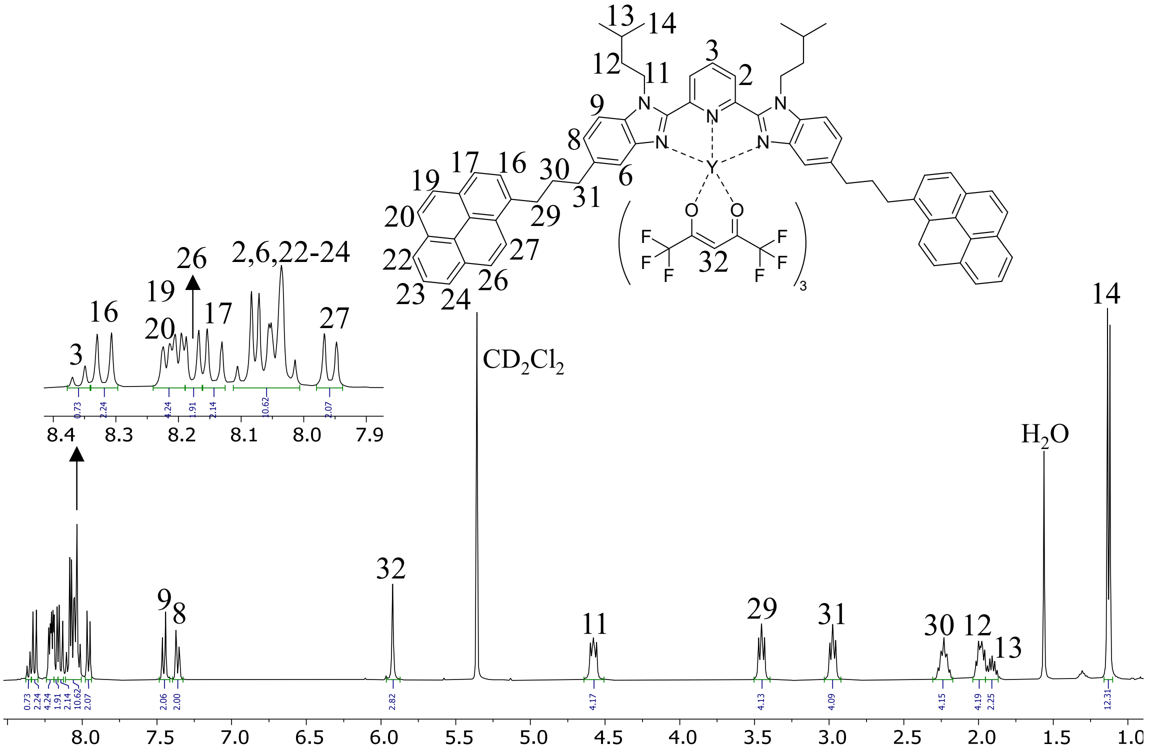

Figure A1-8. ^{1}H NMR spectrum of [**L1**Y(hfac)$_3$] in CD_2Cl_2.

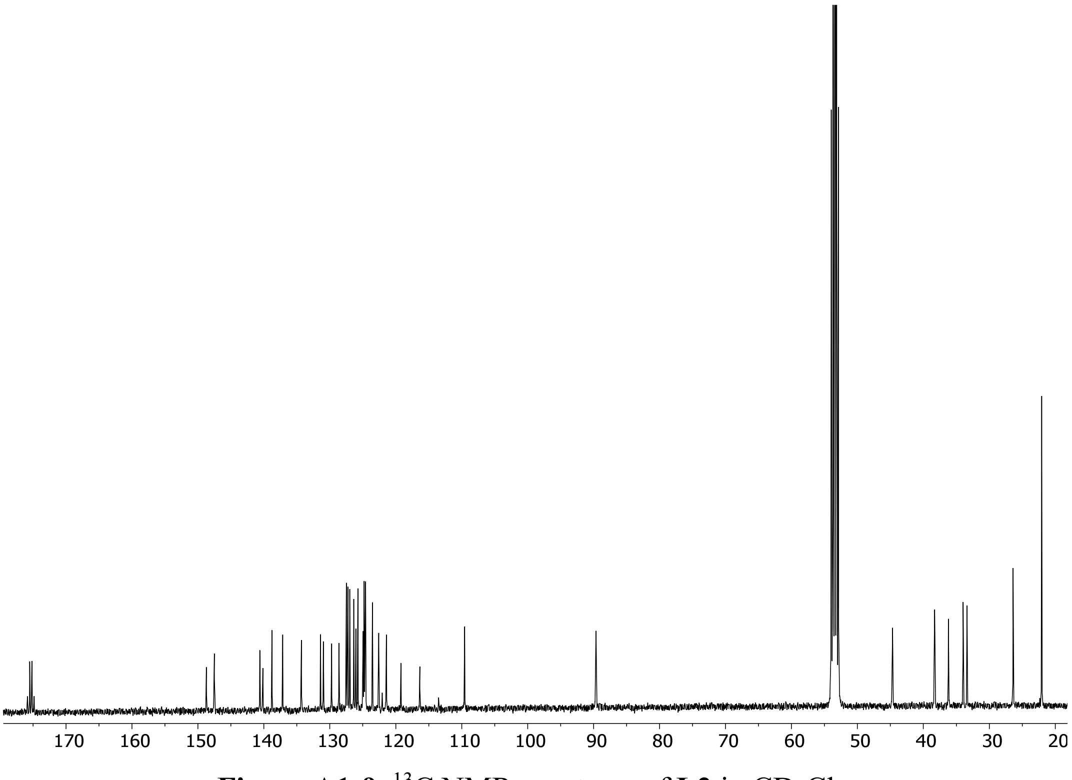

Figure A1-9. ^{13}C NMR spectrum of **L2** in CD_2Cl_2.

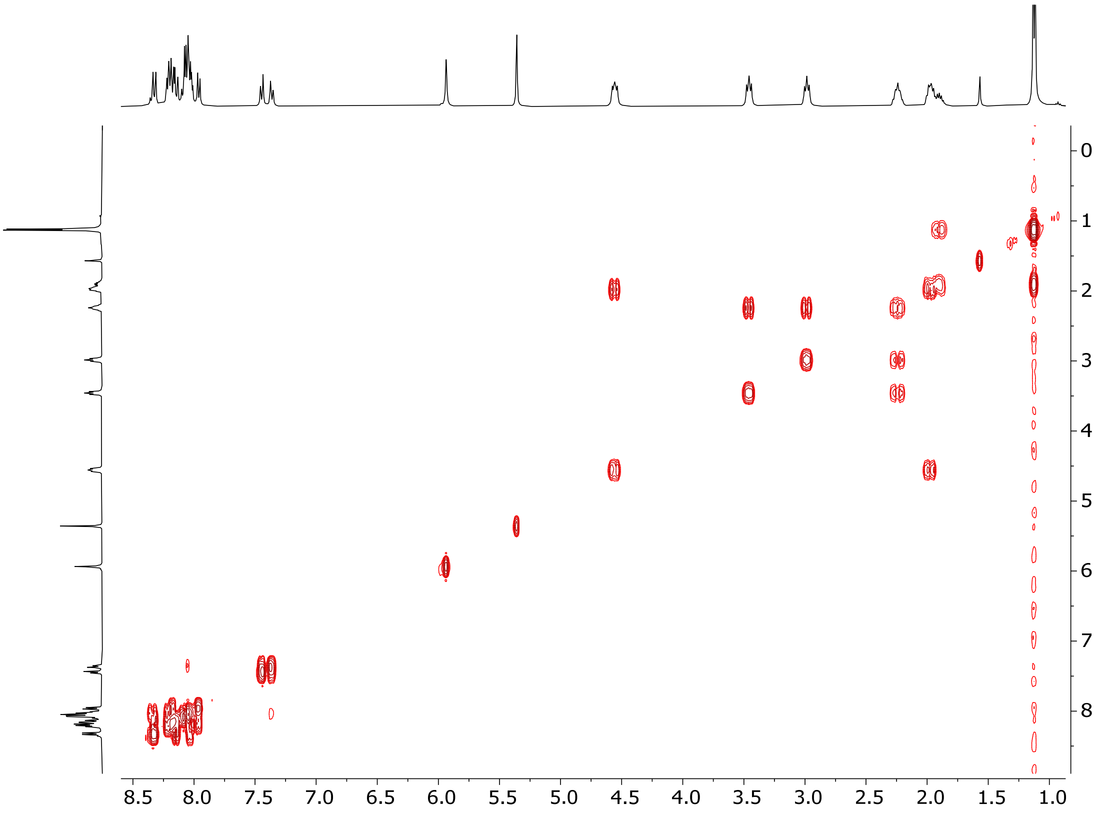

Figure A1-10. COSY spectrum of [**L1**Y(hfac)$_3$] in CD_2Cl_2.

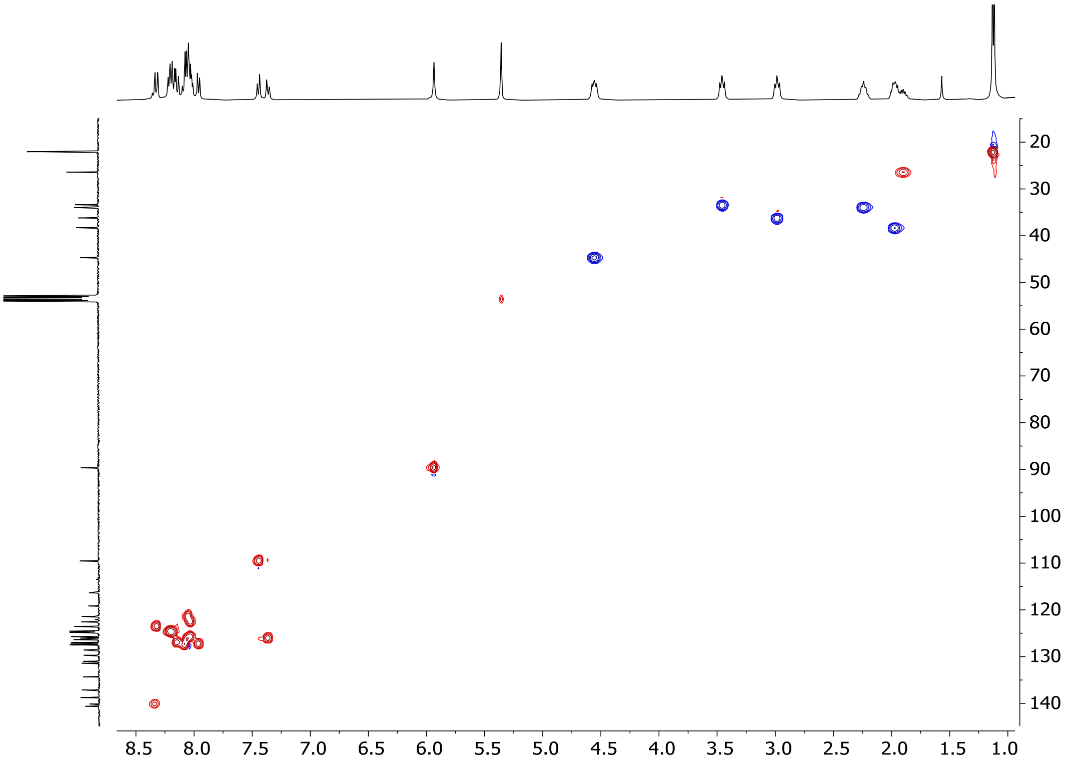

Figure A1-11. HSQC spectrum of [**L1**Y(hfac)$_3$] in CD_2Cl_2.

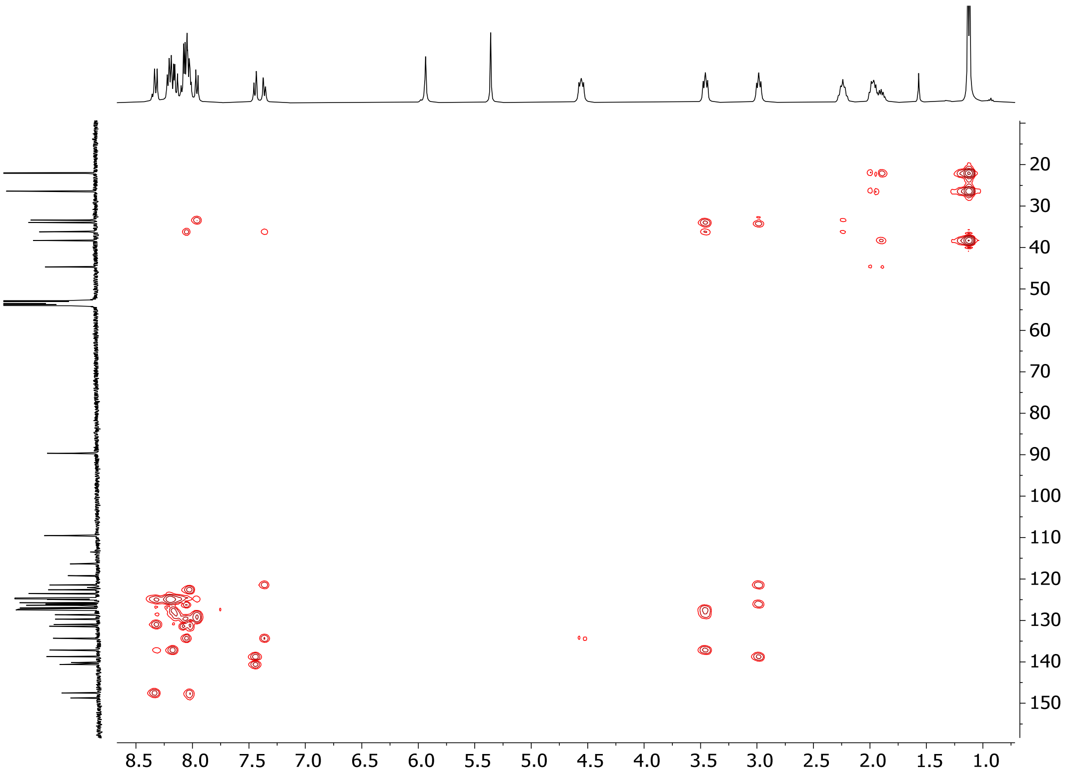

Figure A1-12. HMBC spectrum of [**L1**Y(hfac)$_3$] in CD_2Cl_2.

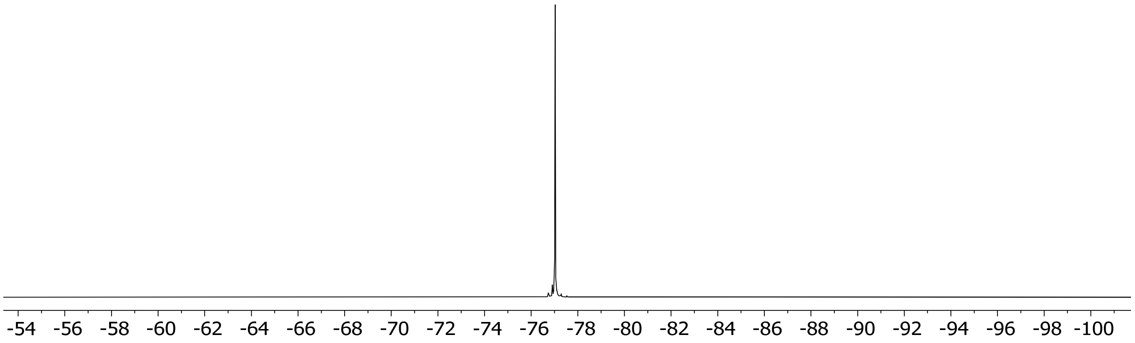

Figure A1-13. ^{19}F NMR spectrum of [**L1**Y(hfac)$_3$] in CD_2Cl_2.

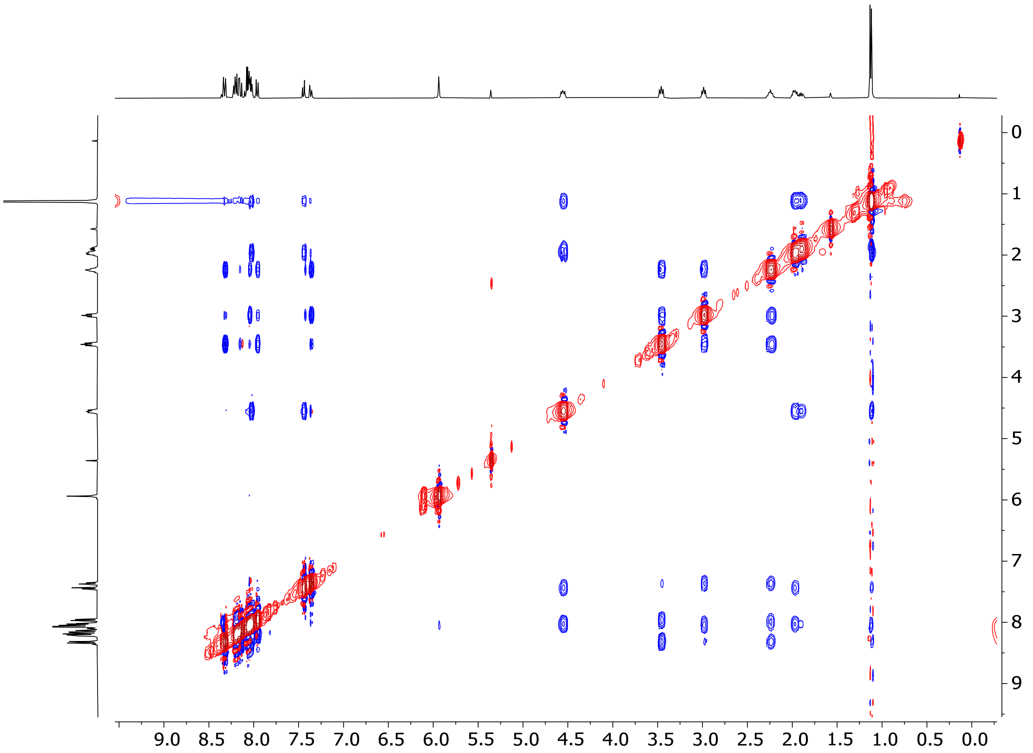

Figure A1-14. NOESY spectrum of [**L1**Y(hfac)$_3$] at 0.02 M in CD_2Cl_2.

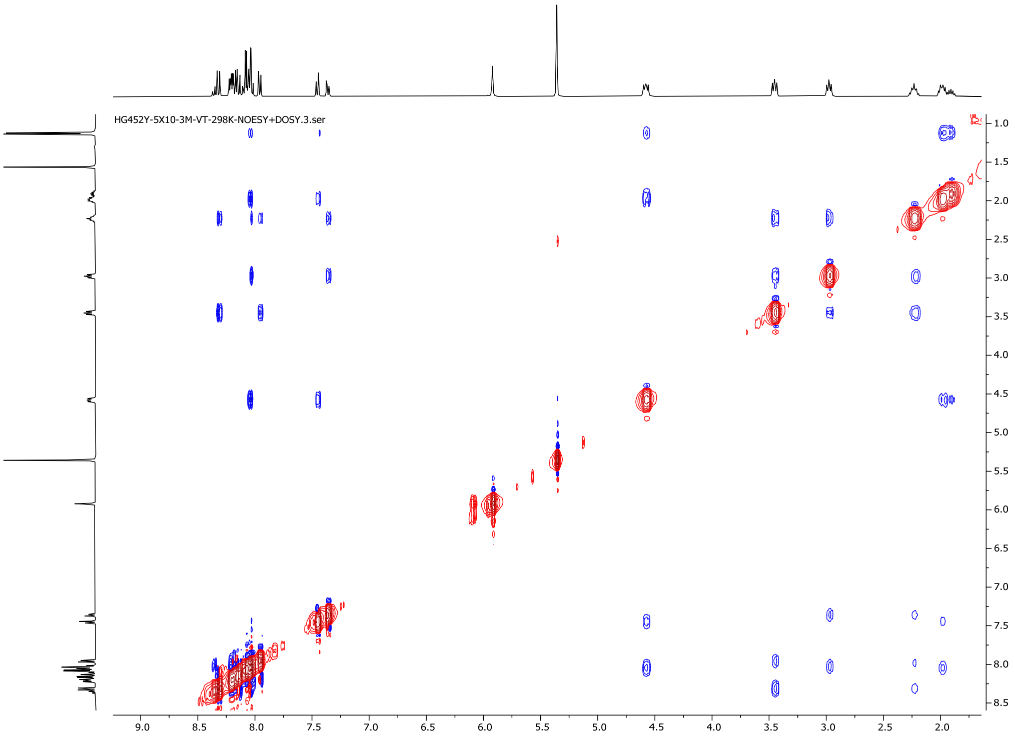

Figure A1-15. NOESY spectrum of [**L1**Y(hfac)$_3$] at 0.005 M in CD_2Cl_2.

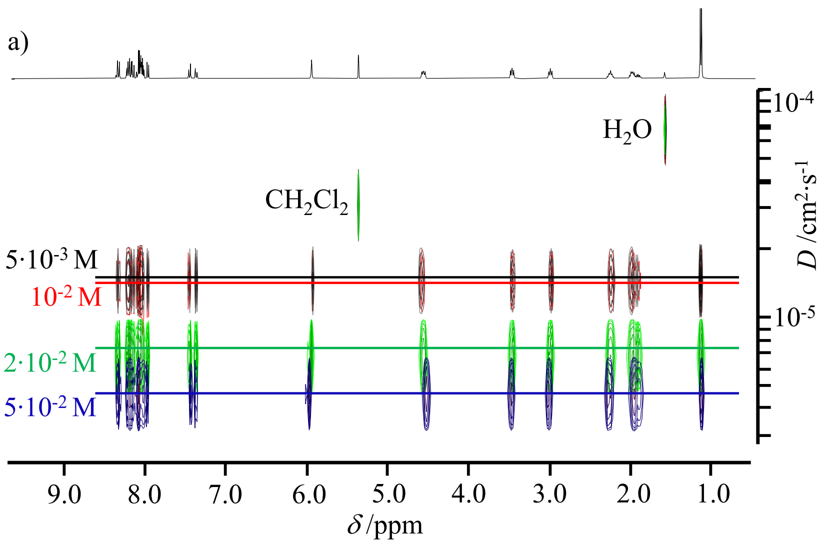

Figure A1-16. DOSY spectrum of [**L1**Y(hfac)$_3$] in CD_2Cl_2.

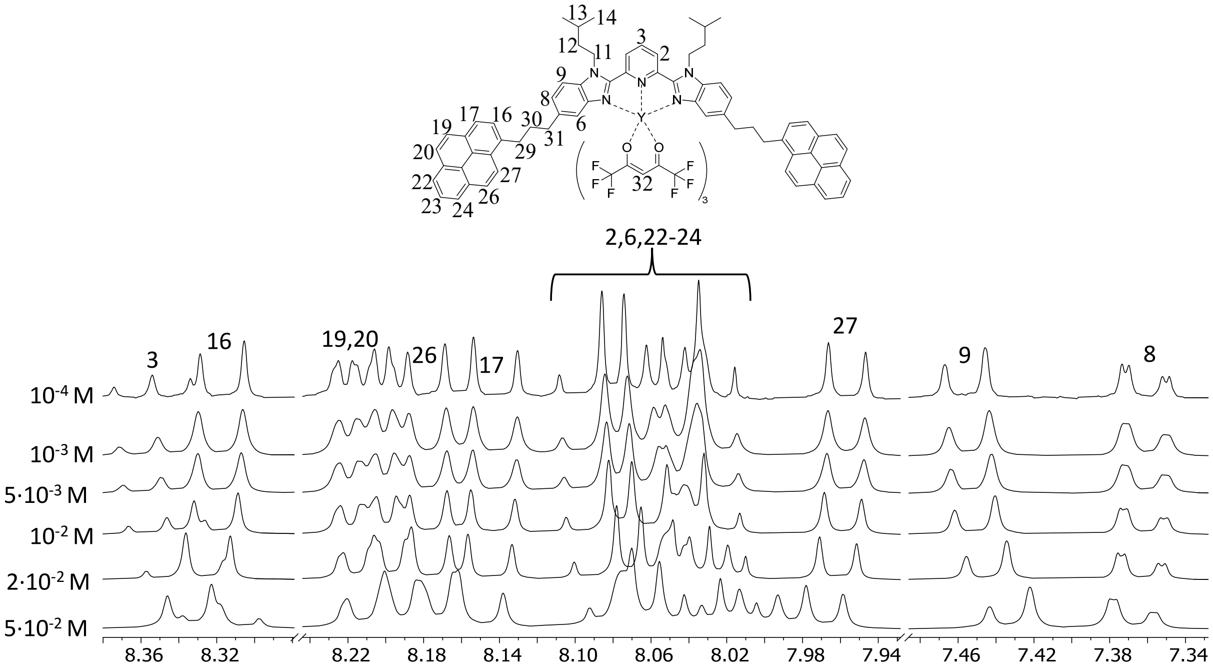

Figure A1-17. ^{1}H NMR spectrum of [**L1**Y(hfac)$_3$] in CD_2Cl_2 upon increasing concentration.

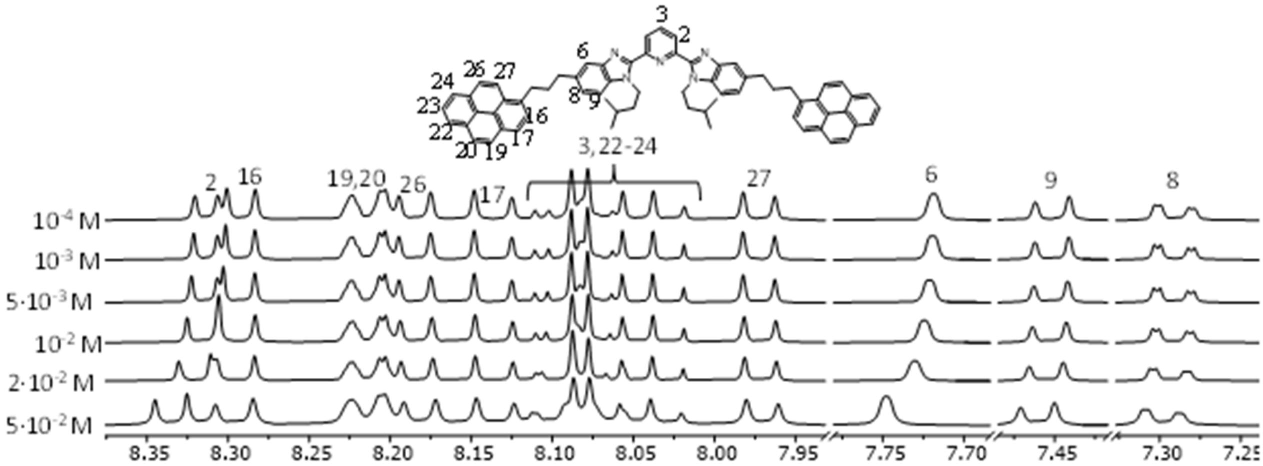

Figure A1-18. ^{1}H NMR spectrum of **L1** in CD_2Cl_2 upon increasing concentration.

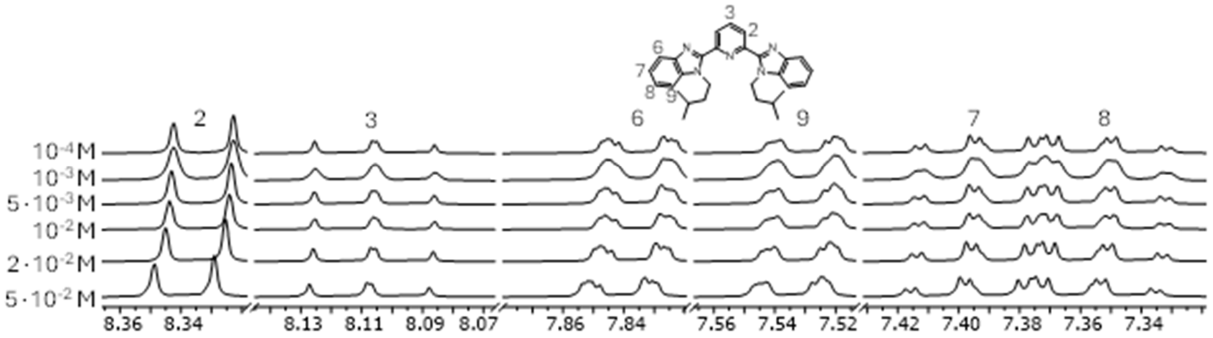

Figure A1-19. ^{1}H NMR spectrum of **L0** in CD_2Cl_2 upon increasing concentration.

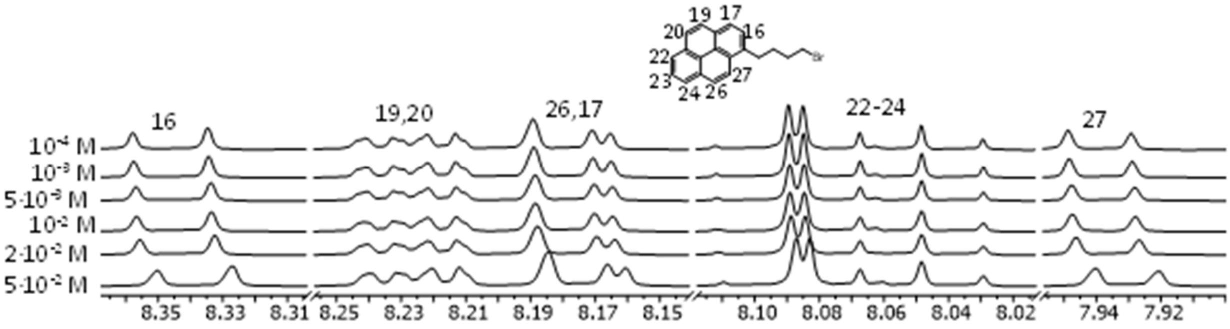

Figure A1-20. ^{1}H NMR spectrum of **1** in CD_2Cl_2 upon increasing concentration.

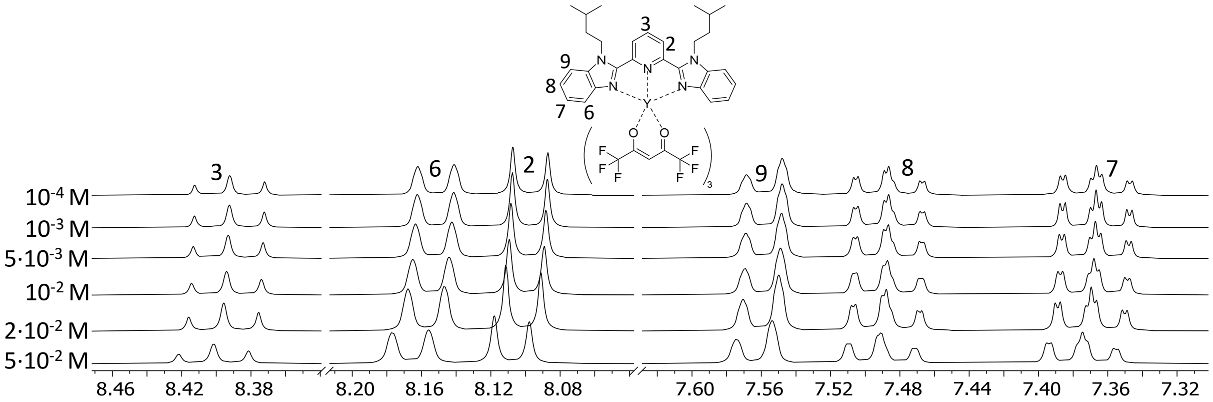

Figure A1-21. ^{1}H NMR spectrum of [**L0**Y(hfac)$_3$] in CD_2Cl_2 upon increasing concentration.

HG452Eu-all.7.fid

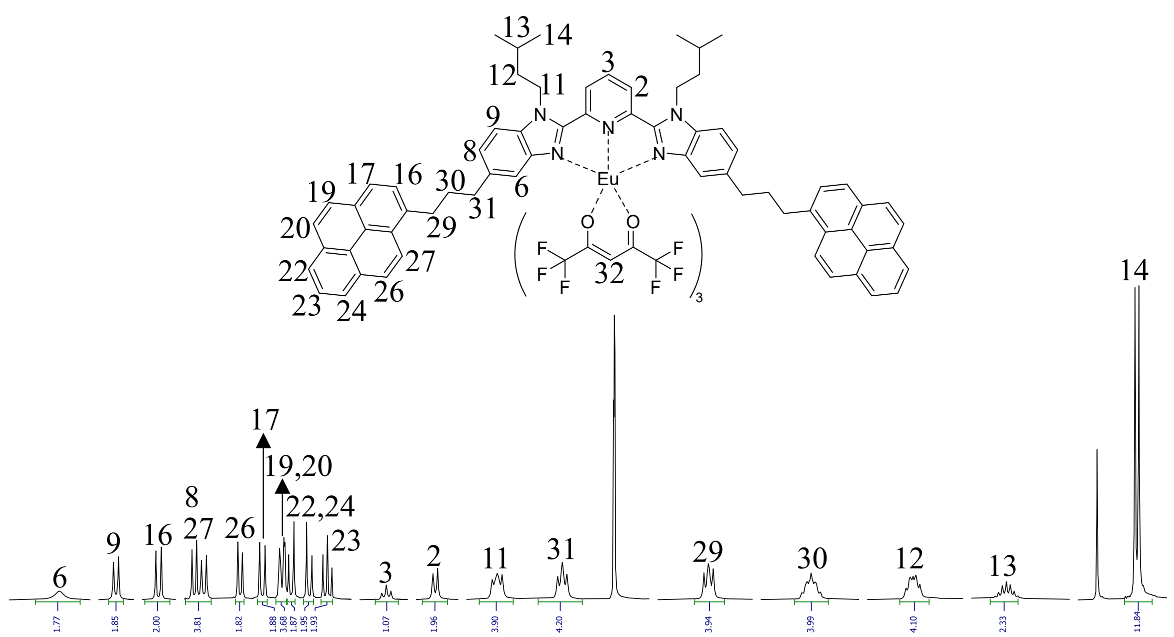

Figure A1-22. ^{1}H NMR spectrum of [**L1**Eu(hfac)$_3$] in CD_2Cl_2.

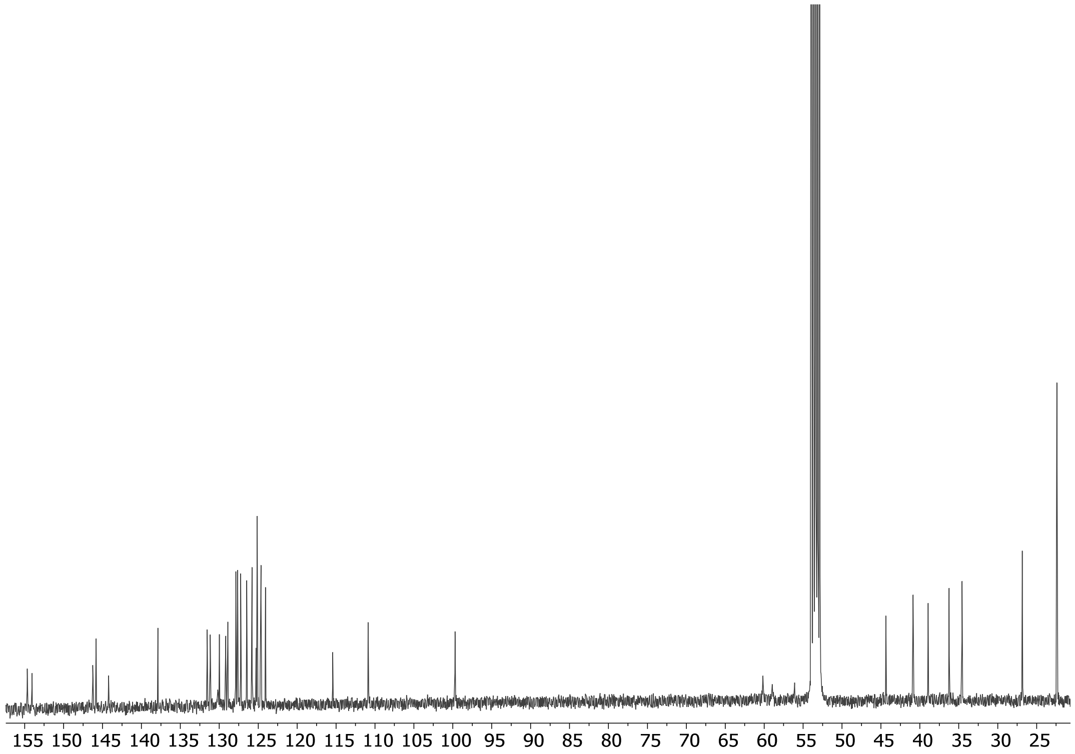

Figure A1-23. ^{13}C NMR spectrum of [**L1**Eu(hfac)$_3$] in CD_2Cl_2.

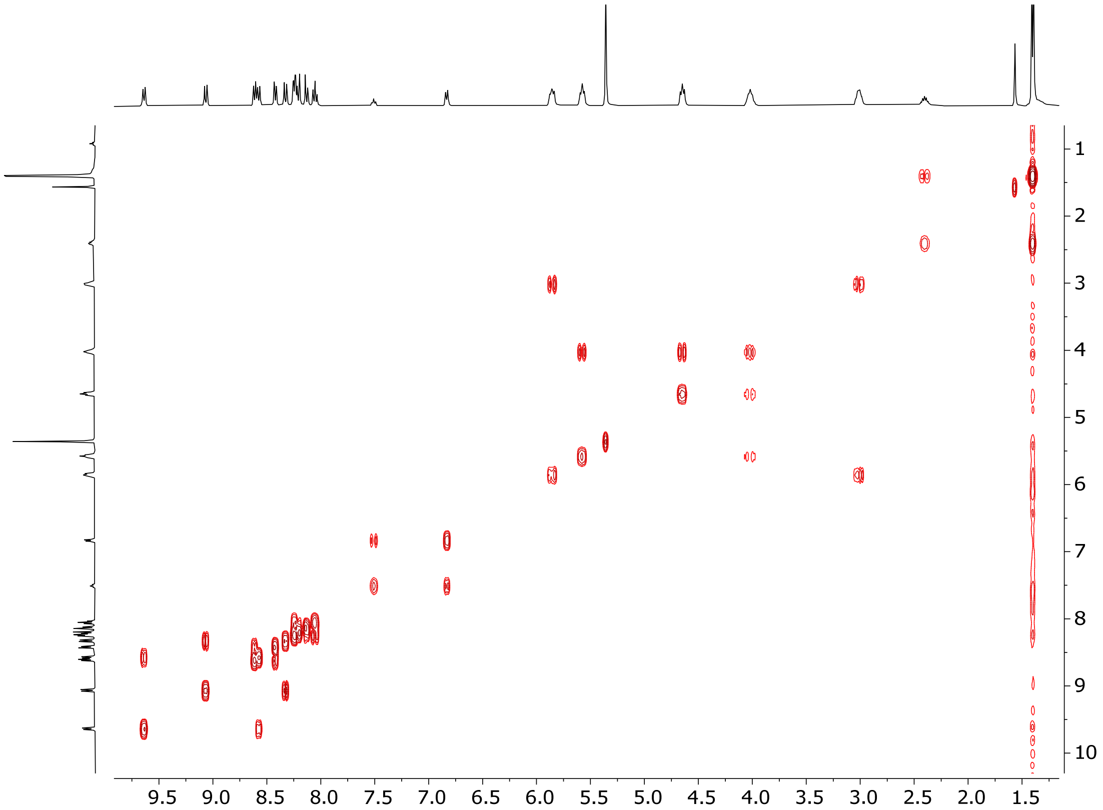

Figure A1-24. COSY spectrum of [**L1**Eu(hfac)$_3$] in CD_2Cl_2.

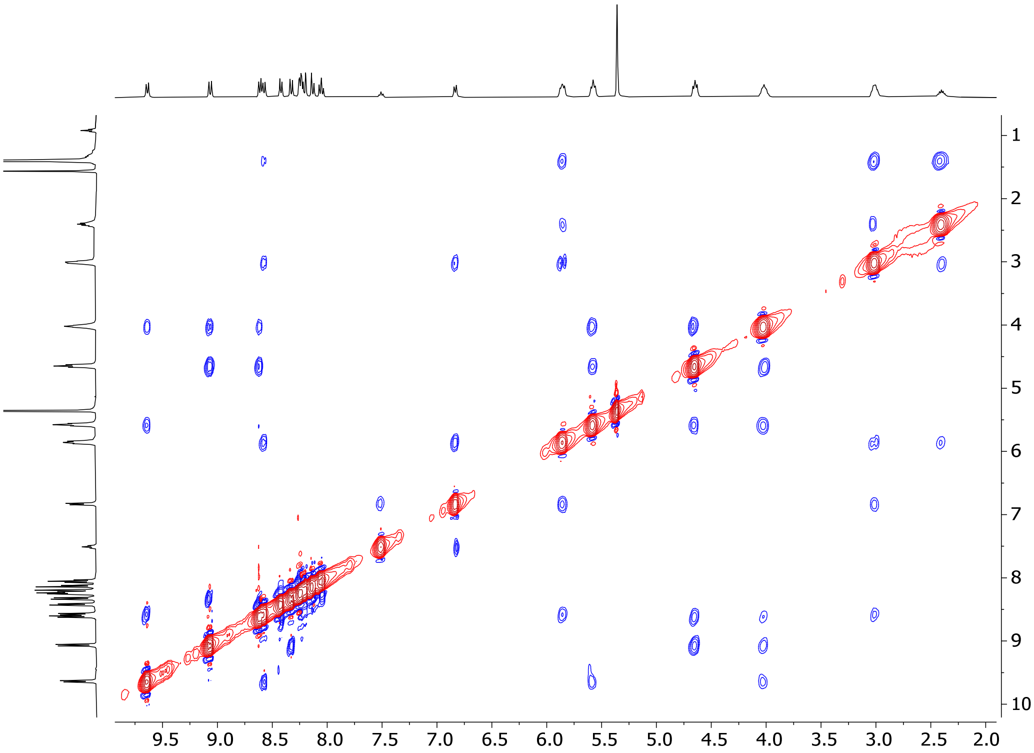

Figure A1-25. NOESY spectrum of [**L1**Eu(hfac)$_3$] in CD_2Cl_2.

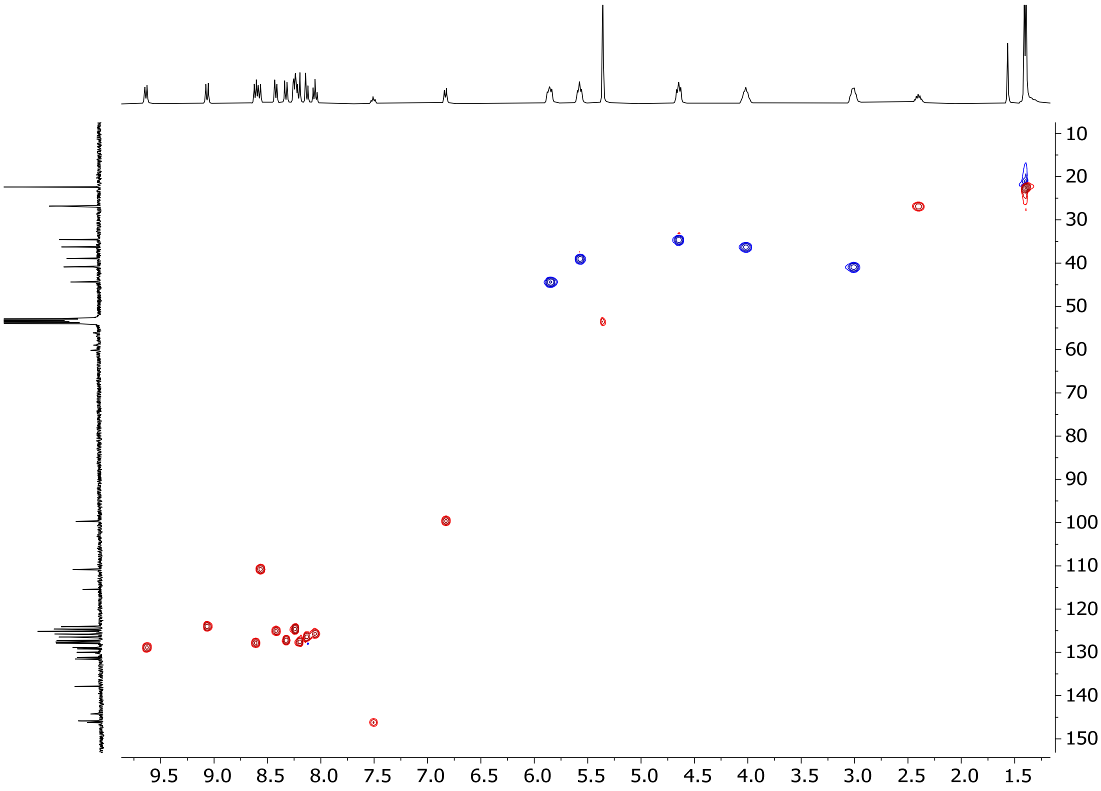

Figure A1-26. HSQC spectrum of [**L1**Eu(hfac)$_3$] in CD_2Cl_2.

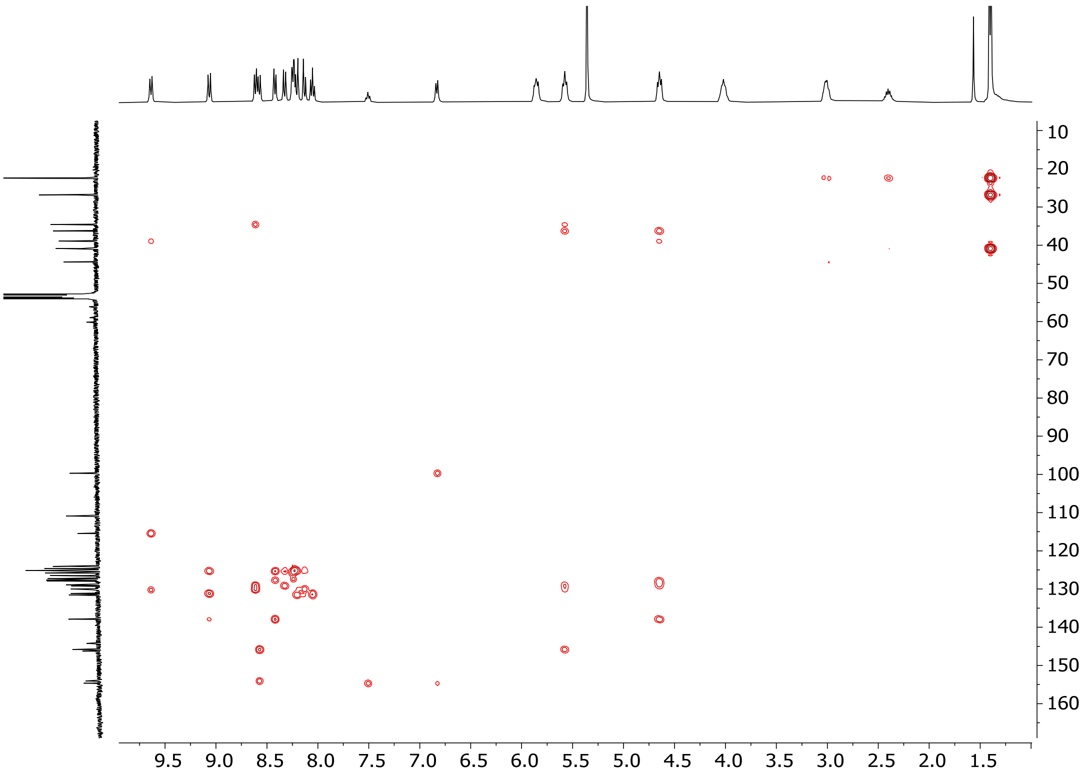

Figure A1-27. HMBC spectrum of [**L1**Eu(hfac)$_3$] in CD_2Cl_2.

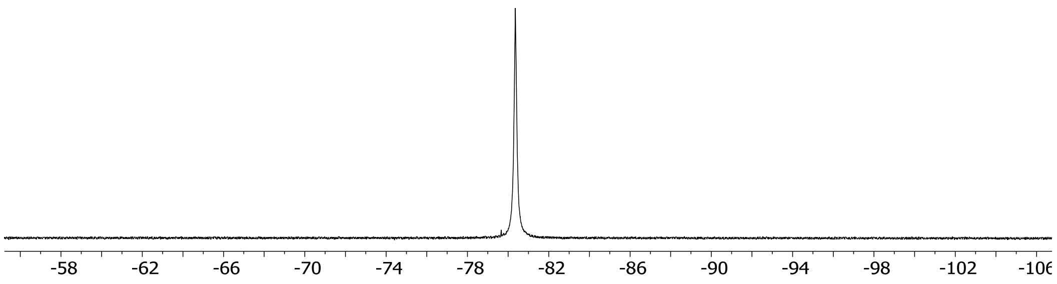

Figure A1-28. ^{19}F NMR spectrum of [**L1**Eu(hfac)$_3$] in CD_2Cl_2.

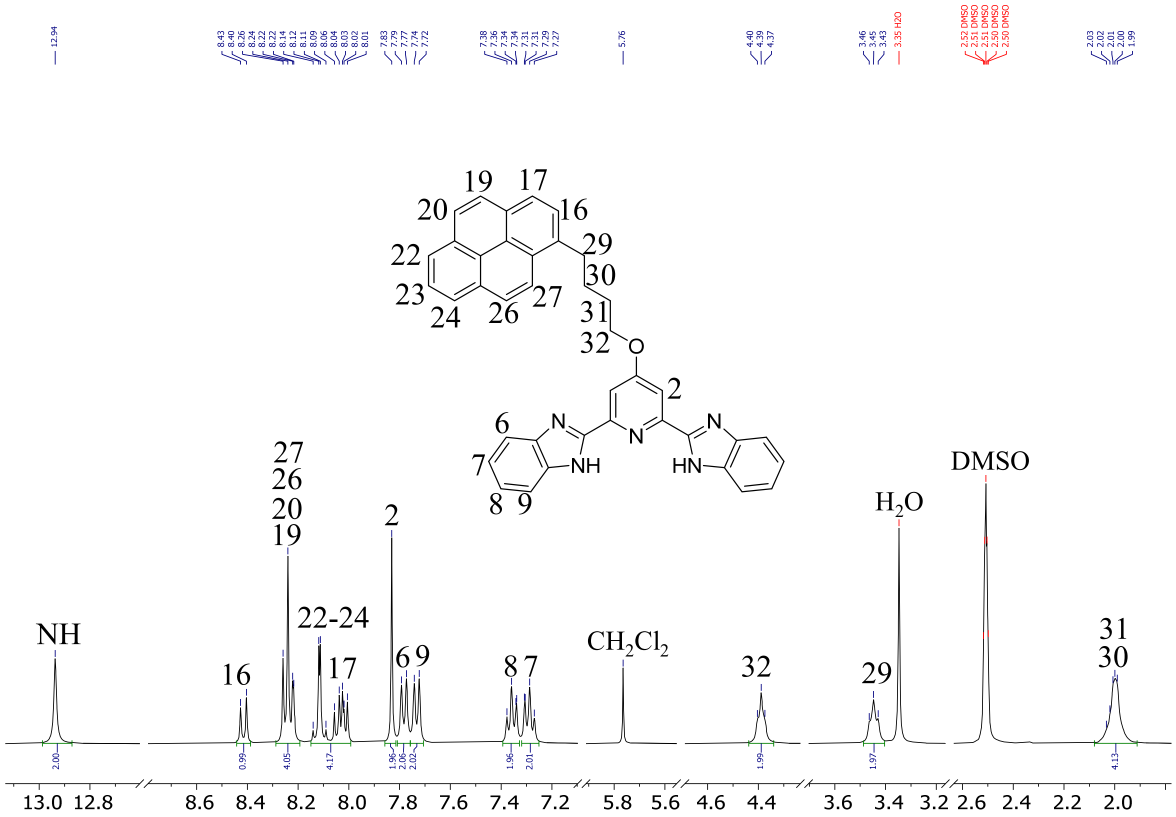

Figure A1-29. ^{1}H NMR spectrum of **3** in DMSO-*d6*.

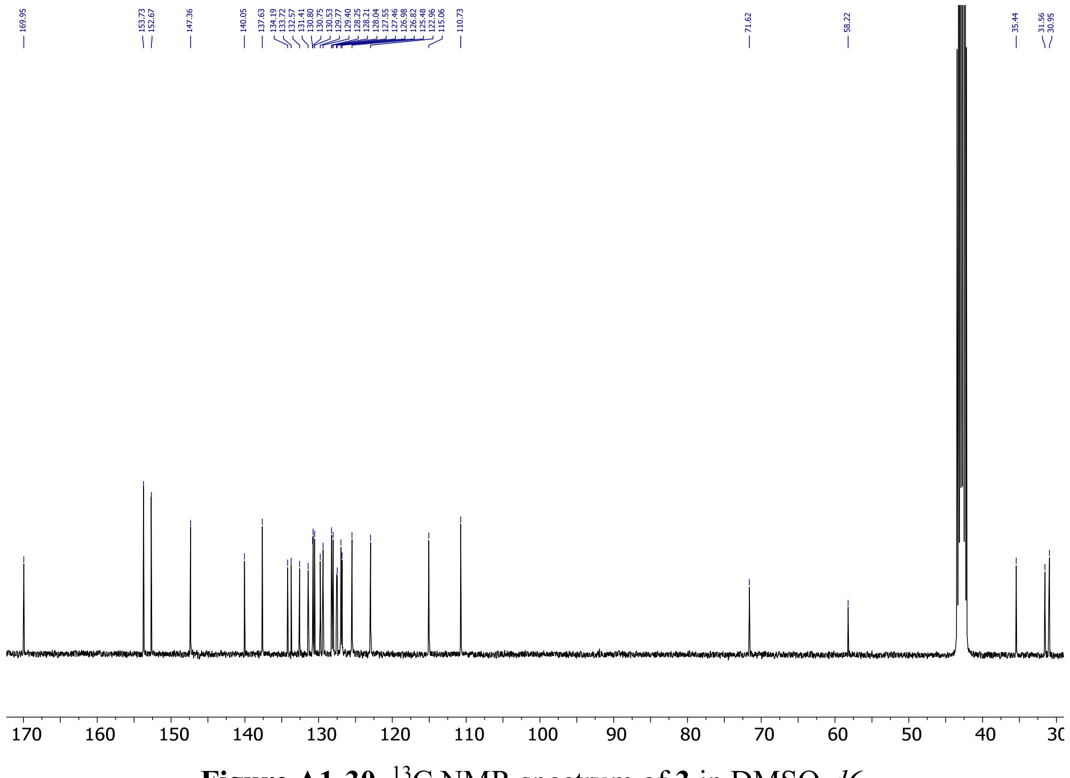

Figure A1-30. ^{13}C NMR spectrum of **3** in DMSO-*d6*.

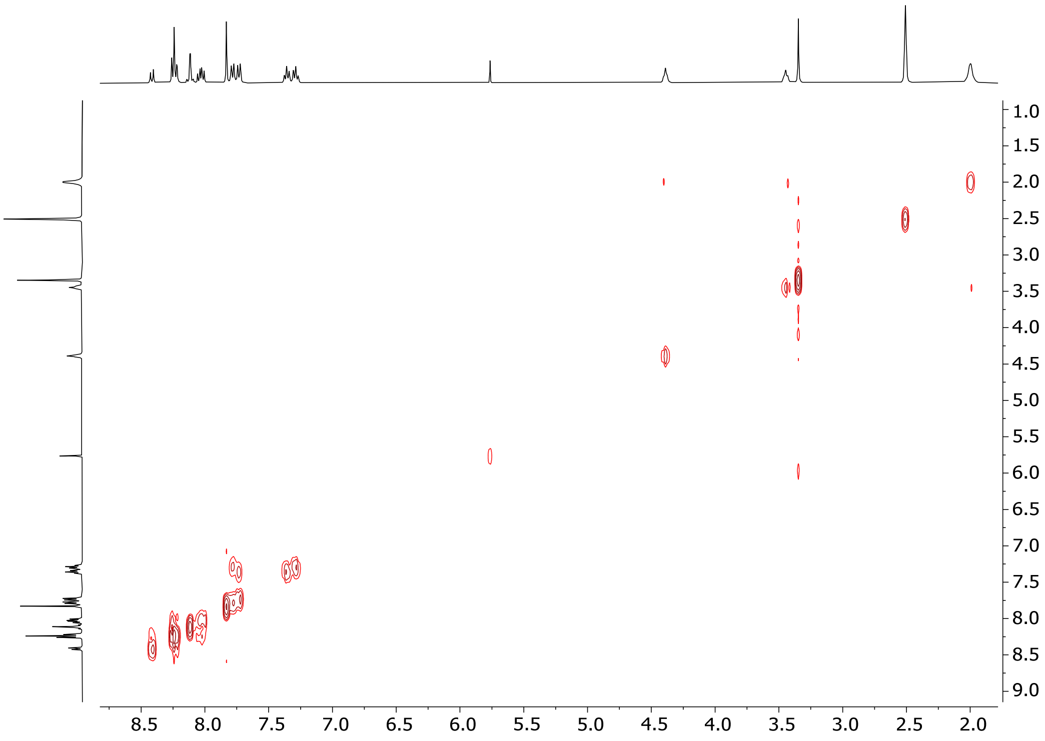

Figure A1-31. COSY spectrum of **3** in DMSO-*d6*.

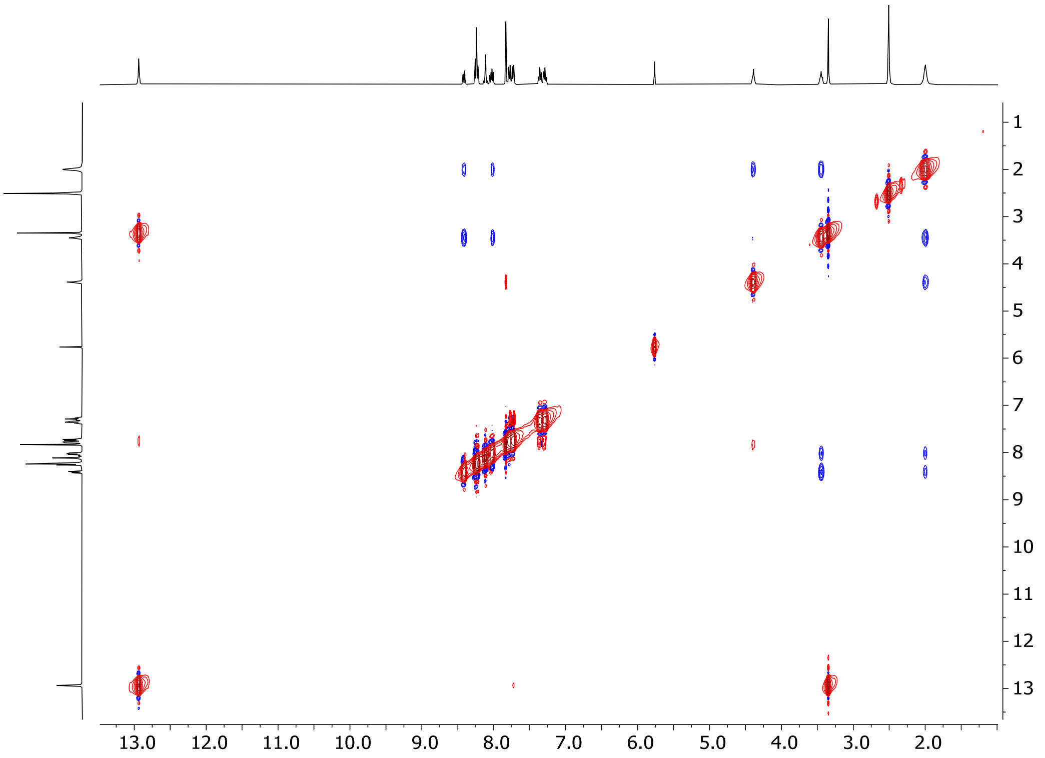

Figure A1-32. NOESY spectrum of **3** in DMSO-*d6*.

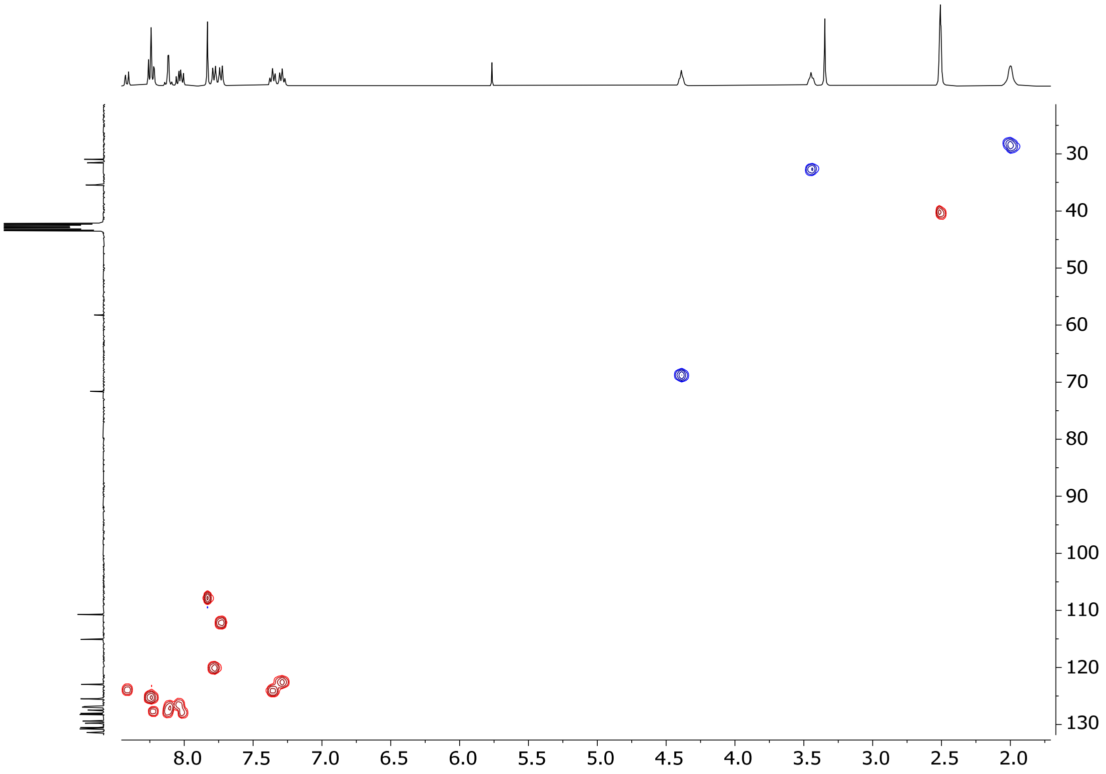

Figure A1-33. HSQC spectrum of **3** in DMSO-*d6*.

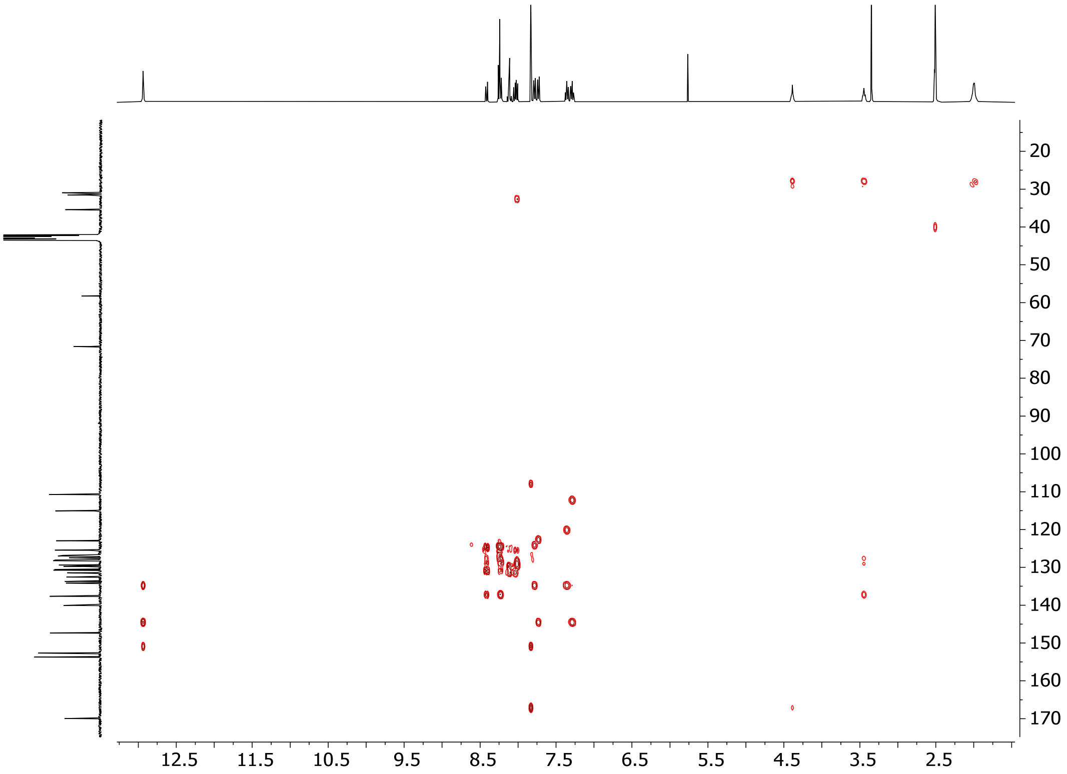

Figure A1-34. HMBC spectrum of **3** in DMSO-*d6*.

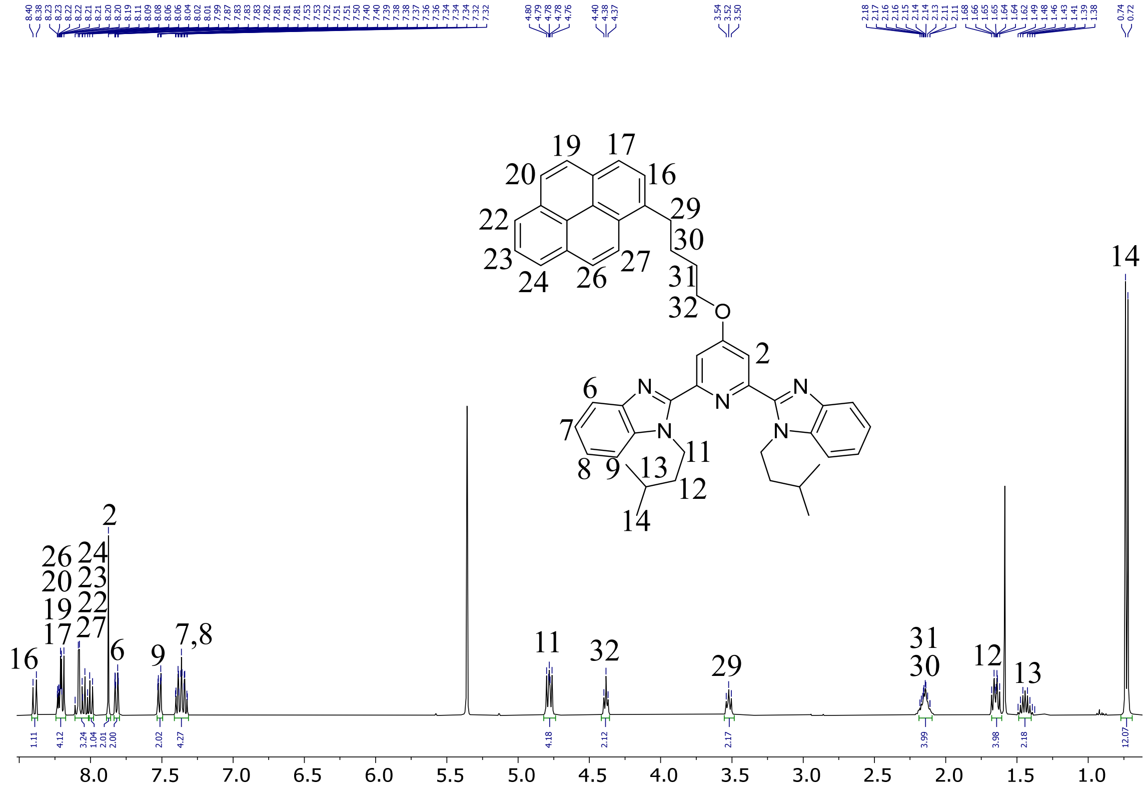

Figure A1-35. ^{1}H NMR spectrum of **L2** in CD_2Cl_2.

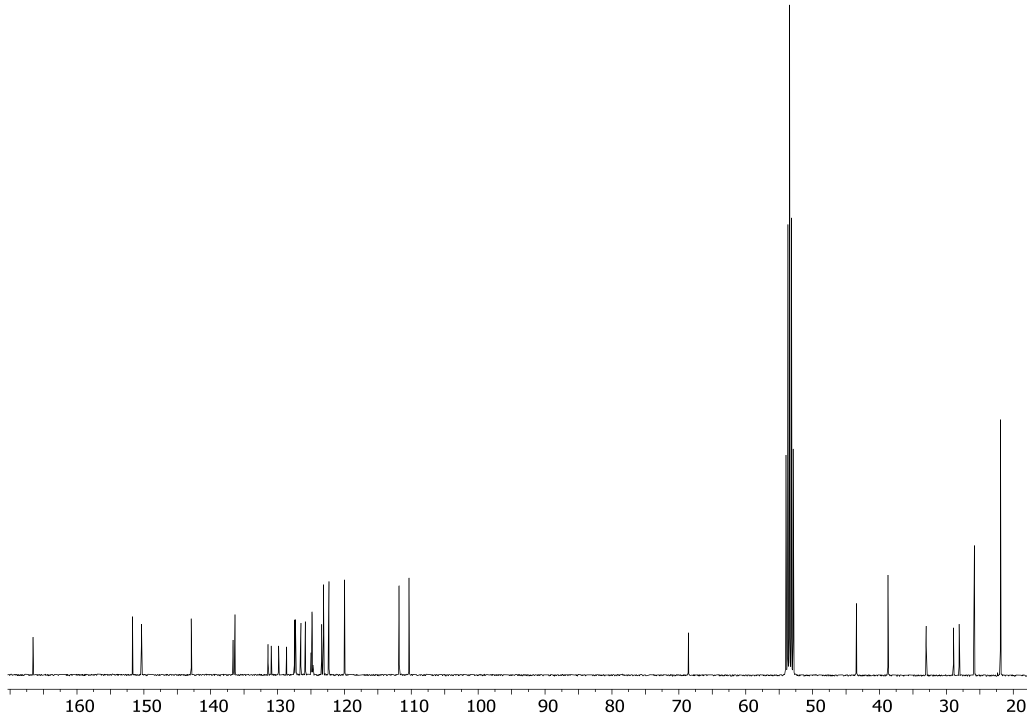

Figure A1-36. ^{13}C NMR spectrum of **L2** in CD_2Cl_2.

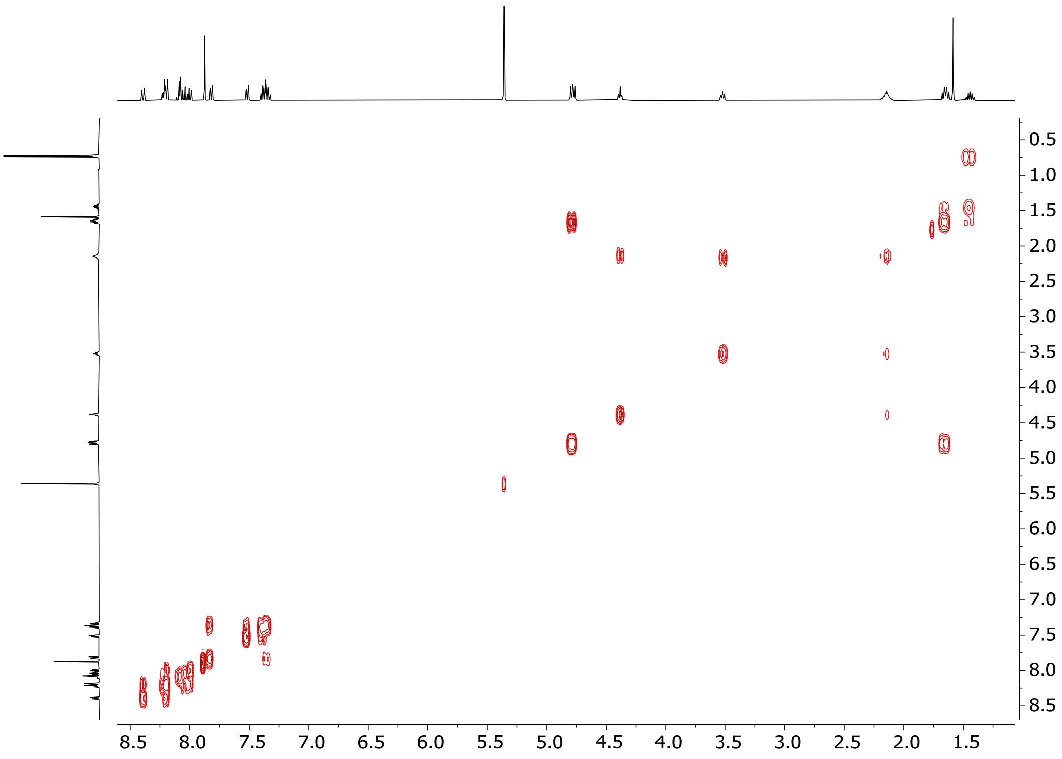

Figure A1-37. COSY spectrum of **L2** in CD_2Cl_2.

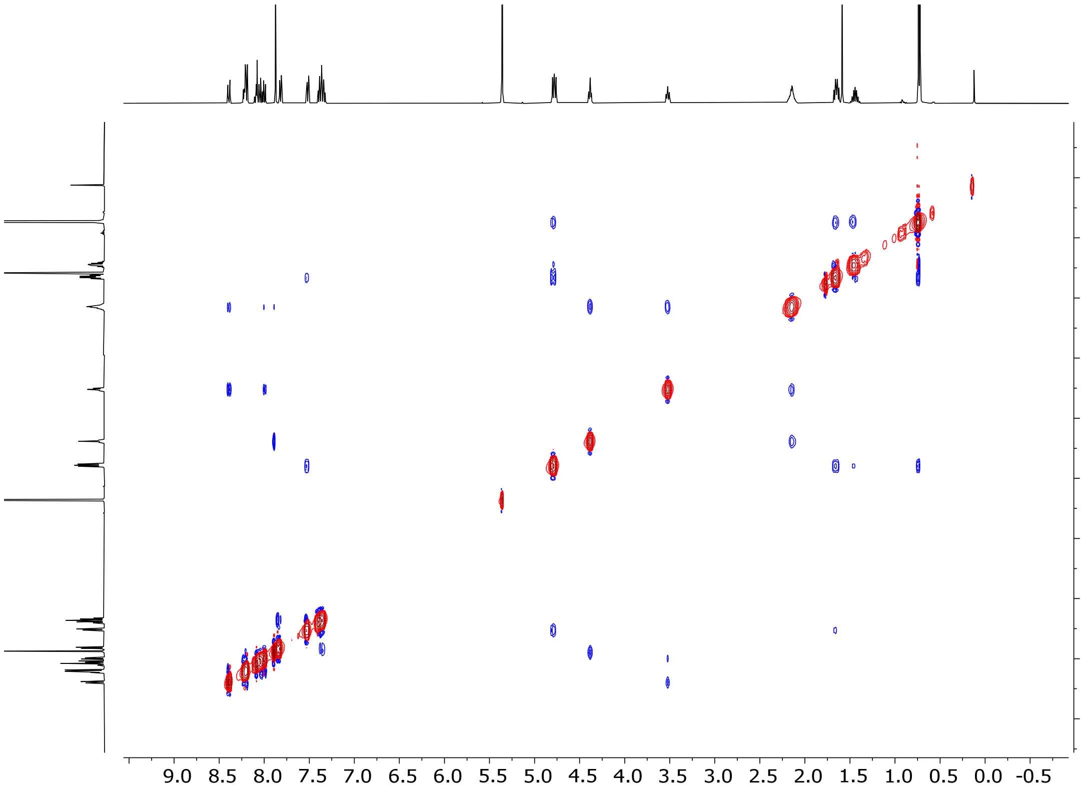

Figure A1-38. NOESY spectrum of **L2** in CD_2Cl_2.

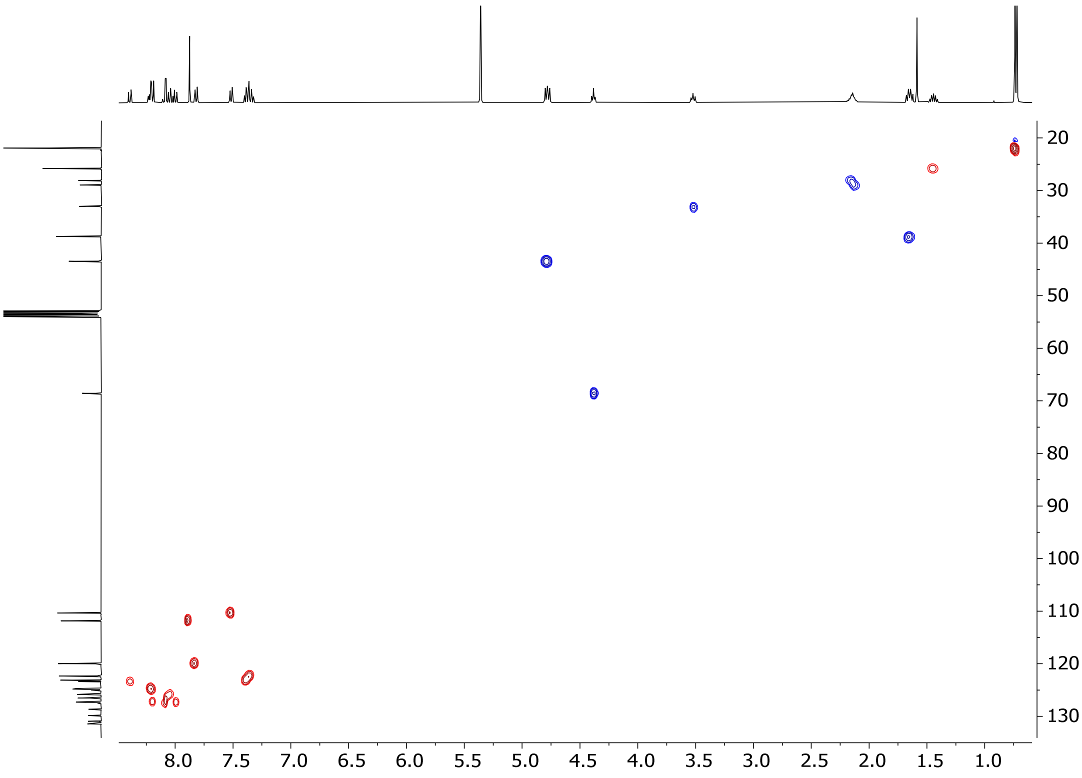

Figure A1-39. HSQC spectrum of **L2** in CD_2Cl_2.

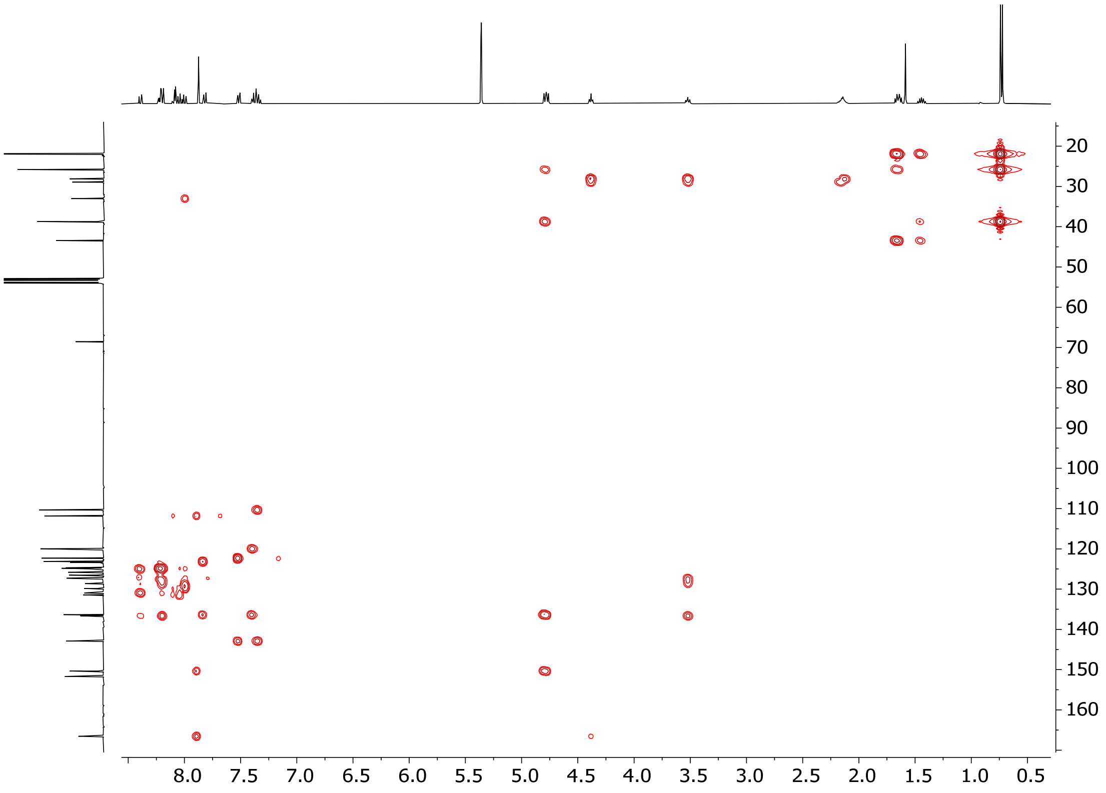

Figure A1-40. HMBC spectrum of **L2** in CD_2Cl_2.

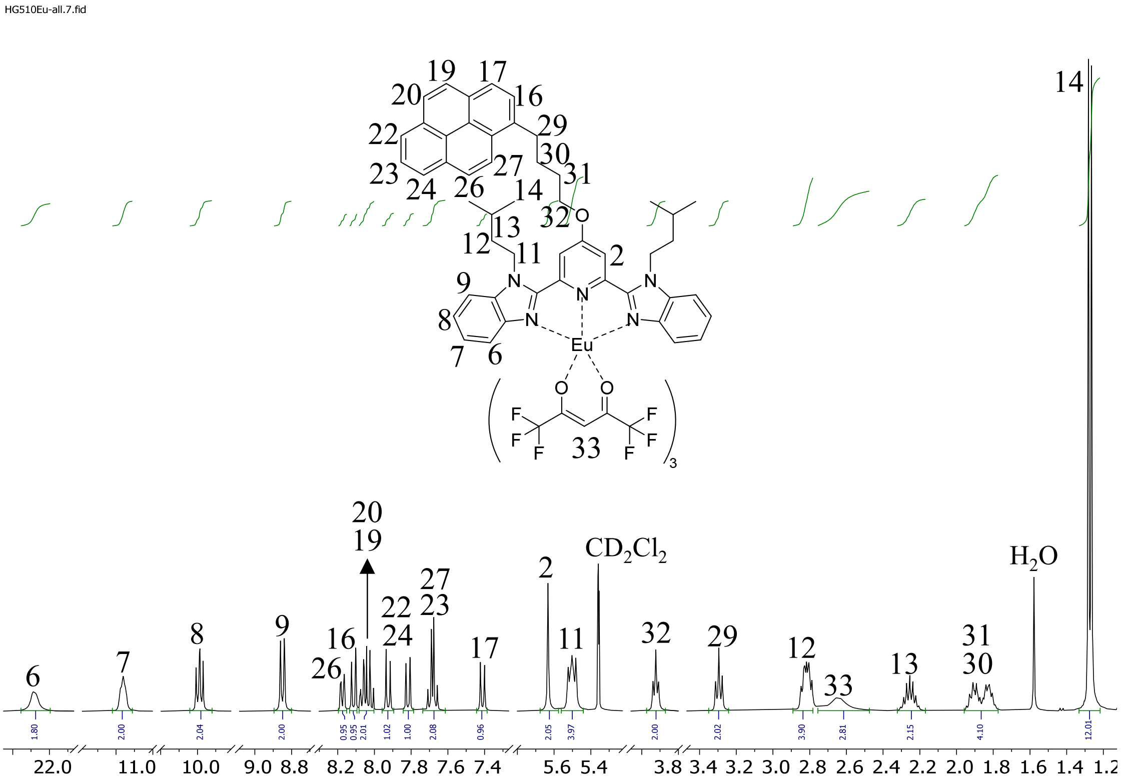

Figure A1-41. ^{1}H NMR spectrum of [**L2**Eu(hfac)$_3$] in CD$_2$Cl$_2$.

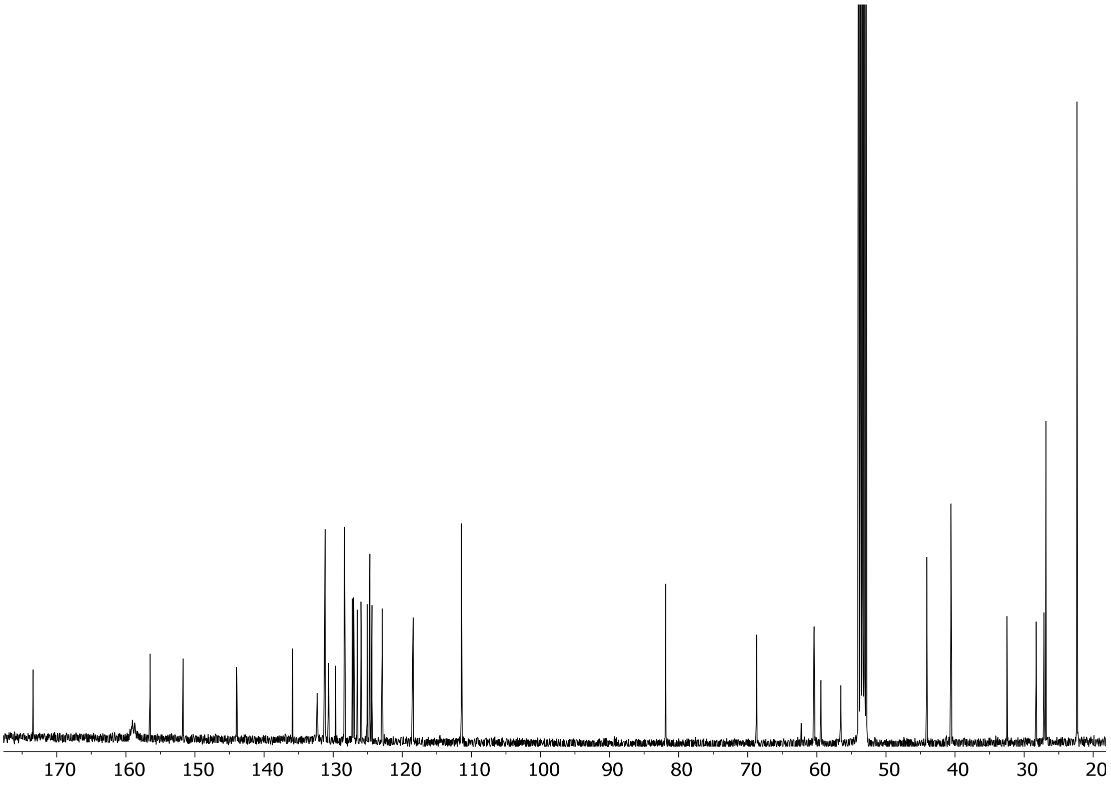

Figure A1-42. ^{13}C NMR spectrum of [**L2**Eu(hfac)$_3$] in CD$_2$Cl$_2$.

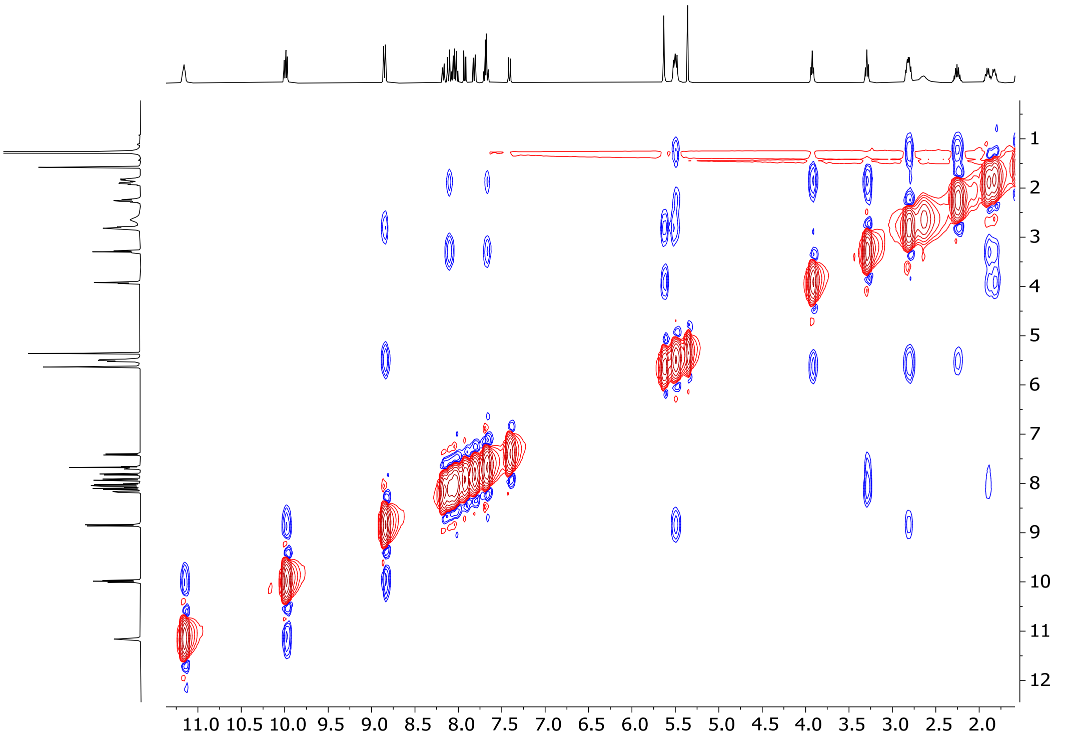

Figure A1-43. NOESY spectrum of [**L2**Eu(hfac)$_3$] in CD_2Cl_2.

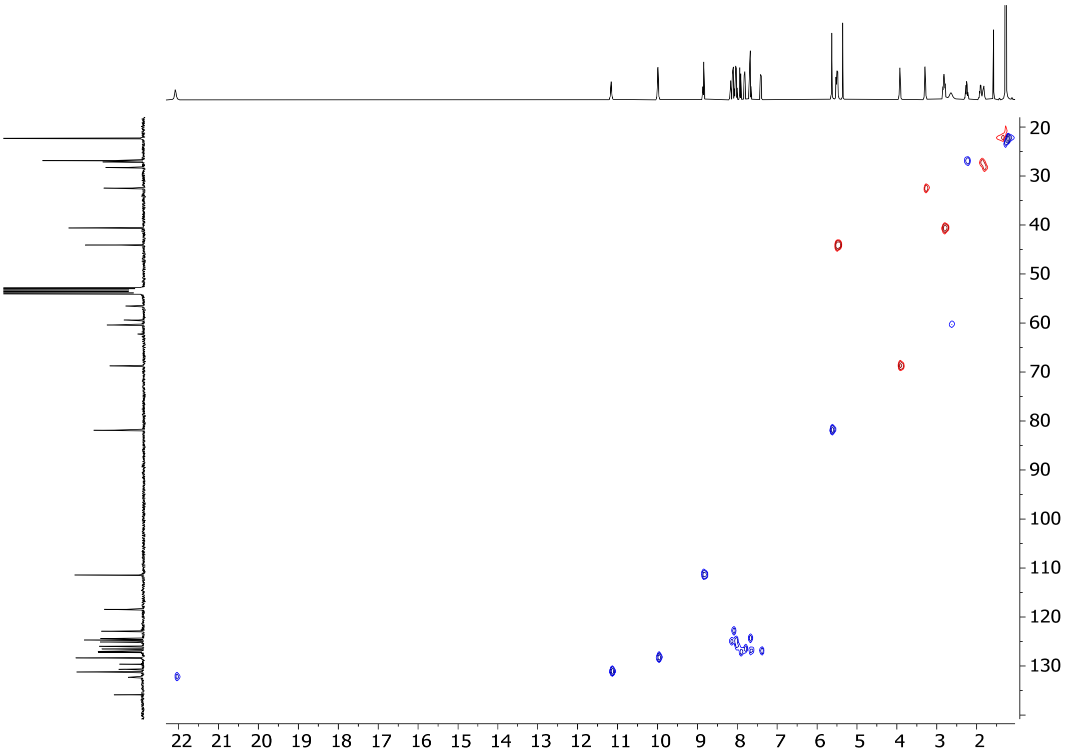

Figure A1-44. HSQC spectrum of [**L2**Eu(hfac)$_3$] in CD_2Cl_2.

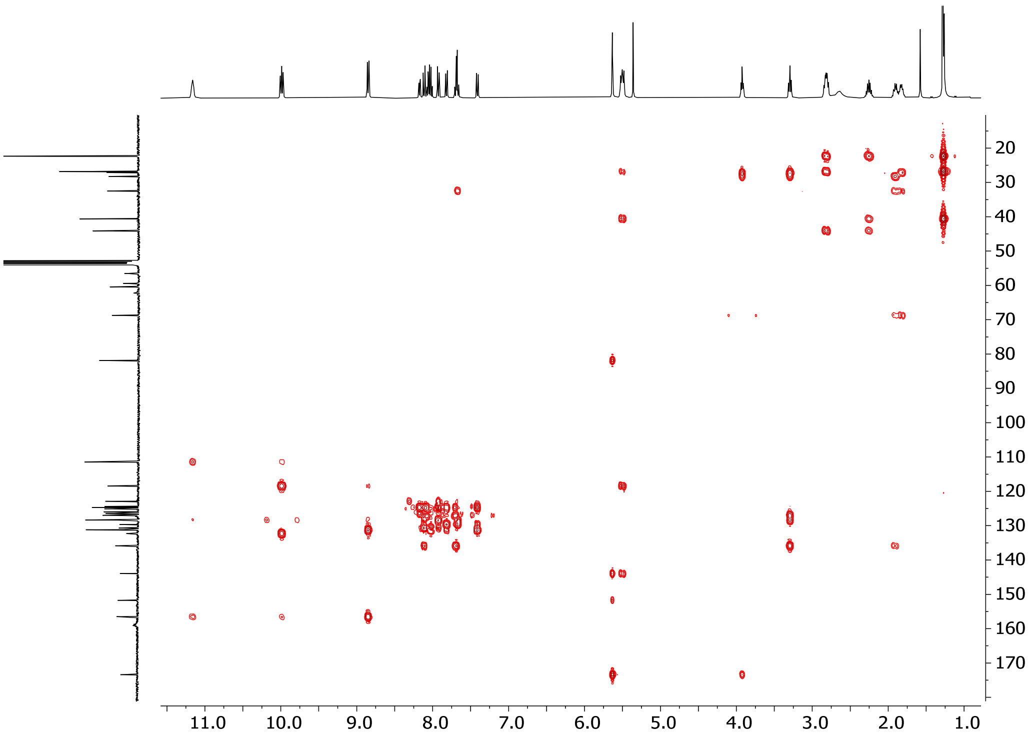

Figure A1-45. HMBC spectrum of [**L2**Eu(hfac)$_3$] in CD_2Cl_2.

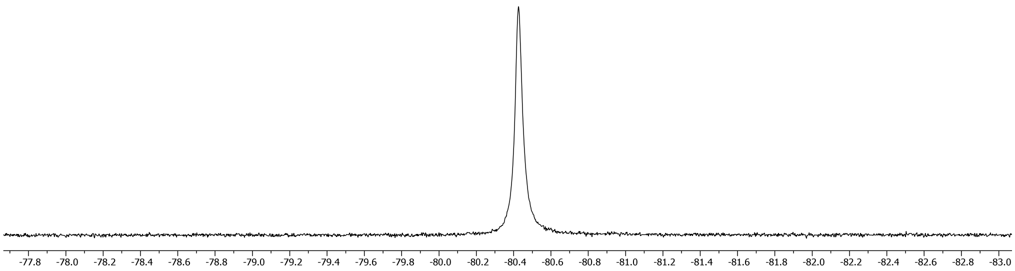

Figure A1-46. ^{19}F NMR spectrum of [**L2**Eu(hfac)$_3$] in CD_2Cl_2.

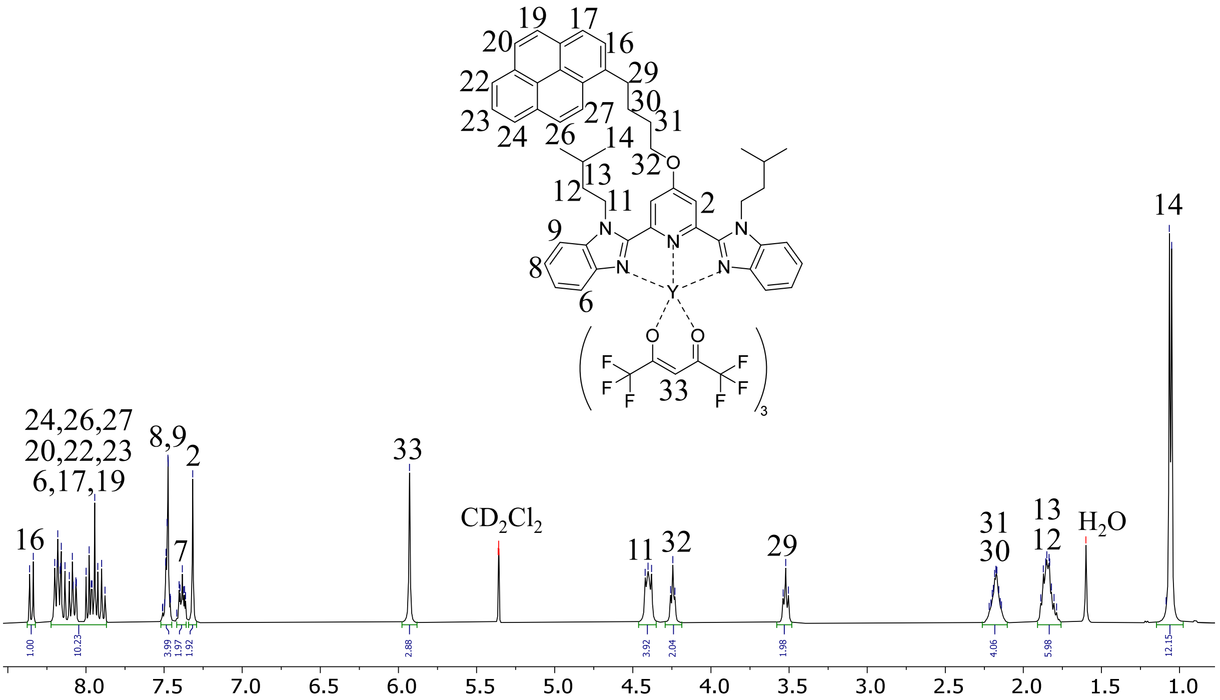

Figure A1-47. ^{1}H NMR spectrum of [**L2**Y(hfac)$_3$] in CD_2Cl_2.

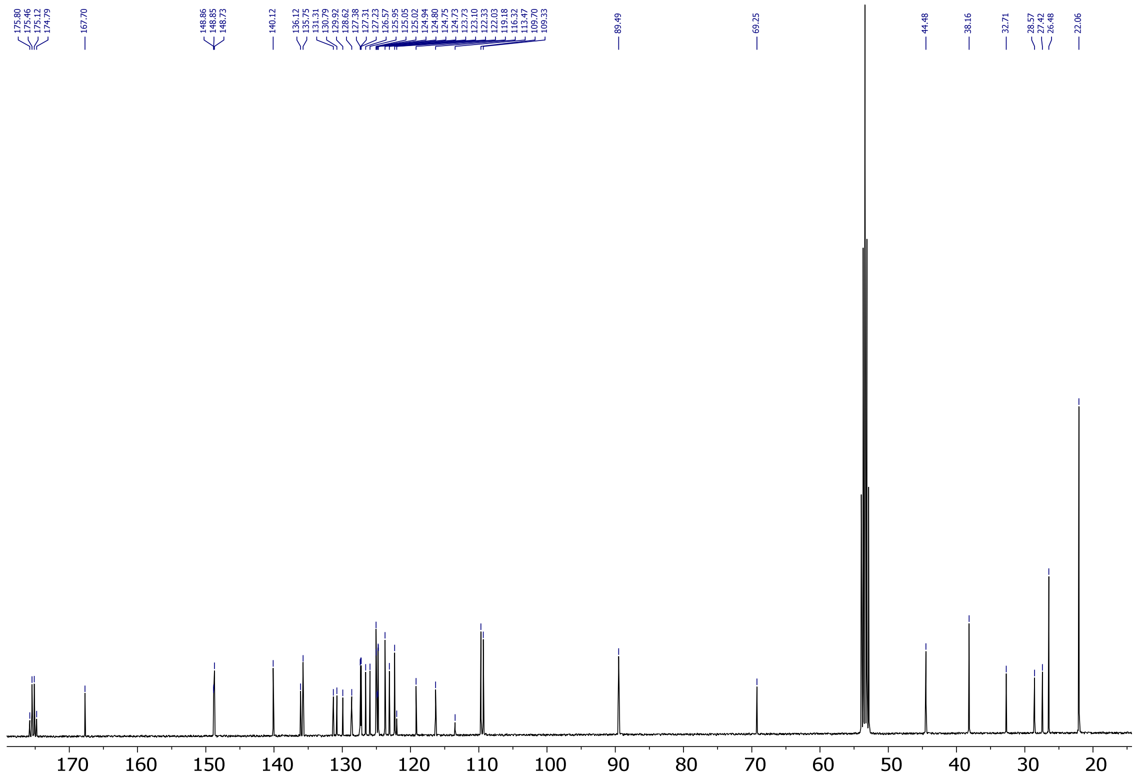

Figure A1-48. ^{13}C NMR spectrum of [**L2**Y(hfac)$_3$] in CD_2Cl_2.

HG510Y-5X10-2M-298K-all.2.fid

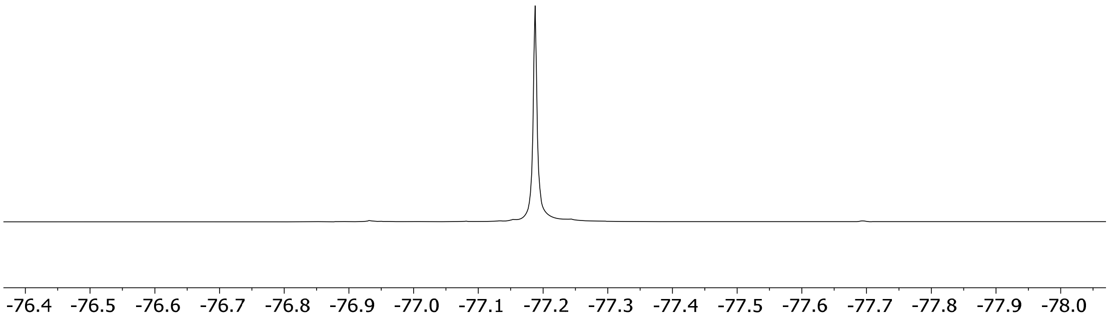

Figure A1-49. ^{19}F NMR spectrum of [**L2**Y(hfac)$_3$] in CD_2Cl_2.

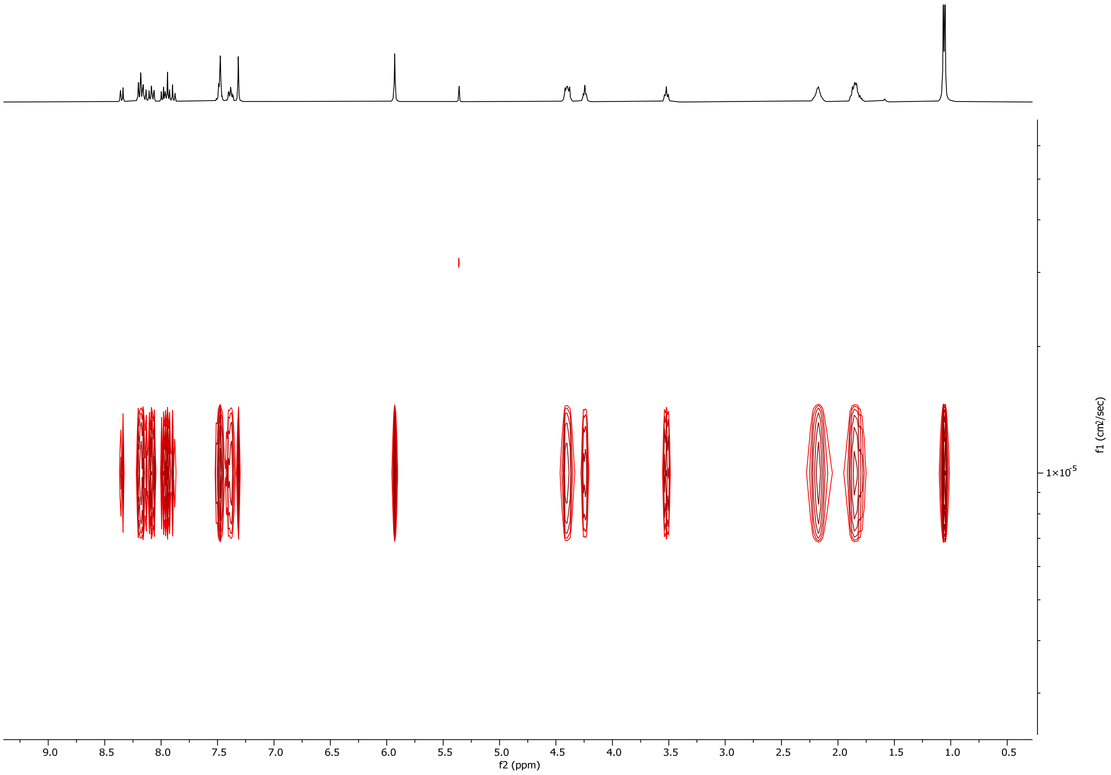

Figure A1-50. DOSY spectrum of [**L2**Y(hfac)$_3$] in CD_2Cl_2.

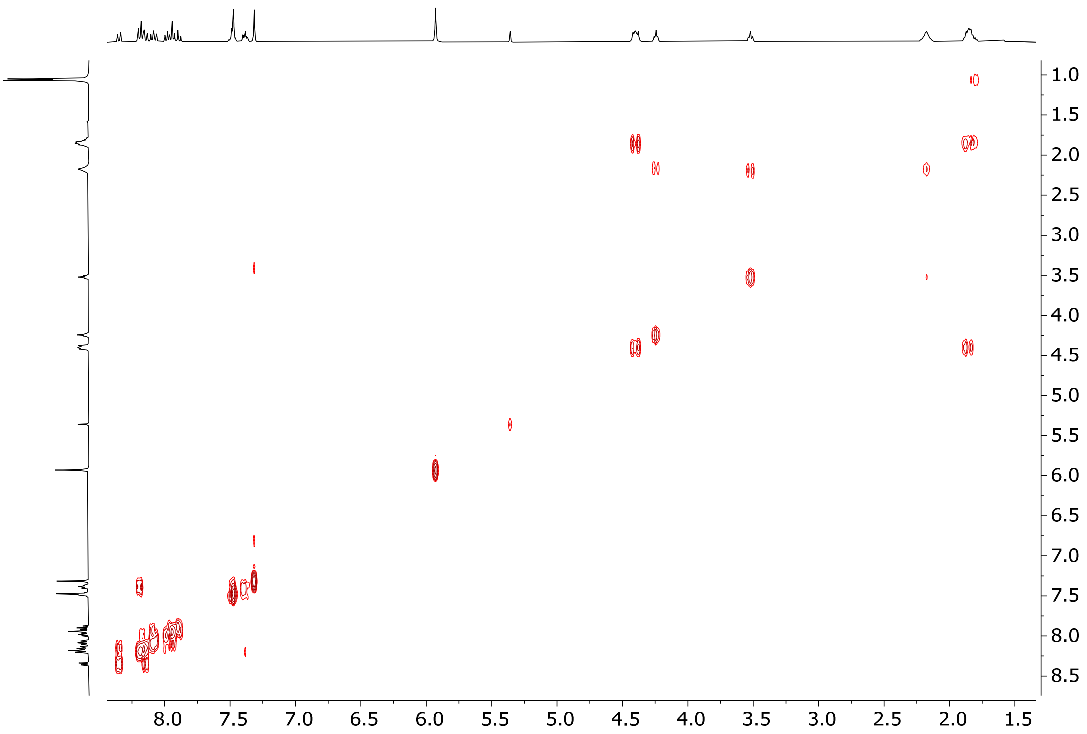

Figure A1-51. COSY spectrum of [**L2**Y(hfac)$_3$] in CD_2Cl_2.

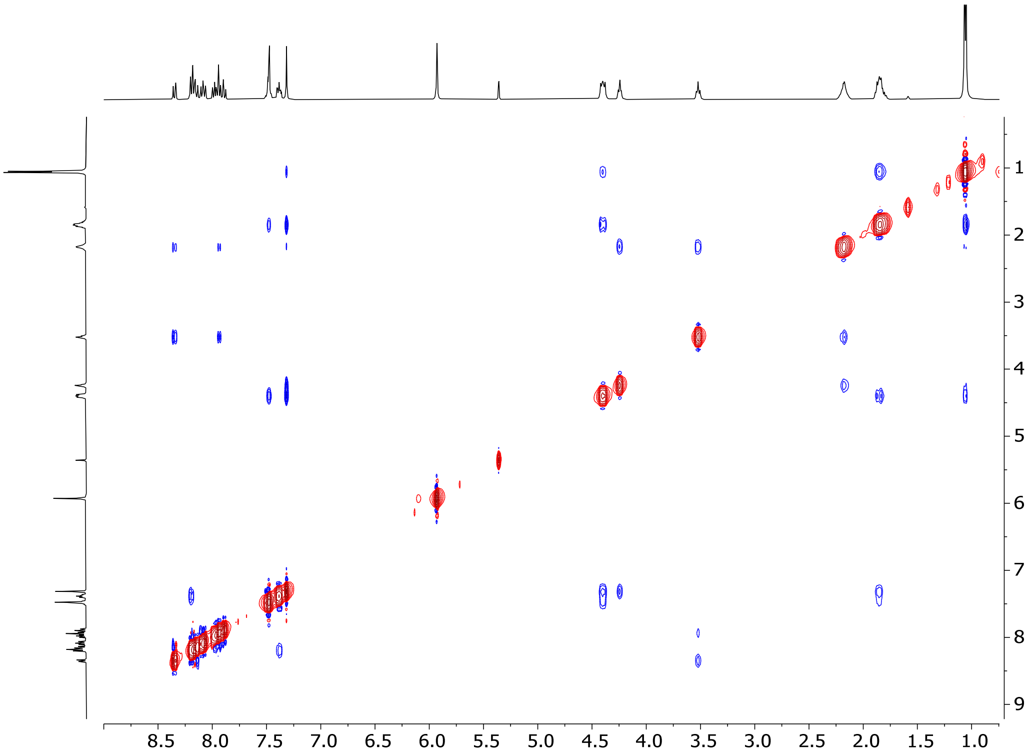

Figure A1-52. NOESY spectrum of [**L2**Y(hfac)$_3$] in CD_2Cl_2.

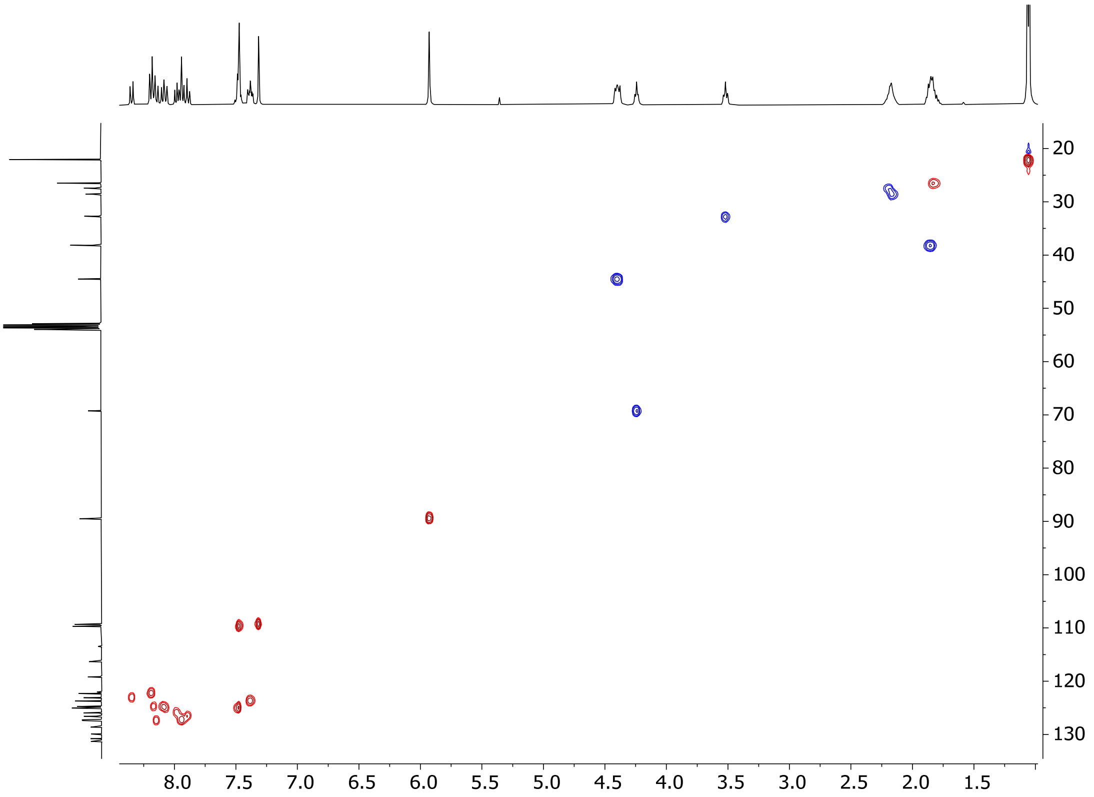

Figure A1-53. HSQC spectrum of [**L2**Y(hfac)$_3$] in CD_2Cl_2.

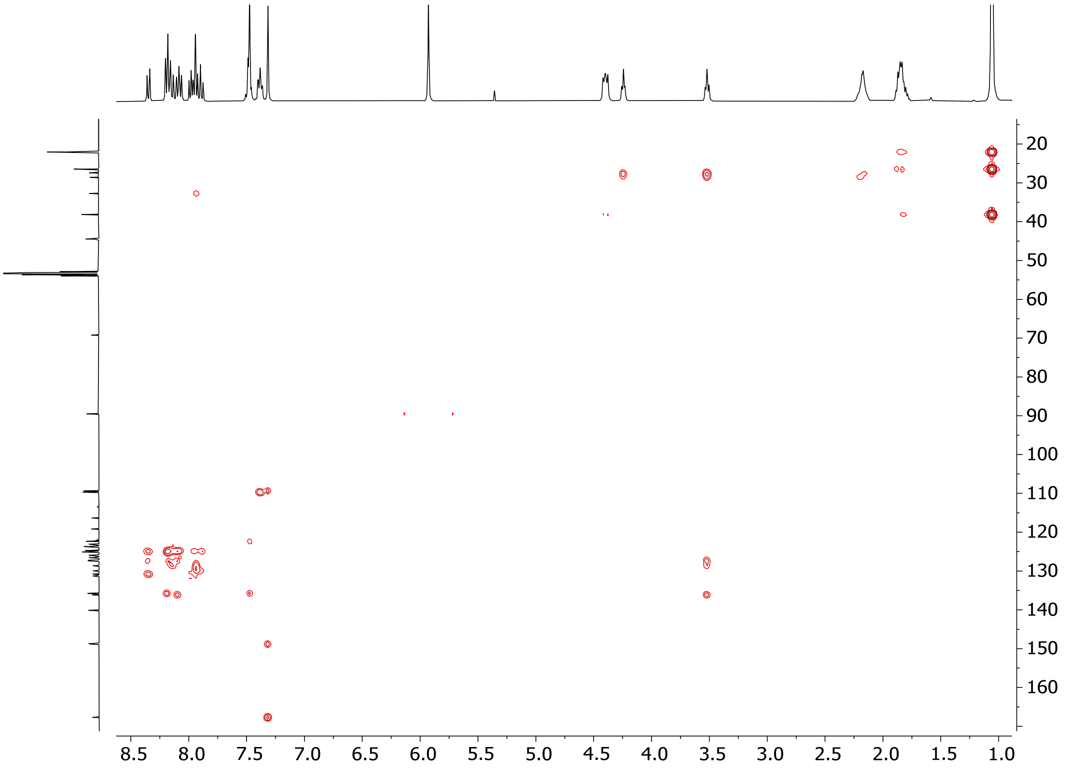

Figure A1-54. HMBC spectrum of [**L2**Y(hfac)$_3$] in CD_2Cl_2.

References

(A1-1) A. Zaïm, H. Nozary, L. Guénée, C. Besnard, J.-F. Lemonnier, S. Petoud, C. Piguet, *Chem. Eur. J.* **2012**, *18*, 7155-7168. DOI: 10.1002/chem.201102827.

(A1-2) G. Zaragoza-Galán, M. A. Fowler, J. Duhamel, R. Rein, N. Solladié, E. Rivera, *Langmuir*, **2012**, *28*, 11195-11205. DOI: 10.1021/la301284v.

(A1-3) P. Froidevaux, J. M. Harrowfield, A. N. Sobolev, *Inorg. Chem.* **2000**, *39*, 4678. DOI: 10.1021/ic000353z.

(A1-4) G. Le-Hoang, L. Guénée, M. Bertrand-Avebe, L. Babel, A. Rosspeintner, C. Piguet, *Inorg. Chem.* **2025**, *64*, 3941–3958. DOI: 10.1021/acs.inorgchem.4c05202.

(A1-5) G. Malandrino, M. Bettinelli, A. Speghini, I. L. Fragalà, *Eur. J. Inorg. Chem.* **2001**, *4*, 1039-1044. DOI: 10.1002/1099-0682(200104)2001:4<1039::AID-EJIC1039>3.0.CO;2-2

(A1-6) G. Malandrino, R. Nigro, I. L. Fragalà, C. Benelli, *Eur. J. Inorg. Chem.* **2004**, *3*, 500-509. DOI: 10.1002/ejic.200300354.

Appendix 2: Crystal structures analysis of ligands and lanthanide complexes

Crystals were mounted on Hampton cryoloops with protection oil. X-ray data collections were performed with a XtaLAB Synergy-S diffractometer equipped with a hybrid pixel array “hypix arc 150” detector (Cu[Kα] radiation).

The structures were solved by using dual-space methods (ShelXT)[A2-1]. Full-matrix least-square refinements on F^2 were performed with SHELXL[A2-1, A2-2] and all other calculations were performed with OLEX2[A2-3] and ORTEP[A2-4] programs.

CCDC 2531606-2531611 contain the supplementary crystallographic data for this paper. These data can be obtained free of charge from The Cambridge Crystallographic Data Centre via www.ccdc.cam.ac.uk/data_request/cif.

Comments on crystal structures:

[L1Eu(hfac)$_3$]:

A hexane solvent molecule is highly disordered in cavities, giving only weak peaks in the electronic density map. Squeeze bypass method was used to refine the solvent free structure. A solvent mask was calculated, and 288 electrons were found in a volume of 960A^3 in 1 void per unit cell. This is consistent with the presence of 1.5[C_6H_{14}] per formula unit which accounts for 300 electrons per unit cell.

[L1Y(hfac)$_3$]:

Solvent molecules (hexane) in cavities were too disordered to be modelled correctly, and "squeeze" bypass program as implemented in Olex2 software was used during the refinement of the solvent-free structure. A solvent mask was calculated, and 440 electrons were found in a volume of 1900A^3 in 1 void per unit cell. This is consistent with the presence of 2.25[C_6H_{14}] per formula unit which accounts for 450 electrons per unit cell.

References

(A2-1) G. M. Sheldrick, *Acta Crystallogr. C: Structural Chemistry* **2015**, *71*, 3-8.

(A2-2) G. M. Sheldrick, *Acta Crystallogr. A* **2008**, *64*, 112-122.

(A2-3) O. V. Dolomanov, L. J. Bourhis, R. J. Gildea, J. A. K. Howard, H. Puschmann, *J. Appl. Crystallogr.* **2009**, *42*, 339-341.

(A2-4) L. Farrugia, *J. Appl. Crystallogr.* **1997**, *30*, 565.

Table A2-1. Summary of crystal data, intensity measurements and structure refinements for **L1**.

CCDC number	2531606	
Empirical formula	$C_{67}H_{61}N_5$	
Chemical formula moiety	$C_{67}H_{61}N_5$	
Formula weight	936.20	
Temperature	120.00(10) K	
Wavelength	1.54184 Å	
Crystal system	Monoclinic	
Space group	P 2_1/c	
Unit cell dimensions	a = 26.0953(2) Å	α = 90°.
	b = 12.63448(11) Å	β = 103.8568(9)°.
	c = 16.11327(12) Å	γ = 90°.
Volume	5157.94(8) $Å^3$	
Z	4	
Density (calculated)	1.206 Mg/m^3	
Absorption coefficient	0.537 mm^{-1}	
F(000)	1992	
Crystal size	0.36 x 0.23 x 0.07 mm^3	
Theta range for data collection	3.489 to 74.250°.	
Index ranges	-31<=h<=32, -15<=k<=15, -16<=l<=20	
Reflections collected	53092	
Independent reflections	10382 [R(int) = 0.0380]	
Completeness to theta = 67.684°	99.9 %	
Absorption correction	Analytical	
Max. and min. transmission	0.967 and 0.877	
Refinement method	Full-matrix least-squares on F^2	
Data / restraints / parameters	10382 / 0 / 653	
Goodness-of-fit on F^2	1.057	
Final R indices [I>2sigma(I)]	R1 = 0.0484, wR2 = 0.1275	
R indices (all data)	R1 = 0.0544, wR2 = 0.1312	
Extinction coefficient	n/a	
Largest diff. peak and hole	0.484 and -0.215 e.$Å^{-3}$	

Table A2-2. Selected least-squares planes data of **L1**.

	Abbreviation	RMSD (Å)	Max deviation (Å) (Atom)
Benzimidazole (1)<br>N1 C7 N2 C6 C1 C3 C2 C4 C5	Bz1	0.006	C7 (0.012)
Benzimidazole (2)<br>C37 N4 C38 C39 C40 C41 C42 C43 N5	Bz2	0.009	N5 (0.015)
Pyridine<br>N3 C36 C35 C34 C33 C32	Py	0.010	C32 (0.014)

Table A2-3. Interplanar angles (°) for **L1**.

Plane	Benzimidazole (1)	Benzimidazole (2)
Pyridine	7.23(4)	49.20(5)
Benzimidazole (1)		55.77(4)

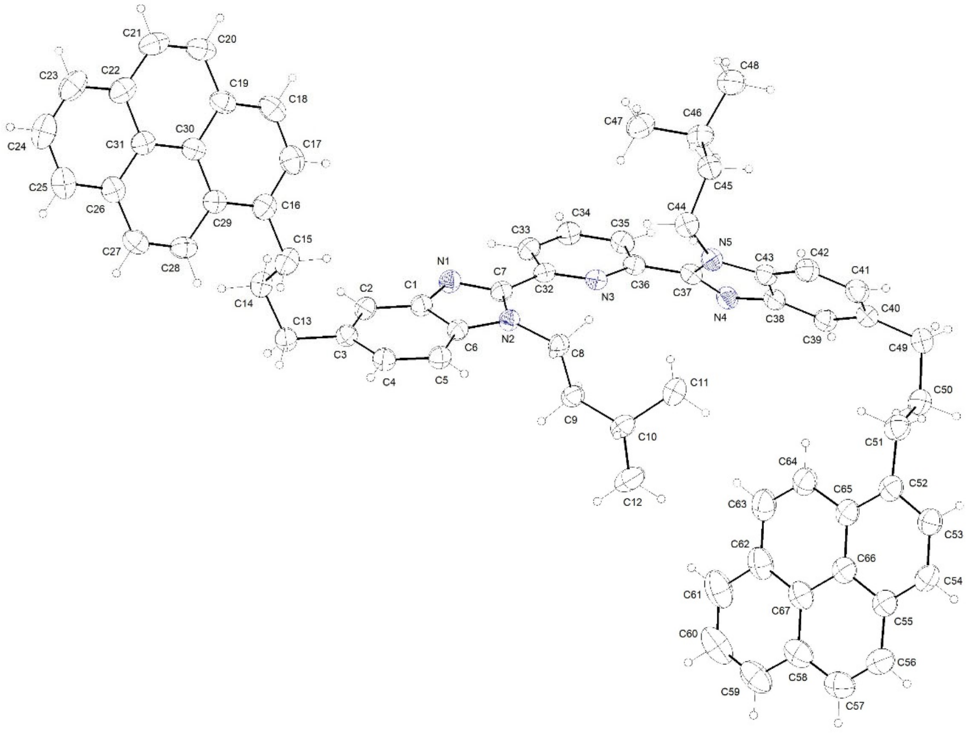

Figure A2-1. Ortep view of **L1** (thermal ellipsoids are drawn at 50% probability) with numbering scheme.

Table A2-4. Summary of crystal data, intensity measurements and structure refinements for **L2**.

CCDC number	2531607	
Empirical formula	$C_{49}H_{49}N_5O$	
Chemical formula moiety	$C_{49}H_{49}N_5O$	
Formula weight	723.93	
Temperature	120.00(10) K	
Wavelength	1.54184 Å	
Crystal system	Monoclinic	
Space group	P 2_1/n	
Unit cell dimensions	a = 19.71021(19) Å	α = 90°.
	b = 17.83878(16) Å	β = 94.1684(8)°.
	c = 22.5695(2) Å	γ = 90°.
Volume	7914.58(13) $Å^3$	
Z (Z')	8 (2)	
Density (calculated)	1.215 Mg/m^3	
Absorption coefficient	0.567 mm^{-1}	
F(000)	3088	
Crystal size	0.21 x 0.1 x 0.04 mm^3	
Theta range for data collection	2.875 to 74.289°.	
Index ranges	-24<=h<=24, -20<=k<=22, -26<=l<=28	
Reflections collected	76881	
Independent reflections	15921 [R(int) = 0.0254]	
Completeness to theta = 67.684°	99.9 %	
Absorption correction	Analytical	
Max. and min. transmission	0.983 and 0.911	
Refinement method	Full-matrix least-squares on F^2	
Data / restraints / parameters	15921 / 0 / 999	
Goodness-of-fit on F^2	1.047	
Final R indices [I>2sigma(I)]	R1 = 0.0466, wR2 = 0.1294	
R indices (all data)	R1 = 0.0584, wR2 = 0.1379	
Extinction coefficient	n/a	
Largest diff. peak and hole	0.457 and -0.224 e.$Å^{-3}$	

Table A2-5. Selected least-squares planes data of **L2**.

	Abbreviation	RMSD (Å)	Max deviation (Å) (Atom)
Benzimidazole (1)<br>C7B N2B C6B C5B C4B C3B C2B C1B N1B	Bz1	0.011	N2B (0.021)
Benzimidazole (2)<br>C18B N4B C19B C20B C21B C22B C23B C24B N5B	Bz2	0.010	N5B (0.015)
Pyridine<br>N3B C17B C16B C15B C14B C13B	Py	0.011	C16B (0.016)
Pyrene<br>C34B C35B C36B C37B C38B C39B C40B C49B C44B C43B C42B C41B C45B C46B C47B C48B	Pr	0.036	C45B (0.066)

Table A2-6. Interplanar angles (°) for **L2**.

Plane	Benzimidazole (1)	Benzimidazole (2)
Pyridine	11.98(4)	25.95(4)
Benzimidazole (1)		34.76(4)

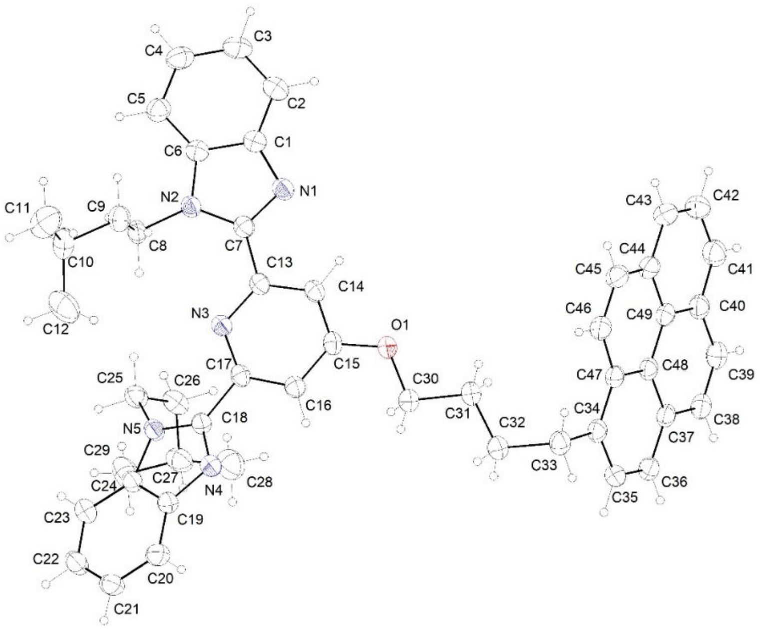

Figure A2-2. Ortep view of molecule A in **L2** (thermal ellipsoids are drawn at 50% probability) with numbering scheme.

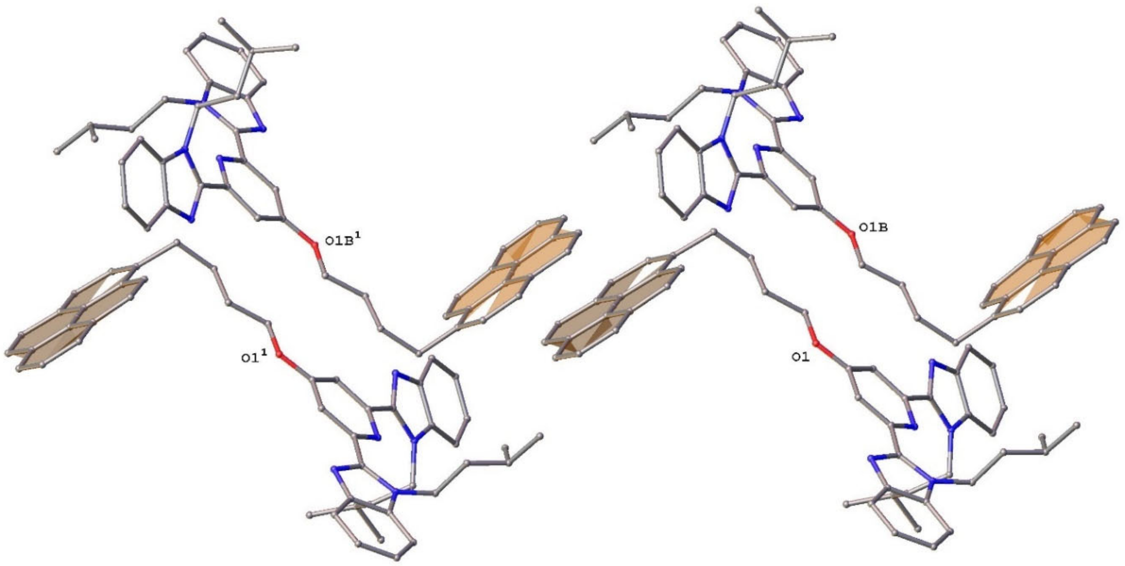

Figure A2-3. Planes and π-stacking analysis in **L2**. Sym. op. 1 (x, -1+y, z).

Intermolecular stacking:

Pr (brown) – PrB^1 (brown):

Angle between planes = 3.47°

Distance Pr to Pr' centroid = 3.35 Å

Table A2-7. Summary of crystal data, intensity measurements and structure refinements for [**L2**Eu(hfac)$_3$].

CCDC number	2531608	
Empirical formula	$C_{64}H_{52}F_{18}N_6O_7Eu$	
Chemical formula moiety	$C_{64}H_{52}F_{18}N_6O_7Eu$	
Formula weight	1497.06	
Temperature	120.00(11) K	
Wavelength	1.54184 Å	
Crystal system	Monoclinic	
Space group	P 2_1/c	
Unit cell dimensions	a = 22.92754(11) Å	α = 90°.
	b = 16.67760(8) Å	β = 97.6632(4)°.
	c = 16.28183(7) Å	γ = 90°.
Volume	6170.18(5) Å^3	
Z	4	
Density (calculated)	1.612 Mg/m^3	
Absorption coefficient	8.281 mm^{-1}	
F(000)	3008	
Crystal size	0.74 x 0.06 x 0.03 mm^3	
Theta range for data collection	3.287 to 76.000°.	
Index ranges	-28<=h<=28, -20<=k<=20, -20<=l<=13	
Reflections collected	112105	
Independent reflections	12629 [R(int) = 0.0360]	
Completeness to theta = 67.684°	100.0 %	
Absorption correction	Analytical	
Max. and min. transmission	0.788 and 0.140	
Refinement method	Full-matrix least-squares on F^2	
Data / restraints / parameters	12629 / 30 / 918	
Goodness-of-fit on F^2	1.043	
Final R indices [I>2sigma(I)]	R1 = 0.0436, wR2 = 0.1068	
R indices (all data)	R1 = 0.0486, wR2 = 0.1096	
Extinction coefficient	n/a	
Largest diff. peak and hole	1.110 and -0.650 e.Å^{-3}	

Table A2-8. Selected least-squares planes data of [**L2**Eu(hfac)$_3$].

	Abbreviation	RMSD (Å)	Max deviation (Å) (Atom)
Benzimidazole (1) N1 C7 N2 C6 C1 C2 C3 C4 C5	Bz1	0.005	C5 (0.009)
Benzimidazole (2) N4 C18 N5 C24 C19 C20 C21 C22 C23	Bz2	0.018	C18 (0.026)
Pyridine N3 C17 C16 C15 C14 C13	Py	0.005	C13 (0.009)
Pyrene C36 C35 C34 C47 C48 C37 C38 C39 C40 C41 C42 C43 C44 C49 C45 C46	Pr	0.031	C34 (0.068)

Table A2-9. Interplanar angles (°) for [**L2**Eu(hfac)$_3$].

Plane	Benzimidazole (1)	Benzimidazole (2)	Hfac1	Hfac2	Hfac3
Pyridine	29.1(1)	10.60(8)	54.49(12)	26.52(9)	65.68(12)
Benzimidazole (1)		38.34(8)	70.74(11)	20.15(10)	36.90(12)
Benzimidazole (2)			45.50(11)	30.53(8)	73.89(10)
Hfac1				51.22(13)	89.43(11)
Hfac2					45.99(13)

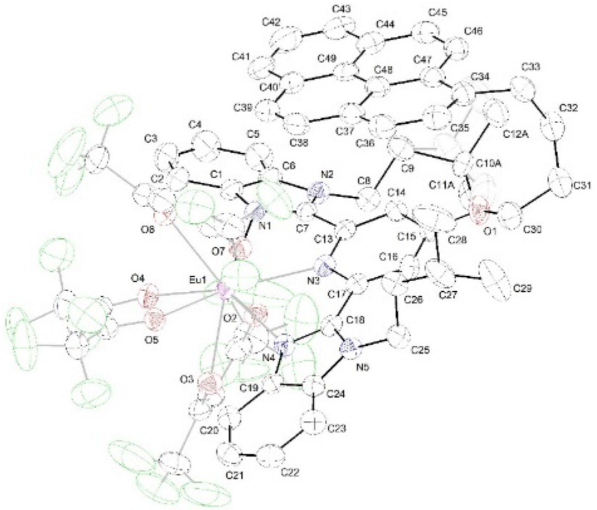

Figure A2-4. ORTEP view of [**L2**Eu(hfac)$_3$] (thermal ellipsoids are drawn at 50% probability level) with numbering scheme.

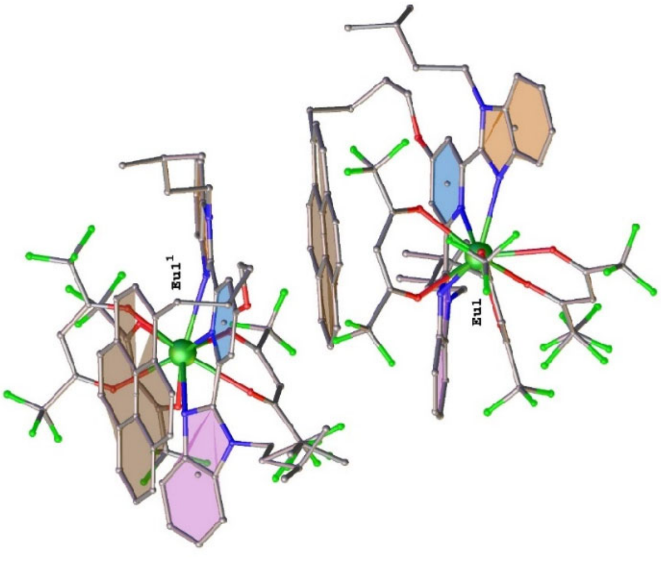

Figure A2-5. Planes and π-π stacking in [**L2**Eu(hfac)$_3$]. Sym. op. ^{1} (x, 3/2-y, ½+z).

Intramolecular Pr(brown)- Py(blue):
Angle between planes = 13.91°
Distance Py to Pr centroid = 3.22 Å
Intramolecular Pr(brown)- Bz(pink):
Angle between planes = 17.58°
Distance Pr to Bz centroid = 3.17 Å

Intermolecular Pr(brown)- Py^1(blue):
Angle between planes = 8.40°
Distance Py to Pr centroid = 3.24 Å
Intermolecular Pr(brown)- Bz^1(orange):
Angle between planes = 5.15°
Distance Pr to Bz centroid = 3.22 Å

Table A2-10. Summary of crystal data, intensity measurements and structure refinements for complex [**L2**Y(hfac)$_3$].

CCDC number	2531609	
Empirical formula	$C_{64}H_{52}N_5O_7F_{18}Y$	
Chemical formula moiety	$C_{64}H_{52}N_5O_7F_{18}Y$	
Formula weight	1433.01	
Temperature	119.99(10) K	
Wavelength	1.54184 Å	
Crystal system	Monoclinic	
Space group	P 2_1/c	
Unit cell dimensions	a = 12.36202(19) Å	α = 90°.
	b = 27.8313(4) Å	β = 91.0352(14)°.
	c = 18.0883(2) Å	γ = 90°.
Volume	6222.30(15) Å^3	
Z	4	
Density (calculated)	1.530 Mg/m^3	
Absorption coefficient	2.317 mm^{-1}	
F(000)	2908	
Crystal size	0.61 x 0.03 x 0.01 mm^3	
Theta range for data collection	2.914 to 75.724°.	
Index ranges	-15<=h<=15, -34<=k<=34, -13<=l<=22	
Reflections collected	59316	
Independent reflections	12529 [R(int) = 0.0368]	
Completeness to theta = 67.684°	99.9 %	
Absorption correction	Analytical	
Max. and min. transmission	0.971 and 0.533	
Refinement method	Full-matrix least-squares on F^2	
Data / restraints / parameters	12529 / 1 / 967	
Goodness-of-fit on F^2	1.029	
Final R indices [I>2sigma(I)]	R1 = 0.0582, wR2 = 0.1475	
R indices (all data)	R1 = 0.0753, wR2 = 0.1573	
Extinction coefficient	n/a	
Largest diff. peak and hole	1.175 and -0.493 e.Å^{-3}	

Table A2-11. Selected least-squares planes data of [**L2**Y(hfac)$_3$].

	Abbreviation	RMSD (Å)	Max deviation (Å) (Atom)
Benzimidazole (1)<br>N1 C7 N2 C6 C1 C2 C3 C4 C5	Bz1	0.025	C7 (0.045)
Benzimidazole (2)<br>C18 N5 C24 C19 N4 C20 C21 C22 C23	Bz2	0.007	N4 (0.013)
Pyridine<br>N3 C17 C16 C15 C14 C13	Py	0.010	C15 (0.014)
Pyrene<br>C34 C47 C46 C45 C48 C35 C36 C37 C38<br>C49 C42 C43 C44 C39 C40 C41	Pr	0.021	C34 (0.043)

Table A2-12. Interplanar angles (°) for [**L2**Y(hfac)$_3$].

Plane	Benzimidazole (1)	Benzimidazole (2)	Hfac1	Hfac2	Hfac3
Pyridine	6.12(13)	25.83(12)	61.02(15)	32.45(12)	72.45(15)
Benzimidazole (1)		23.29(10)	72.41(11)	26.68(11)	47.00(13)
Benzimidazole (2)			57.28(15)	26.35(10)	68.64(13)
Hfac1				47.58(16)	88.62(11)
Hfac2					52.54(16)

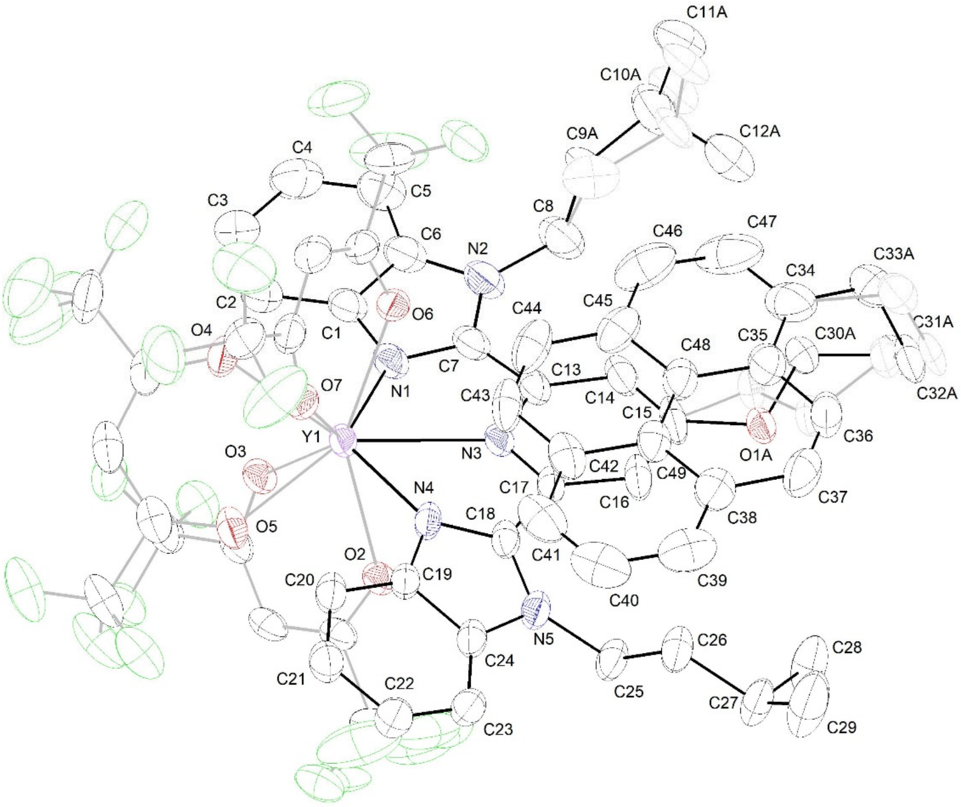

Figure A2-6. ORTEP view of [**L2**Y(hfac)$_3$] (thermal ellipsoids are drawn at 50% probability level) with numbering scheme.

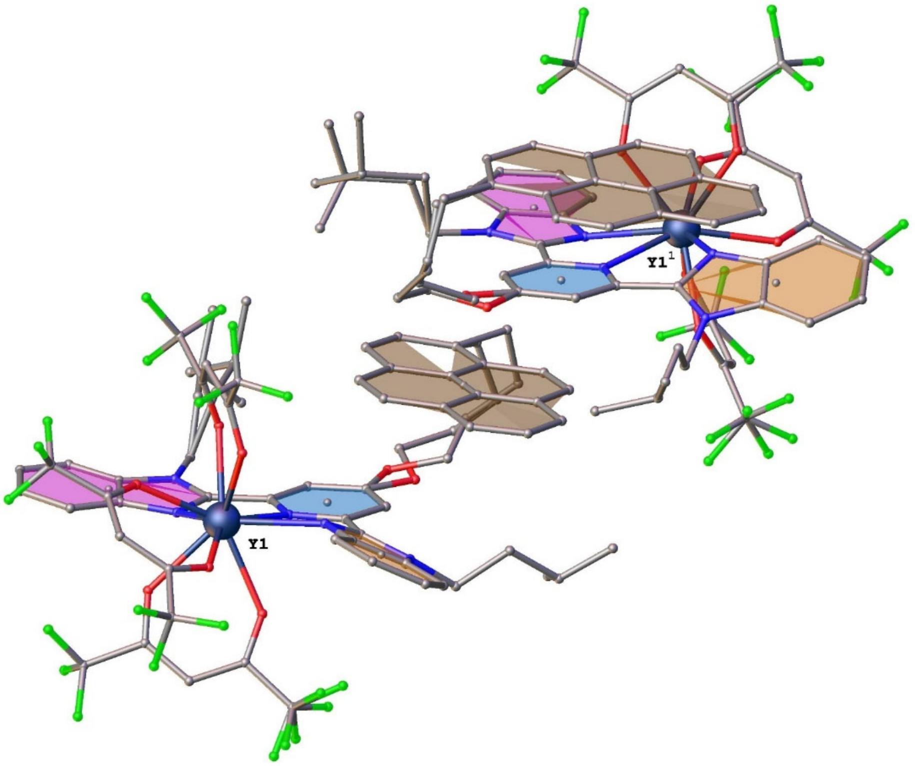

Figure A2-7. Planes and π-π stacking in [**L2**Y(hfac)$_3$]. Sym. Op. ^{1} (x,1/2-y,-1/2+z).

Intramolecular Pr(brown) - Py(blue):

Angle between planes = 5.84°

Distance Py to Pr centroid = 3.30 Å

Intramolecular Pr(brown) - Bz(pink):

Angle between planes = 20.87°

Distance Pr to Bz centroid = 3.18 Å

Intermolecular Pr(brown) – Py^{1}(blue):

Angle between planes = 6.21°

Distance Py to Pr centroid = 3.26 Å

Intermolecular Pr(brown) – Bz^{1}(orange):

Angle between planes = 5.02°

Distance Bz to Pr centroid = 3.26 Å

Table A2-13. Summary of crystal data, intensity measurements and structure refinements for [**L1**Eu(hfac)$_3$]·0.5CH_2Cl_2·1.5C_6H_{14}.

CCDC number	2531610	
Empirical formula	$C_{82}H_{64}N_5O_6F_{18}Eu$	
Chemical formula moiety	$C_{82}H_{64}N_5O_6F_{18}Eu·0.5CH_2Cl_2·1.5C_6H_{14}$	
Formula weight	1881.06	
Temperature	120.00(10) K	
Wavelength	1.54184 Å	
Crystal system	Orthorhombic	
Space group	P *b c n*	
Unit cell dimensions	a = 20.09750(11) Å	α = 90°.
	b = 14.10417(6) Å	β = 90°.
	c = 30.15350(18) Å	γ = 90°.
Volume	8547.27(8) Å^3	
Z (Z')	4 (0.5)	
Density (calculated)	1.462 Mg/m^3	
Absorption coefficient	6.373 mm^{-1}	
F(000)	3840	
Crystal size	0.225 x 0.147 x 0.092 mm^3	
Theta range for data collection	2.931 to 75.967°.	
Index ranges	-24<=h<=24, -9<=k<=17, -37<=l<=35	
Reflections collected	69809	
Independent reflections	8753 [R(int) = 0.0236]	
Completeness to theta = 67.684°	99.9 %	
Absorption correction	Analytical	
Max. and min. transmission	0.609 and 0.372	
Refinement method	Full-matrix least-squares on F^2	
Data / restraints / parameters	8753 / 2 / 521	
Goodness-of-fit on F^2	1.054	
Final R indices [I>2sigma(I)]	R1 = 0.0636, wR2 = 0.1829	
R indices (all data)	R1 = 0.0677, wR2 = 0.1869	
Extinction coefficient	n/a	
Largest diff. peak and hole	1.494 and -0.668 e.Å^{-3}	

Table A2-14. Selected least-squares planes data of complex [**L1**Eu(hfac)$_3$]·0.5CH_2Cl_2·1.5C_6H_{14}.

	Abbreviation	RMSD (Å)	Max deviation (Å) (Atom)
Benzimidazole (1)<br>N1 C4 N2 C10 C5 C6 C7 C8 C9	Bz1	0.012	C6 (0.019)
Benzimidazole (2)<br>N1 C4 N2 C10 C5 C6 C7 C8 C9	Bz1	0.012	C6 (0.019)
Pyridine<br>N3 C3 C2 C1 C2 C3	Py	0.003	C2, C3 (0.003)
Pyrene<br>C19 C32 C31 C30 C29 C28 C27 C26 C25<br>C24 C23 C34 C33 C20 C21 C22	Pr	0.023	C27(0.035)
Hexafluroacetylacetonate (1)<br>O1 C36 C37 C38 O2	Hfac1	0.008	C37 (0.011)
Hexafluroacetylacetonate (2)<br>O3 C41 C42 C41 O3	Hfac2	0.001	C41, C3 (0.001)
Hexafluroacetylacetonate (3)<br>O1 C36 C37 C38 O2	Hfac1	0.008	C37 (0.011)

Table A2-15. Interplanar angles (°) for [**L1**Eu(hfac)$_3$]·0.5CH_2Cl_2·1.5C_6H_{14}.

Plane	Benzimidazole (1)	Benzimidazole (2)	Hfac1	Hfac2	Hfac1
Pyridine	12.92(12)	12.92(12)	76.11(18)	29.7(2)	76.11(18)
Benzimidazole (1)		23.11(16)	80.69(15)	26.41(11)	80.69(15)
Benzimidazole (2)			63.86(16)	26.41(11)	63.86(16)
Hfac1				57.03(14)	88.18(16)
Hfac2					57.03(14)

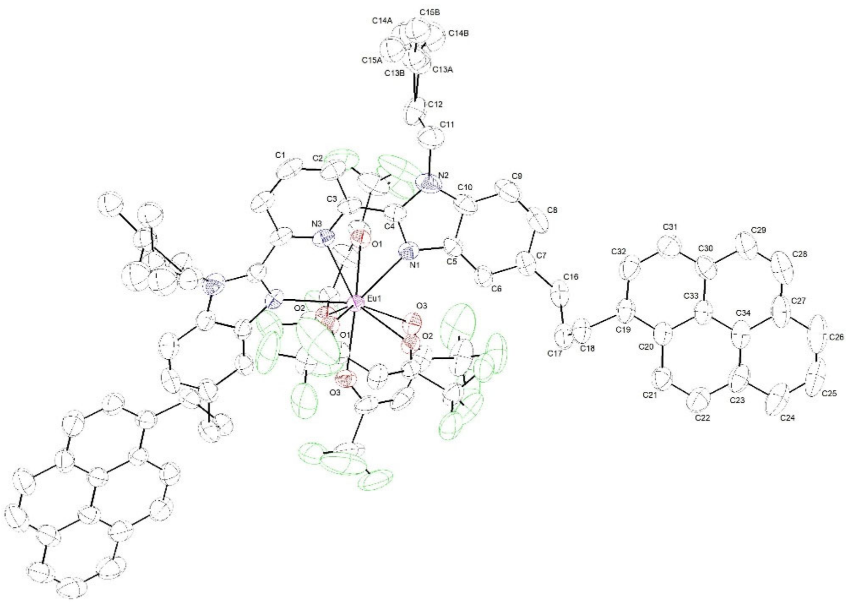

Figure A2-8. ORTEP view of [**L1**Eu(hfac)$_3$] (thermal ellipsoids are drawn at 50% probability level) with numbering scheme of the asymmetric unit. Hydrogen atoms and disordered solvent molecules are omitted for clarity purposes.

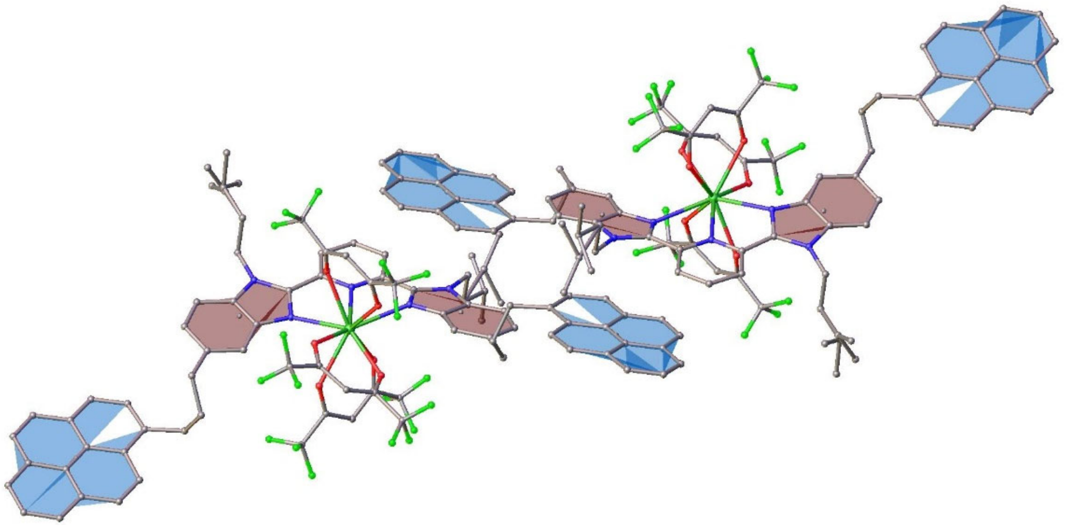

Figure A2-9. Planes and π- π stacking in [**L1**Eu(hfac)$_3$]. Intermolecular interactions between two symmetry-related molecules. (mol1 down left, mol1' (1-x;1-y;1-z) up right).

Bz(brown) – Pr'(blue):

Angle between planes = 1.65°

Distance Bz to Pr' centroid = 3.43 Å

Pr(blue)- Bz'(brown):

Angle between planes = 1.65°

Distance Pyr to Bz' centroid = 3.45 Å

Table A2-16. Summary of crystal data, intensity measurements and structure refinements for [**L1**Y(hfac)$_3$]·2.25C_6H_{14}.

CCDC number	2531611	
Empirical formula	$C_{82}H_{64}N_5O_6F_{18}Y$	
Chemical formula moiety	$C_{82}H_{64}N_5O_6F_{18}Y$·2.25$C_6H_{14}$	
Formula weight	1840.17	
Temperature	119.99(10) K	
Wavelength	1.54184 Å	
Crystal system	Orthorhombic	
Space group	*P b c n*	
Unit cell dimensions	a = 20.11679(14) Å	α = 90°.
	b = 14.05917(8) Å	β = 90°.
	c = 30.12016(14) Å	γ = 90°.
Volume	8518.75(8) Å^3	
Z (Z')	4 (0.5)	
Density (calculated)	1.435 Mg/m^3	
Absorption coefficient	1.813 mm^{-1}	
F(000)	3810	
Crystal size	0.26 x 0.163 x 0.043 mm^3	
Theta range for data collection	2.934 to 75.964°.	
Index ranges	-25<=h<=24, -17<=k<=17, -22<=l<=37	
Reflections collected	75060	
Independent reflections	8673 [R(int) = 0.0301]	
Completeness to theta = 67.684°	99.9 %	
Absorption correction	Analytical	
Max. and min. transmission	0.925 and 0.697	
Refinement method	Full-matrix least-squares on F^2	
Data / restraints / parameters	8673 / 0 / 509	
Goodness-of-fit on F^2	1.084	
Final R indices [I>2sigma(I)]	R1 = 0.0602, wR2 = 0.1795	
R indices (all data)	R1 = 0.0668, wR2 = 0.1850	
Extinction coefficient	n/a	
Largest diff. peak and hole	1.426 and -0.737 e.Å^{-3}	

Table A2-17. Selected least-squares planes data of complex [**L1**Y(hfac)$_3$]·2.25C_6H_{14}.

	Abbreviation	RMSD (Å)	Max deviation (Å) (Atom)
Benzimidazole (1)<br>N1 C4 N2 C10 C5 C6 C7 C8 C9	Bz1	0.013	C6 (0.019)
Benzimidazole (2)<br>N1 C4 N2 C10 C5 C6 C7 C8 C9	Bz1	0.013	C6 (0.019)
Pyridine<br>N3 C3 C2 C1 C2 C3	Py	0.003	C3 (0.004)
Pyrene<br>C19 C30 C31 C32 C33 C34 C27 C26 C25<br>C24 C23 C28 C29 C20 C21 C22	Pr	0.024	C24(0.039)
Hexafluroacetylacetonate (1)<br>O1 C36 C37 C38 O2	Hfac1	0.009	C37 (0.013)
Hexafluroacetylacetonate (2)<br>O3 C41 C42 C41 O3	Hfac2	0.008	C41 (0.009)
Hexafluroacetylacetonate (3)<br>O1 C36 C37 C38 O2	Hfac1	0.009	C37 (0.013)

Table A2-18. Interplanar angles (°) for [**L1**Y(hfac)$_3$]·2.25C_6H_{14}.

Plane	Benzimidazole (1)	Benzimidazole (2)	Hfac1	Hfac2	Hfac1
Pyridine	12.12(8)	12.12(8)	75.61(13)	31.18(14)	75.61(13)
Benzimidazole (1)		21.61(11)	79.76(11)	27.69(9)	79.76(11)
Benzimidazole (2)			64.11(12)	27.69(9)	64.11(12)
Hfac1				55.64(10)	87.34(12)
Hfac2					55.64(10)

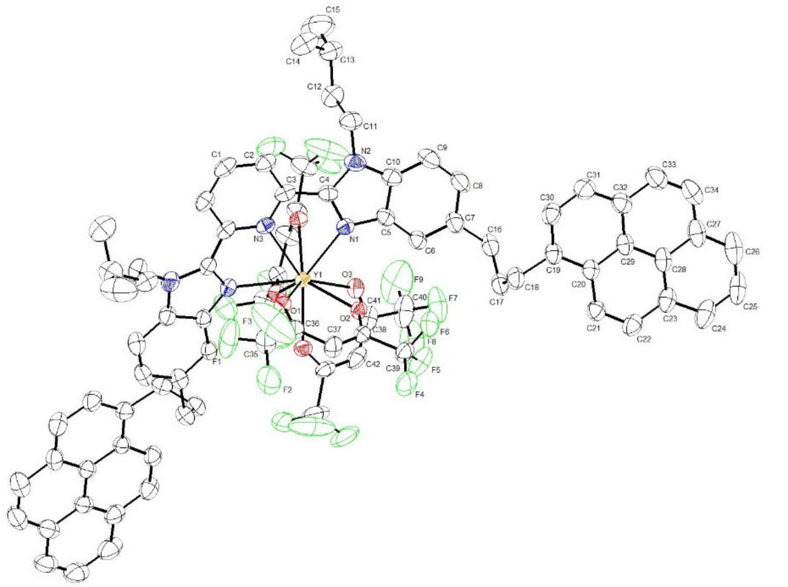

Figure A2-10. Ortep view of [**L1**Y(hfac)$_3$] with numbering scheme of the asymmetric unit (thermal ellipsoids are drawn at 50% probability level). Hydrogen atoms are omitted for clarity purposes.

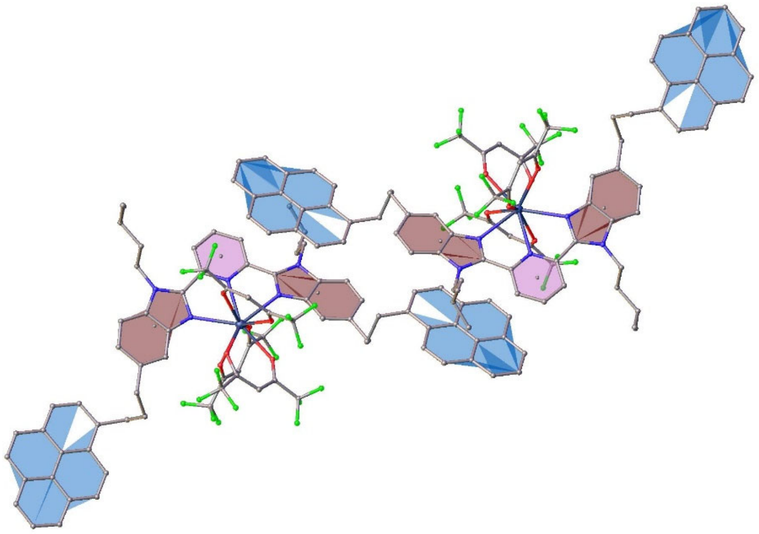

Figure A2-11. π-π Stacking in [**L1**Y(hfac)$_3$]. Intermolecular interaction between two symmetry-related molecules. (mol1 down left, mol1' (1-x;1-y;1-z) up right). Pyridine (Py=pink), Bz (brown), pyrene (Pyr=blue).

Bz(brown) – Pr'(blue):
Angle between planes = 3.09°
Distance Bz to Pyr' centroid = 3.40 A

Py(pink) – Pr'(blue):
Angle between planes = 15.03°
Distance Py to Pyr' centroid = 3.01 A

Pr(blue)- Bz'(brown):
Angle between planes = 3.09°
Distance Pyr to Bz' centroid = 3.47 A

Pr(blue) – Py'(pink):
Angle between planes = 15.03°
Distance Pyr to Py' centroid = 3.55 A

Appendix 3: The aggregation equilibrium constant K_{Dimer} of 1, L1 and [L1Y(hfac)3] in dichloromethane solution.

Table A3-1. The aggregation equilibrium constant K_{Dimer} and the chemical shift $δ_D$ of pure dimer obtained from the titrations of **1**, **L1**, and [**L1**Y(hfac)3] at various concentrations.

	**1**	**L1**	[**L1**Y(hfac)3]
K_{Dimer} /M^{-1}	3.6	3.0	17.4
$δ_D$ /ppm	8.3260	7.9735	7.9920

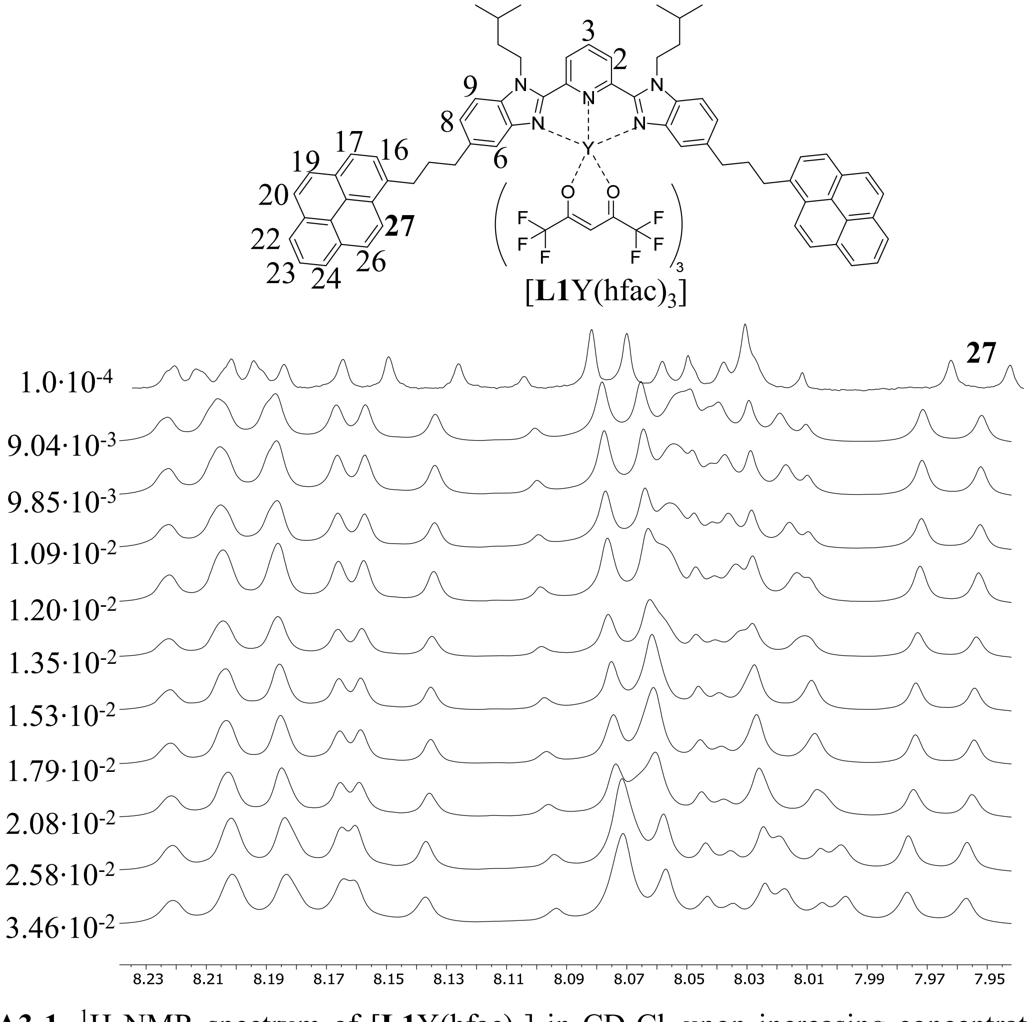

Figure A3-1. ^1H NMR spectrum of [**L1**Y(hfac)3] in CD_2Cl_2 upon increasing concentration for determining dimerization constant.

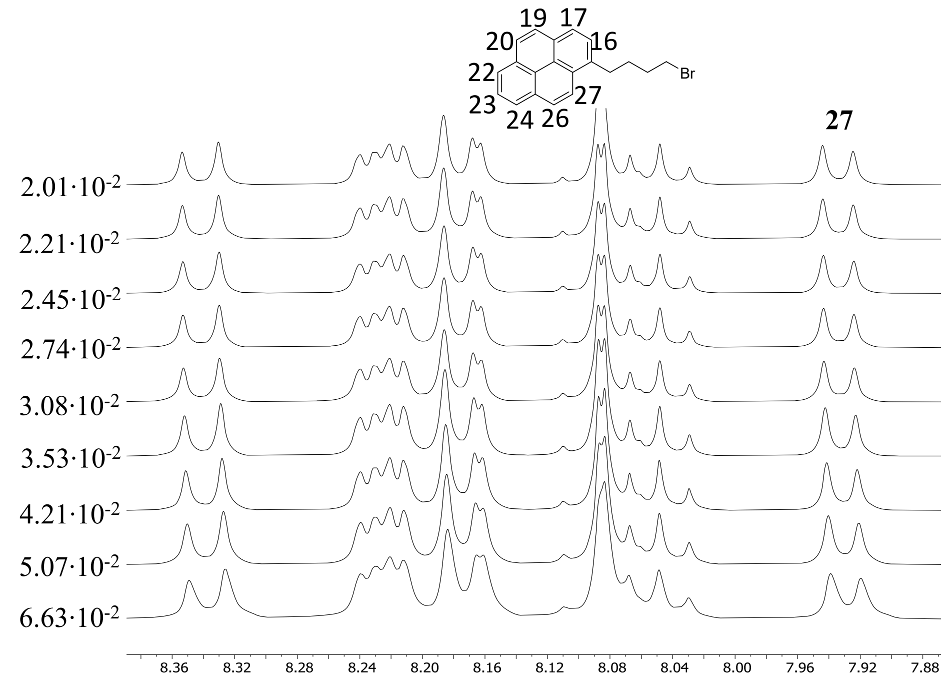

Figure A3-2. ^{1}H NMR spectrum of **1** in CD_2Cl_2 upon increasing concentration for determining dimerization constant.

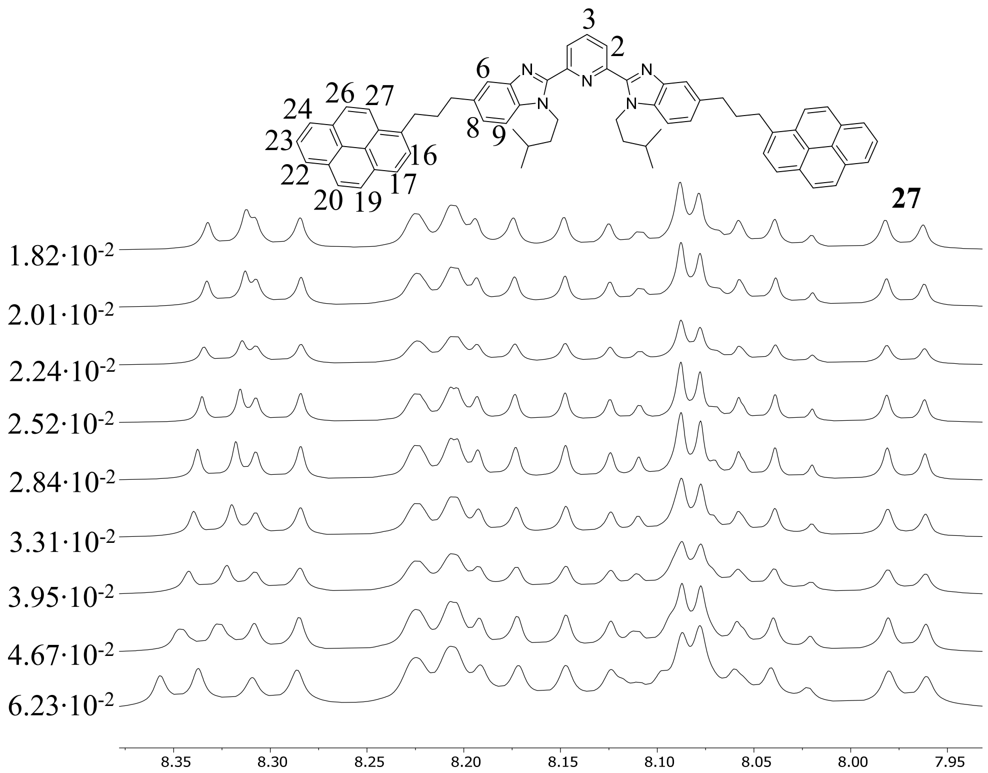

Figure A3-3. ^{1}H NMR spectrum of **L1** in CD_2Cl_2 upon increasing concentration for determining dimerization constant.

Appendix 4: ^{1}H NMR titration of [L1Y(hfac)$_3$] host and aromatic compound guest

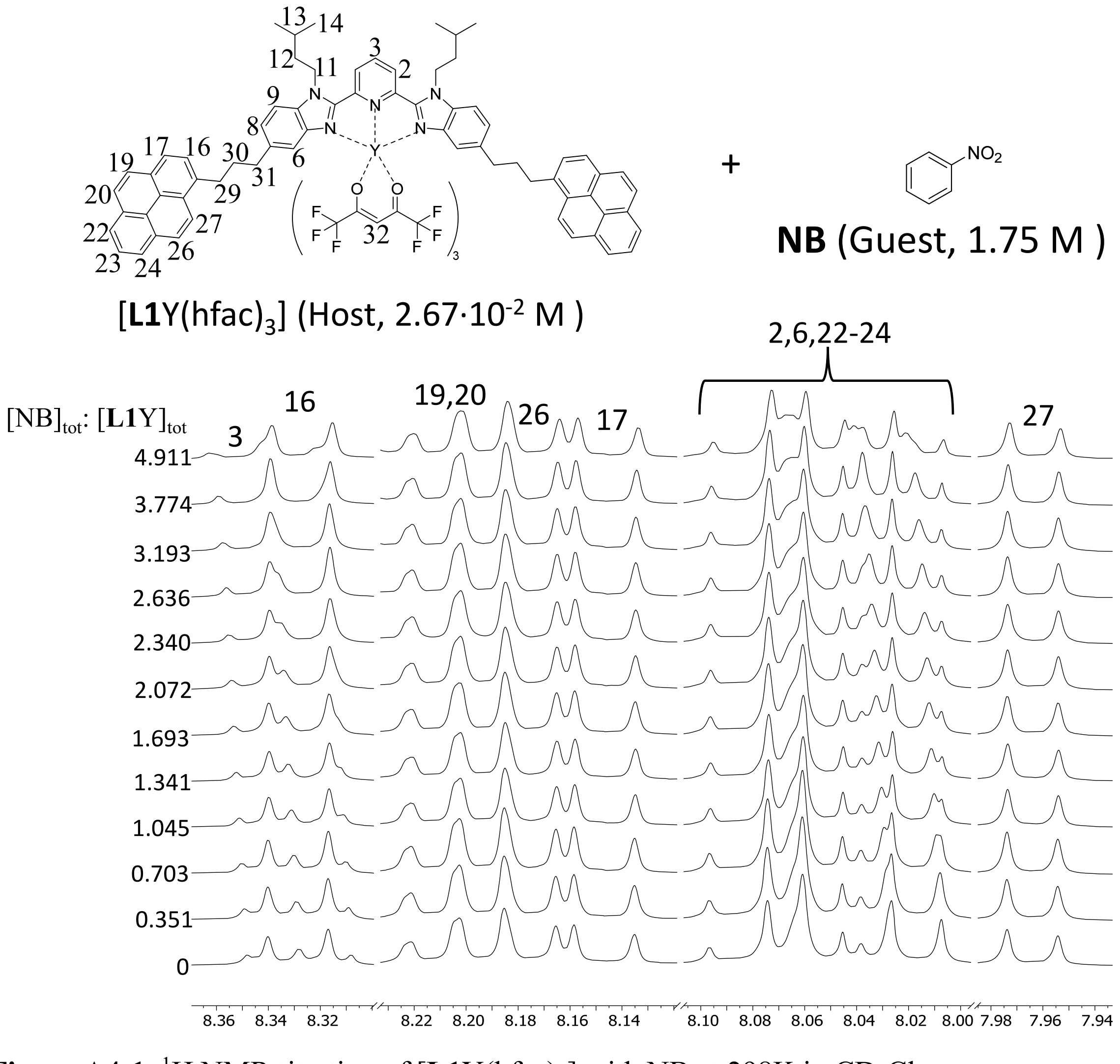

Figure A4-1. ^{1}H NMR titration of [**L1**Y(hfac)$_3$] with NB at 298K in CD_2Cl_2

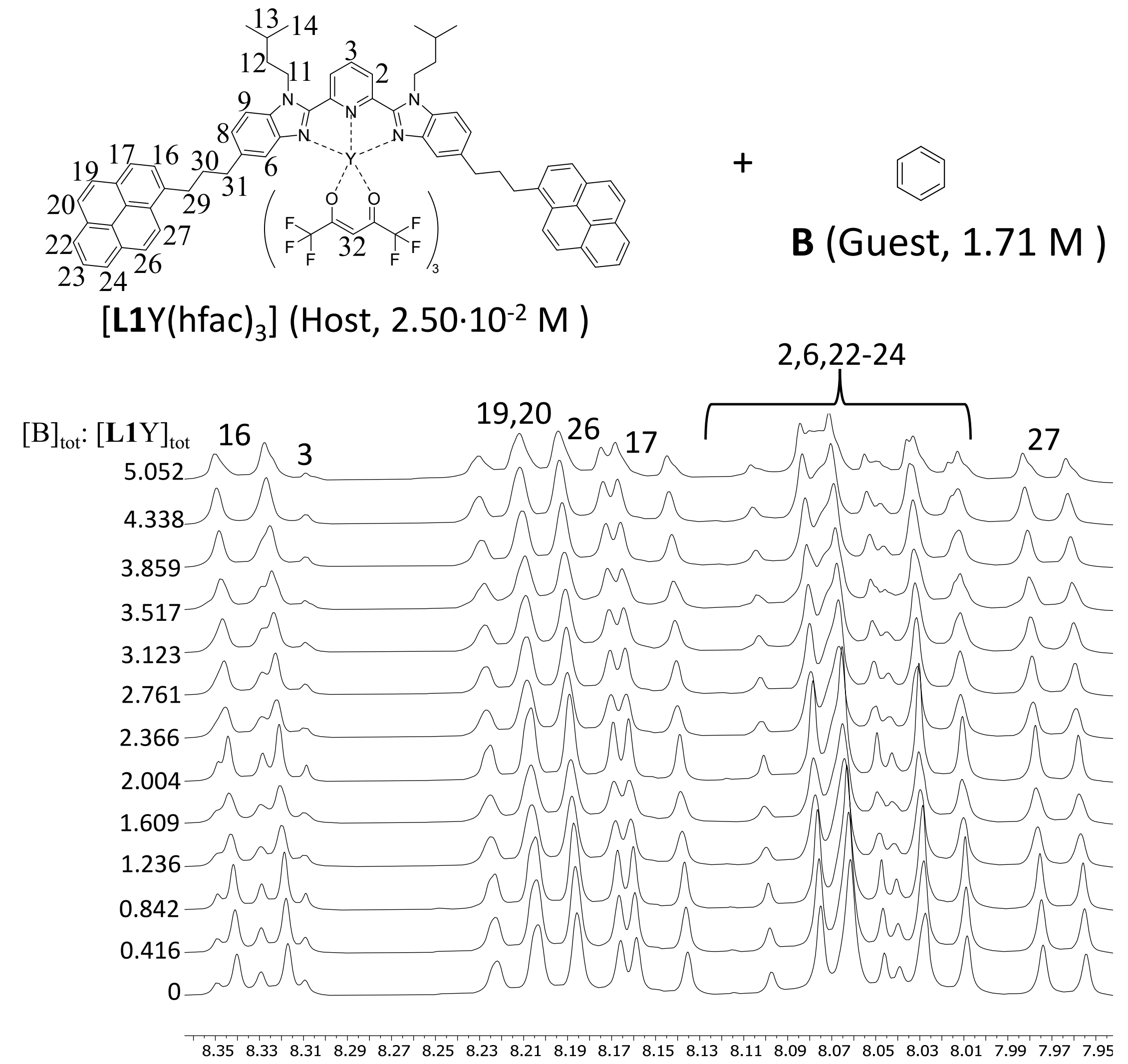

Figure A4-2. ^{1}H NMR titration of [**L1**Y(hfac)$_3$] with B at 298K in CD_2Cl_2

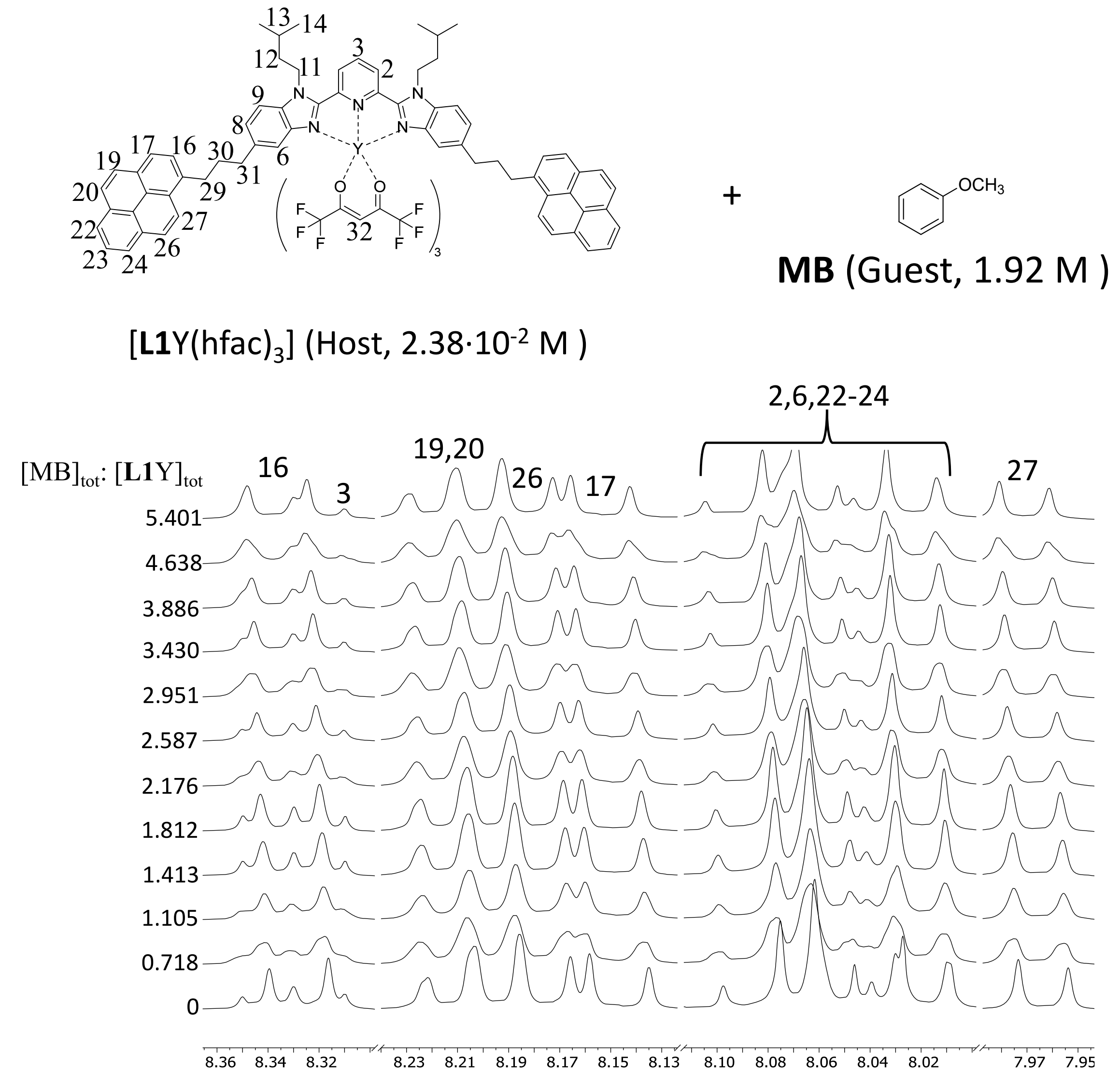

Figure A4-3. ^{1}H NMR titration of [**L1**Y(hfac)$_3$] with MB at 298K in CD_2Cl_2

Appendix 5: Thermodynamic properties of L1 receptor with [Y(hfac)3] guest in dichloromethane.

The binding isotherm rebuilt from obtained stability constant $\beta_{1,1,\mathrm{cond}}^{\mathbf{L1},\mathrm{Y}}$ (dashed-red traces in Figure 7 in the main text) differs from the experimental curve (black diamonds in Figure 7 in the main text) due to the variation in the activity coefficients γ_i occurring in non-ideal organic solutions according to eq (A5-1),[A5-1] where c^{θ} = 1 M stands for the concentration of the reference state.

$$\beta_{1,1,\mathrm{cond}}^{\mathbf{L1},\mathrm{Y}} = \frac{a_{\mathbf{L1}\mathrm{Y}}^{\mathrm{eq}}}{a_{\mathrm{Y}}^{\mathrm{eq}} a_{\mathbf{L1}}^{\mathrm{eq}}} = \frac{\gamma_{\mathbf{L1}\mathrm{Y}}}{\gamma_{\mathrm{Y}}\gamma_{\mathbf{L1}}} \cdot \frac{\left(c_{\mathbf{L1}\mathrm{Y}}^{\mathrm{eq}}/c^{\theta}\right)}{\left(c_{\mathrm{Y}}^{\mathrm{eq}}/c^{\theta}\right)\left(c_{\mathbf{L1}}^{\mathrm{eq}}/c^{\theta}\right)} = \frac{\gamma_{\mathbf{L1}\mathrm{Y}}}{\gamma_{\mathrm{Y}}\gamma_{\mathbf{L1}}} \cdot \left(\frac{|\mathbf{L1}\mathrm{Y}|}{|\mathrm{Y}||\mathbf{L1}|}\right) \cdot c^{\theta}$$

$$= \frac{\gamma_{\mathbf{L1}\mathrm{Y}}}{\gamma_{\mathrm{Y}} \cdot \gamma_{\mathbf{L1}}} \cdot Q_{1,1,\mathrm{cond}}^{\mathbf{L1},\mathrm{Y}} \cdot c^{\theta} \qquad \text{(A5-1)}$$

Eggers and coworkers proposed eq (A5-2) to catch these variations during the host-guest interactions,[A5-2,A5-3] where the experimental quotients of reaction $Q_{1,1,\mathrm{cond}}^{\mathbf{L1},\mathrm{Y}} = \left(\frac{|\mathbf{L1}\mathrm{Y}|}{|\mathrm{Y}||\mathbf{L1}|}\right)$ can be easily estimated at each point of ^{1}H RMN titration.

$$-RT\ln(Q_{1,1,\mathrm{cond}}^{\mathbf{L1},\mathrm{Y}}) = -RT\ln(\beta_{1,1,\mathrm{cond}}^{\mathbf{L1},\mathrm{Y},\infty}) + \Delta G_{1,1,\mathrm{cond}}^{\mathbf{L1},\mathrm{Y},\mathrm{S}} \cdot (|\mathbf{L1}\mathrm{Y}|/c^{\theta}) \qquad \text{(A5-2)}$$

The plots of $-RT\ln(Q_{1,1,\mathrm{cond}}^{\mathbf{L1},\mathrm{Y}})$ versus the concentration of formed final complex $|\mathbf{L1}\mathrm{Y}|$ are close to linear (Fig. A5-1) and provide free energy changes at infinite dilution $\Delta G_{1,1,\mathrm{cond}}^{\mathbf{L1},\mathrm{Y},\infty} = -RT\ln(\beta_{1,1,\mathrm{cond}}^{\mathbf{L1},\mathrm{Y},\infty})$ and solvation free energy changes $\Delta G_{1,1,\mathrm{cond}}^{\mathbf{L1},\mathrm{Y},\mathrm{S}}$ (Table A5-1), from which satisfying occupancy factors $\theta_{\mathbf{L}k}^{\mathrm{Eu}}$ could be rebuilt with eq (A5-3) (green dashed trace in Figure 7 in the main text).

$$\theta_{\mathbf{L1}}^{\mathrm{Y}} = \frac{Q_{1,1,\mathrm{cond}}^{\mathrm{Y},\mathbf{L1}}|\mathrm{Y}|}{1 + Q_{1,1,\mathrm{cond}}^{\mathrm{Y},\mathbf{L1}}|\mathrm{Y}|} = \frac{|\mathbf{L1}\mathrm{Y}|}{|\mathbf{L1}|_{\mathrm{tot}}} = \frac{|\mathrm{Y}|_{\mathrm{tot}} - |\mathrm{Y}|}{|\mathbf{L1}|_{\mathrm{tot}}}$$

$$= \frac{\exp\left[-\left(\Delta G_{1,1,\mathrm{cond}}^{\mathrm{Y},\mathbf{L1},\infty} + \left(|\mathrm{Y}|_{\mathrm{tot}} - |\mathrm{Y}|\right) \cdot \Delta G_{1,1,\mathrm{cond}}^{\mathrm{Y},\mathbf{L1},\mathrm{S}}\right)\Big/RT\right] \cdot |\mathrm{Y}|}{1 + \left\{\exp\left[-\left(\Delta G_{1,1,\mathrm{cond}}^{\mathrm{Y},\mathbf{L1},\infty} + \left(|\mathrm{Y}|_{\mathrm{tot}} - |\mathrm{Y}|\right) \cdot \Delta G_{1,1,\mathrm{cond}}^{\mathrm{Y},\mathbf{L1},\mathrm{S}}\right)\Big/RT\right] \cdot |\mathrm{Y}|\right\}} \qquad \text{(A5-3)}$$

Table A5-1. Associated free energies $\Delta G_{1,1,\mathrm{cond}}^{\mathbf{L1},\mathrm{Y}} = -RT\ln(\beta_{1,1,\mathrm{cond}}^{\mathbf{L1},\mathrm{Y}})$, $\Delta G_{1,1,\mathrm{cond}}^{\mathbf{L1},\mathrm{Y},\infty}$ and $\Delta G_{1,1,\mathrm{cond}}^{\mathbf{L1},\mathrm{Y,S}}$ determined for the titration of ligand **L1** with [**dig**Y(hfac)3] in CD_2Cl_2 + 0.14 M diglyme at 293 K.

$\Delta G_{1,1,\mathrm{cond}}^{\mathbf{L1},\mathrm{Y}}$ /kJ·mol^{-1}	-18.7(4)
$\Delta G_{1,1,\mathrm{cond}}^{\mathbf{L1},\mathrm{Y},\infty}$ /kJ·mol^{-1}	-15.2(4)
$\Delta G_{1,1,\mathrm{cond}}^{\mathbf{L1},\mathrm{Y,S}}$ /kJ·mol^{-1}	-3.4(3)·10^3

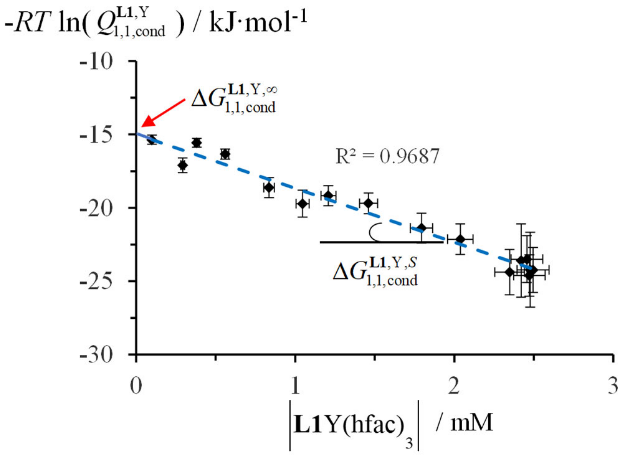

Figure A5-1 Plots of $-RT\ln(Q_{1,1,\mathrm{cond}}^{\mathbf{L1},\mathrm{Y}})$ as a function of $|\mathbf{L1}\mathrm{Y}|$ for the titration of **L1** with [**dig**Y(hfac)3] in CD_2Cl_2 + 0.14 M diglyme at 293K.

References

(A5-1) Baudet, K.; Kale, V.; Mirzakhani, M.; Babel, L.; Naseri, S.; Besnard, C.; Nozary, H.; Piguet, C. Neutral Heteroleptic Lanthanide Complexes for Unravelling Host-Guest Assemblies in Organic Solvents: The Law of Mass Action Revisited. *Inorg. Chem.* **2020**, *59*, 62-75. DOI: 10.1021/acs.inorgchem.9b00755.

(A5-2) Castellano, B. M.; Eggers, D. K. Experimental Support for a Desolvation Energy Term in Governing Equations for Binding Equilibria. *J. Phys. Chem. B* **2013**, **117**, 8180-8188. DOI: 10.1021/jp402632a.

(A5-3) Eggers, D. K.; Le, J. M.; Nahm, N. T.; Pham, D. N.; Castellano, B. M. Dual Effect of Secondary Solutes on Binding Equilibria: Contributions from Solute-Reactant Interactions and Solute-Water Interactions. *ACS Omega* **2024**, *48*, 928-934. DOI: 10.1021/acsomega.3c09329.

Appendix 6: Photophysical parameters of L*k* ligands and their lanthanide complexes

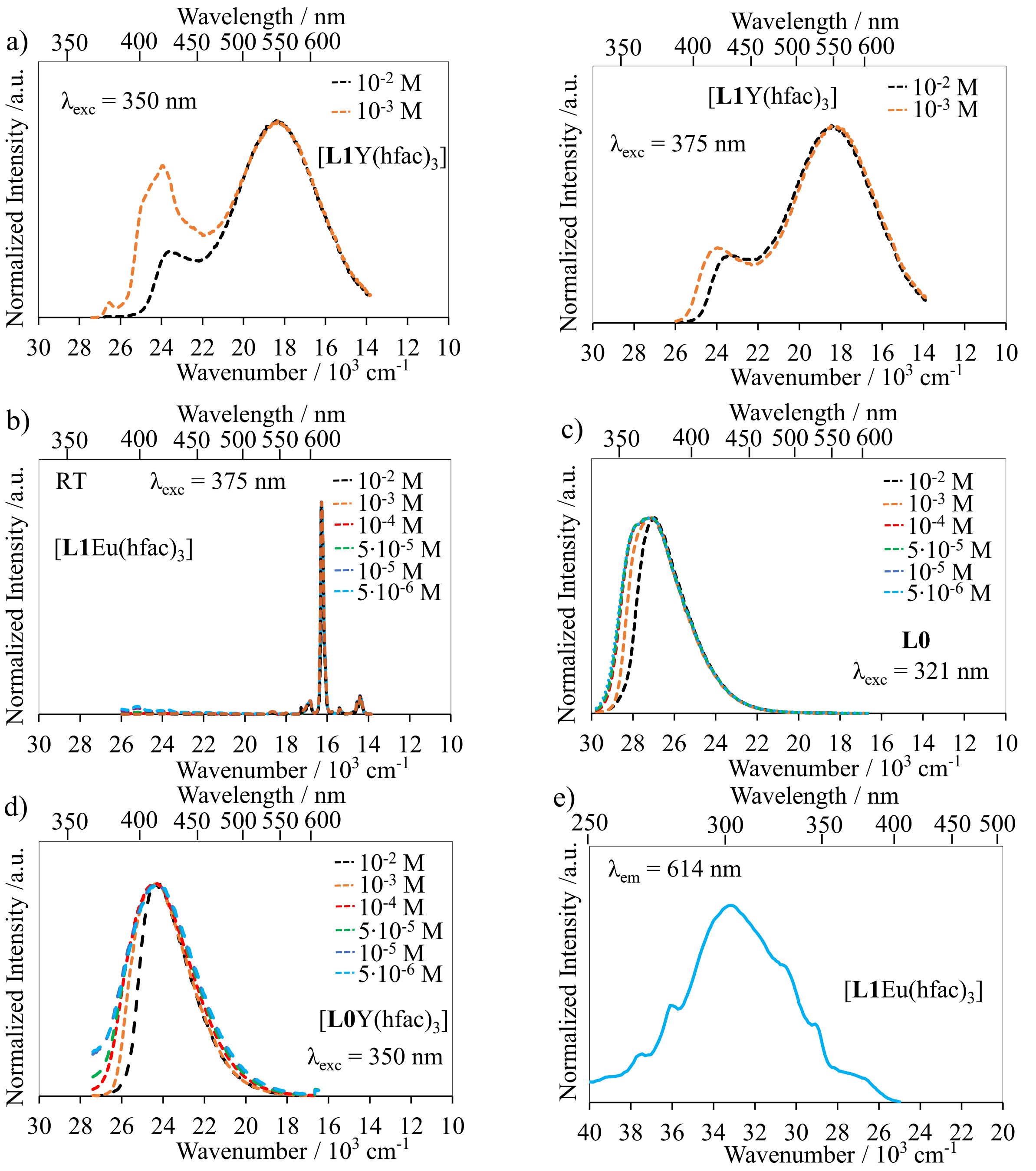

Figure A6-1. Normalized emission spectra recorded for a) [**L1**Y(hfac)$_3$] at λ_{exc} = 350 nm (left) and λ_{exc} = 375 nm (right), b) [**L1**Eu(hfac)$_3$] at λ_{exc} = 350 nm, c) **L0** at λ_{exc} = 321 nm, d) [**L0**Y(hfac)$_3$] at λ_{exc} = 350 nm, and e) excitation spectrum of [**L1**Eu(hfac)$_3$] at λ_{em} = 614 nm (dichloromethane, 293 K).

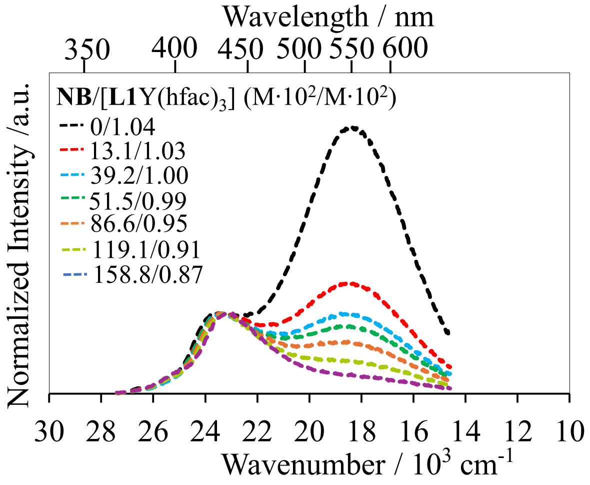

Figure A6-2. Fluorescence titration of [**L1**Y(hfac)$_3$] at 10^{-2} M with NB (λ_{exc} = 350 nm)
